# Influence of androgen deprivation therapy on serum urate levels in patients with prostate cancer: A retrospective observational study

**DOI:** 10.1371/journal.pone.0209049

**Published:** 2018-12-17

**Authors:** Jun Won Park, Jae Hyun Lee, Hyon Joung Cho, You-Jung Ha, Eun Ha Kang, Kichul Shin, Seok-Soo Byun, Eun Young Lee, Yeong Wook Song, Yun Jong Lee

**Affiliations:** 1 Department of Internal Medicine, Seoul National University Hospital, Seoul, Republic of Korea; 2 Department of Internal Medicine, Seoul National University College of Medicine, Seoul, Republic of Korea; 3 Department of Internal Medicine, Armed Forces Capital Hospital, Seongnam, Republic of Korea; 4 Department of Internal Medicine, Seoul National University Bundang Hospital, Seongnam, Republic of Korea; 5 Department of Internal Medicine, Seoul Metropolitan Government–Seoul National University Boramae Medical Center, Seoul, Republic of Korea; 6 Departments of Urology, Seoul National University Bundang Hospital, Seongnam, Republic of Korea; 7 WCU Department of Molecular Medicine and Biopharmaceutical Sciences, Medical Research Institute, Seoul National University College of Medicine, Seoul, Republic of Korea; 8 Department of Translational Medicine, Seoul National University College of Medicine, Seoul, Republic of Korea; University of Toronto, CANADA

## Abstract

**Objectives:**

Although estrogenic modulation of serum urate levels is well-known, the androgenic effect on urate homeostasis remains controversial. We investigated the effect of androgen deprivation therapy (ADT) on serum urate levels.

**Methods:**

We retrospectively enrolled a total of 489 prostate cancer patients with available serum urate levels at baseline and 3 and 6 months after ADT (n = 150) or prostate surgery (n = 339). We extracted the demographic, clinical, and laboratory data from a data warehouse and compared the changes in urate levels between the two treatment groups and between the different ADT regimens (with versus without luteinizing hormone-releasing hormone (LHRH) agonists) using generalized estimating equation (GEE).

**Results:**

The baseline urate levels and the proportion of hyperuricemic subjects were comparable between the two groups. After 6 months, the urate levels were significantly decreased (by −0.66 mg/dL, 95% confidence interval (CI) [-0.81 to -0.51]) in the ADT group, whereas they did not significantly change in the surgery group in the univariate GEE analysis. The ADT group (4.7% from 18.0% at baseline) had a significantly lower proportion of hyperuricemic patients than surgery group (16.5% from 15.9% at baseline) at 6-month (p < 0.001). Regardless of whether LHRH agonists were used, the serial urate levels were lowered by the ADT. Temporal changes in the urate levels were significantly associated with the treatment group, baseline hyperuricemia, and poor functional or advanced cancer status. The ADT-related serum urate level reduction also remained significant in the multivariate GEE analysis (regression coefficient = −0.43 [−0.67 to −0.19] after 3 months and −0.37 [−0.64 to −0.10] after 6 months). Moreover, propensity-score-matched analyses yielded the same results.

**Conclusions:**

Our results showed that longitudinal serum urate levels were significantly reduced in men receiving ADT. This finding suggests that androgen could have an independent role in urate homeostasis.

## Introduction

Gout is a crystal-induced disease caused by the deposition of monosodium urate monohydrate crystals. Hippocrates already noted the association between gout and males and wrote several famous aphorisms describing the characteristics of gout 2500 years ago; one of Hippocrates’ remarks on this disease is that eunuchs do not develop gout and a woman does not have the gout unless menstruation is stopped [1]. Therefore, gout has been considered a male-predominant disease although the known clinical and genetic risk factors for gout are not different by gender, except for the serum urate (SUA) levels [2].

Chronic hyperuricemia is the primary risk factor for gout, and the gender discrepancy in gout prevalence could be explained by the varying SUA levels between men and women. Generally, women have significantly lower SUA levels than age-matched men, which then tend to increase after menopause [3]. In addition, many studies in postmenopausal women or male-to-female transgenders have shown SUA reduction after estrogen treatment [4,5]. Moreover, masculinizing hormone therapy in female-to-male transgender patients suppresses estradiol levels and could elevate the SUA levels [6]. The estrogen-induced increase in the renal clearance of urate has been suggested as a mechanism for the gender discrepancy in the SUA levels [7].

The question of whether the male hormone status could independently affect the SUA levels, as in the case of female hormones, remains to be fully answered. Unfortunately, only four studies have been published concerning the effect of testosterone on the SUA levels in men, and they have reported conflicting results [8–11].

Given that approximately 80% of estradiol is estimated to be produced via testosterone aromatization in men, exogenous testosterone therapy can also elevate the estradiol levels in men [12]. Accordingly, the SUA levels could be affected by an increase in the estrogen as well as androgen levels in testosterone replacement therapy. ADT has been indicated for the treatment of locally advanced or metastatic prostate cancer and includes luteinizing hormone-releasing hormone (LHRH) agonists and/or androgen receptor (AR) antagonists [13]. Chemical castration with LHRH analogues results in a reduction of the endogenous testosterone and estrogen levels, whereas AR antagonist monotherapy leads to their increase in the circulation [14,15]. Based on these findings, the effects of LHRA analogues and AR antagonists on the SUA levels would be different from each other if only estrogen is a single independent modulator of the SUA levels. Therefore, we explored the longitudinal changes in the SUA levels during the first 6 months after ADT in patients with prostate cancer (ADT group) and patients undergoing prostatectomy alone (surgery group) and compared the changes between patients receiving LHRH agonists and those on AR antagonist monotherapy.

## Materials and methods

### Patients and clinical data

**Fig 1:**
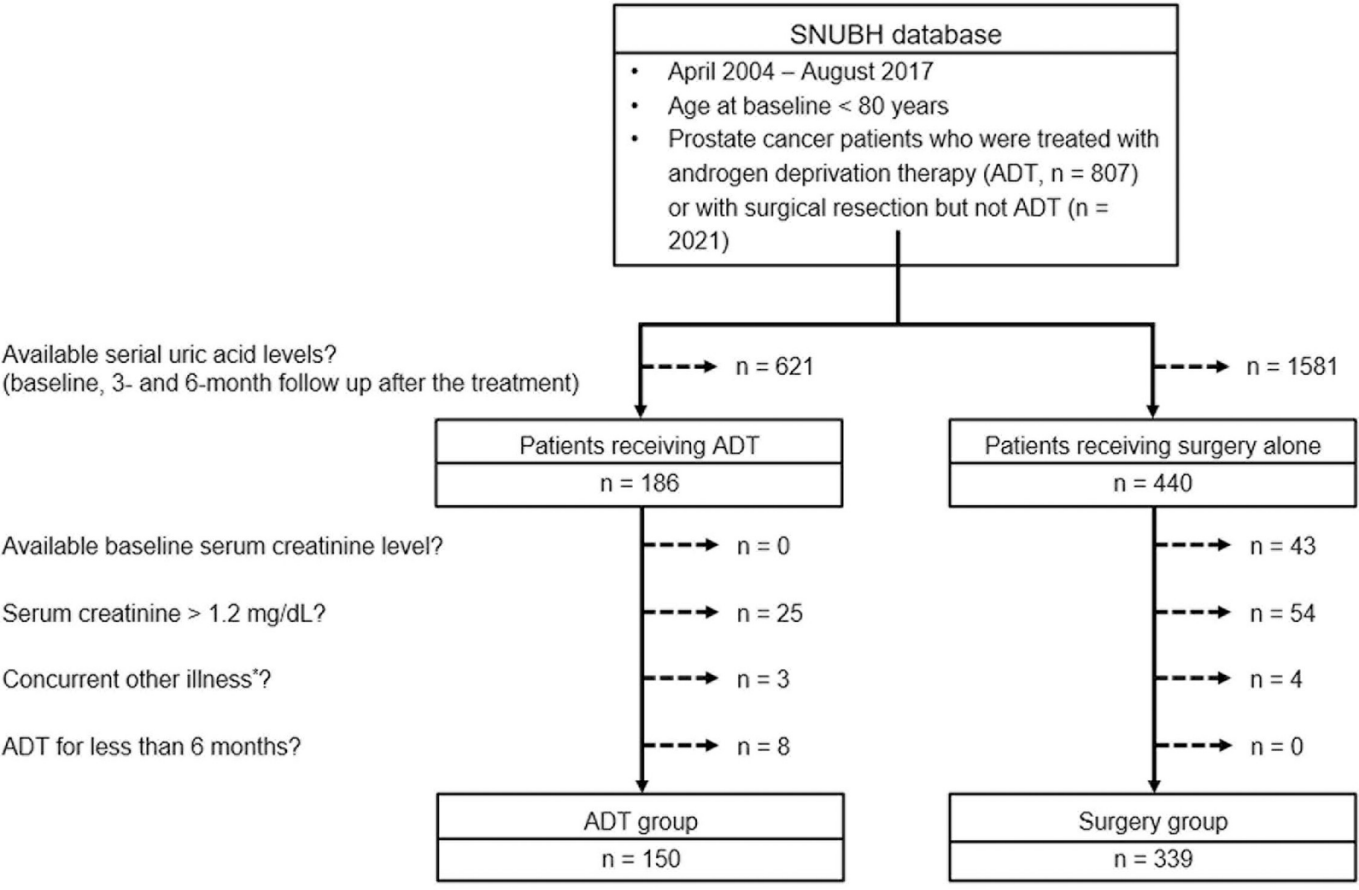
Flowchart for patient inclusion. *, including stomach cancer (n = 2), hepatocellular cancer (1), colon cancer (1), multiple myeloma (1), gastrointestinal stromal tumor (1), and ulcerative colitis (1). ADT, androgen deprivation therapy.

We conducted a retrospective study using the Seoul National University Bundang Hospital’s clinical data warehouse containing electronic medical records and laboratory results. We searched for patients aged 80 years or less, who were diagnosed with prostate cancer between April 2003 and August 2017. Of these patients, we selected those who have available baseline and serial SUA levels and serum creatinine levels ≤1.2 mg/dL at baseline and 12 and 24 weeks after the surgical treatment or ADT for prostate cancer. The baseline SUA level was defined as the SUA within 28 days before the treatment for prostate cancer, and the follow-up SUA levels were collected at 12 ±2 weeks and 24 ±2 weeks after the treatment. We excluded patients who were receiving urate-lowering agents or diagnosed with concurrent other illnesses which could affect the SUA levels during the study period. Additionally, patients with an ADT duration <6 months or those undergoing a surgical treatment during the ADT were excluded (Fig 1).

Finally, 339 patients receiving only surgical prostatectomy (surgery group) and 150 patients with ADT (ADT group) were identified as the study population. In the ADT group, 114 (76.0%) patients received LHRH agonists with or without AR antagonists (LHRH subgroup) and 36 (24.0%) were on monotherapy with AR antagonists (AR subgroup). The demographic and clinical data, including the prostate cancer stage, Eastern Cooperative Oncology Group (ECOG) functional status, co-morbidities, and concomitant medications, were collected from the data warehouse. Additionally, laboratory data including protein, albumin, cholesterol, blood urea nitrogen (BUN), and creatinine were collected to estimate the nutritional or renal status of the patients. Patients were classified into hypouricemic (<4.0 mg/dL), normouricemic, or hyperuricemic (≥7.0 mg/dL) subgroups according to the baseline SUA levels [16,17]. The study data file is available in the electronic supplementary material (S1 File).

This study was carried out in accordance with the Declaration of Helsinki and was approved by the Institutional Review Board (IRB) of Seoul National Bundang University Hospital (IRB no. B-1405/250-102). Informed patient consent was waived by our IRB because this study was retrospective and had a minimal risk to the participants. All data were anonymized prior to the analysis.

### Statistical analysis

Continuous variables were expressed as the mean ±standard deviation (SD). Continuous or dichotomous variables were compared between the ADT and surgery groups with the chi-square test or Student’s t-test, respectively. The longitudinal change in variables, including the SUA levels, was analyzed with generalized estimating equation (GEE) models. To adjust for the within-subject correlation due to the repeated measurements of the SUA levels within the same individual, we used the ‘exchangeable’ correlation structure based on the Pearson correlation coefficient among the measurements. Post-hoc pairwise comparisons of the estimated SUA levels were adjusted using the Bonferroni correction. The effect of two-way interactions between each clinical factor and time on the longitudinal SUA levels was explored, and if a relevant interaction (p <0.1) was found, this interaction was included as a covariate in the final multivariable model. Pearson correlation was performed to test collinearity between covariates. If a correlation coefficient between two covariates was higher than 0.8, one of them was removed from the multivariable models.

Additionally, we created 1:1 matched sample using propensity score matching because an imbalance of the baseline characteristics in the ADT and surgery groups could result in biased results. Propensity scores were calculated from a logistic regression model using age, current alcohol drinking, cancer stage (stage 4 or not), serum nutritional or renal markers such as protein, albumin, BUN and creatinine, baseline SUA level and concomitant aspirin usage. A caliper of 0.2 was used to discard matches showing discordance (S1 Fig).

GEE model analysis was separately conducted for the propensity-score-matched subgroups and for the LHRH versus AR subgroups. All statistical analyses were done with SPSS 20.0 (IBM, USA) and P values <0.05 were considered statistically significant.

## Results

### Baseline characteristics of the study participants

The baseline characteristics of the ADT and surgery groups are summarized in Table 1. Briefly, the ADT group was older and had higher prevalence of current drinker and patients with metastatic cancer or receiving concurrent radiation therapy than that of the surgery group. However, most patients had a favorable performance status and only 5 had an ECOG performance status of 3 to 4. Although the serum creatinine levels were comparable, the ADT group had a higher BUN and lower protein and albumin level than that of the surgery group. The SUA levels (5.8 ±1.3 mg/dL in the ADT group versus 5.7 ±1.4 mg/dL in the surgery group) and the proportion of subjects with hyperuricemia (18.0% in the ADT versus and 15.9% in the surgical group) were not significantly different between the two groups.

**Table 1:** Baseline characteristics of the patients.

	Surgery group (n = 339)	ADT group (n = 150)	P value
Age, year, mean (SD)	66.5 (7.1)	69.2 (7.2)	<0.001
Body mass index, kg/m^2^, mean (SD)	24.5 (2.8)	24.3 (2.5)	0.527
Current alcohol drink*, n (%)	171 (50.4)	54 (37.5)	0.009
Hypertension, n (%)	167 (49.3)	77 (51.3)	0.673
Diabetes mellitus, n (%)	69 (20.4)	28 (18.7)	0.666
Coronary artery disease, n (%)	30 (8.8)	7 (4.7)	0.111
Dyslipidemia, n (%)	90 (26.5)	31 (20.8)	0.176
Concomitant radiotherapy, n (%)	5 (1.5)	73 (48.7)	<0.001
Metastatic prostate cancer, n (%)	2 (0.6)	49 (32.7)	<0.001
ECOG functional status^†^, n (%)			<0.001
0	86 (26.1)	11 (8.0)	
1	135 (41.0)	97 (70.8)	
2	106 (32.2)	26 (19.0)	
3	2 (0.6)	2 (1.5)	
4	0 (0.0)	1 (0.7)	
Serum uric acid, mg/dL, mean (SD)	5.7 (1.4)	5.8 (1.3)	0.633
Hyperuricemia, n (%)	54 (15.9)	27 (18.0)	0.570
Hypouricemia, n (%)	32 (9.4)	12 (8.0)	0.608
Serum protein, mg/dL, mean (SD)	7.2 (0.6)	7.1 (0.6)	0.036
Serum albumin, mg/dL, mean (SD)	4.3 (0.5)	4.1 (0.4)	0.001
Serum cholesterol, mg/dL, mean (SD)	178.3 (35.0)	176.6 (36.0)	0.640
BUN, mg/dL, mean (SD)	15.6 (3.7)	16.3 (4.3)	0.049
Serum creatinine, mg/dL, mean (SD)	0.94 (0.15)	0.95 (0.15)	0.600
Medications			
Aspirin use, n (%)	79 (23.3)	25 (16.7)	0.327
Thiazide, n (%)	45 (13.3)	13 (8.7)	0.153
Loop diuretics, n (%)	3 (0.9)	3 (2.0)	0.302
Angiotensin receptor blockers, n (%)	114 (36.9)	36 (24.2)	0.037
Statins, n (%)	98 (28.9)	36 (24.2)	0.279
Bicalutamide, n (%)		134 (89.3%)	-
Cyproterone, n (%)		2 (1.3%)	-
Leuprorelin, n (%)		39 (26.0%)	-
Goserelin, n (%)		72 (48.0%)	-
Triptorelin, n (%)		3 (2.0%)	-

*, data were missing in 23;

†, data were missing in 6;

ADT, androgen deprivation therapy; BUN, blood urea nitrogen; ECOG, Eastern Cooperative Oncology Group; SD, standard deviation.

In the ADT group, 100 patients received the combination therapy of LHRH agonist and AR antagonist. The most prevalent regimen was the combination of goserelin (63.0%) or leuprorelin (33.0%) with bicalutamide. LHRH agonist monotherapy was done in 14 patents and bicalutamide as a monotherapy was given to 36 patients. In both groups, the post-treatment prostate-specific antigen (PSA) levels significantly decreased after 24 weeks (from 69.2 ±171.1 to 7.1 ±69.6 ng/mL in the ADT group and from 42.9 ±590.0 to 0.03 ±0.06 ng/mL in the surgery group, both p < 0.001).

### Clinical factors associated with the longitudinal SUA change in the univariate analyses

**Fig 2:**
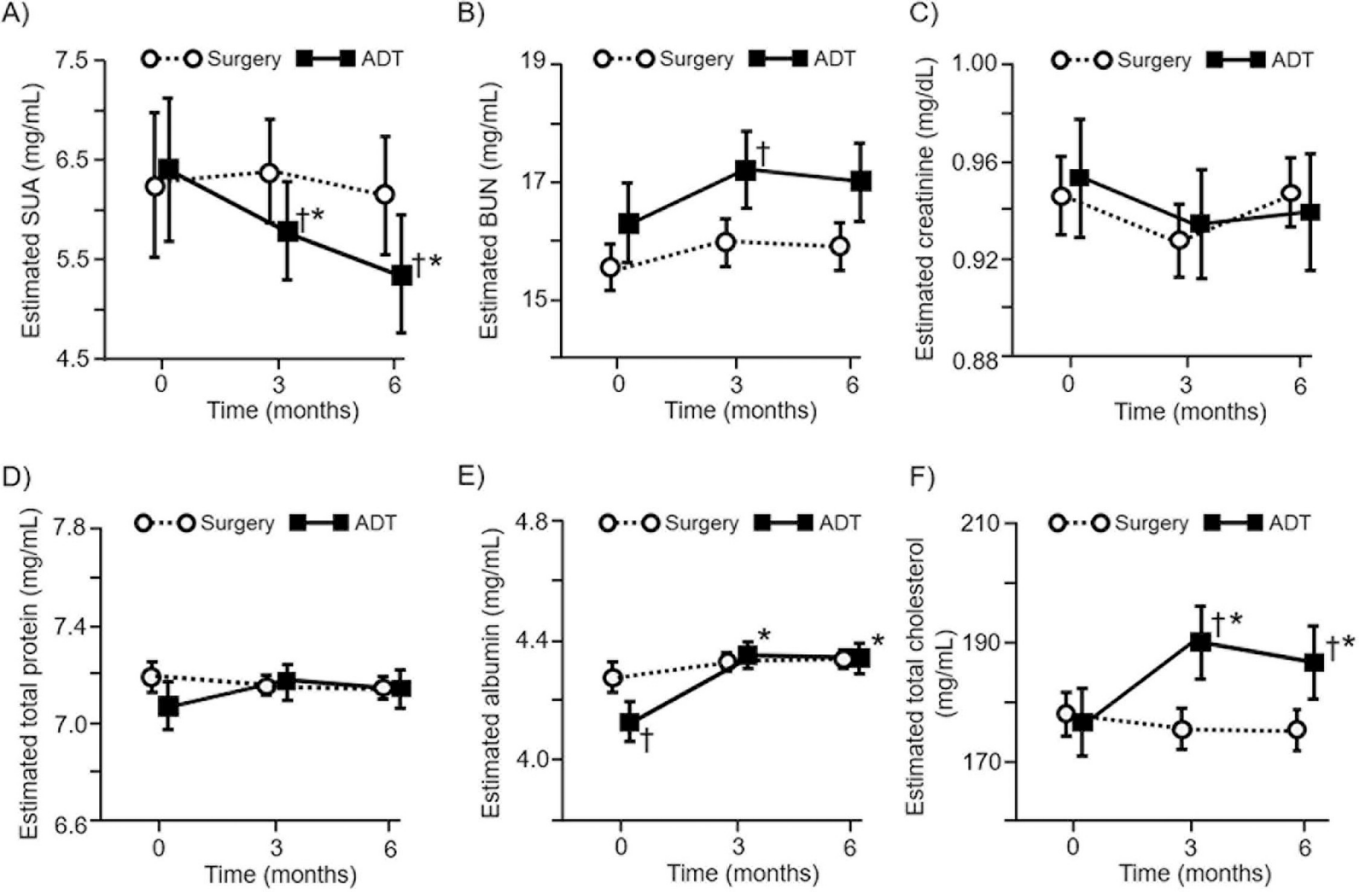
Longitudinal changes in the serum urate (SUA, A), blood urea nitrogen (BUN, B), serum creatinine (C), total protein (D), albumin (E), and total cholesterol (F) levels. P values were corrected by the Bonferroni method. * p <0.05 between the baseline and 6-month time points; †, p <0.05 when compared between the surgery and ADT groups at each time point.

In a univariate GEE analysis, the SUA levels were significantly decreased by 0.66 mg/dL (95% confidence interval (CI) -0.81 to -0.51) after 6 months of the ADT treatment (p <0.001); however, they did not significantly change in the surgery group (β = 0.23, 95% CI -0.03 to 0.48) after 6 months (Fig 2A). During the study period, no patients showed a significant deterioration in the ECOG functional status and renal function (Fig 2B and 2C). Moreover, the levels of nutritional markers such as total protein, cholesterol and albumin did not decrease across the follow-up period in the two groups (Fig 2D–2F). Rather, the serum albumin or total cholesterol levels were significantly increased from the baseline to 6-month in the ADT group (p <0.001 in both analyses). These findings suggest that the longitudinal decrease in the SUA levels in the ADT group was less likely to be associated with nutritional or renal factors.

**Fig 3:**
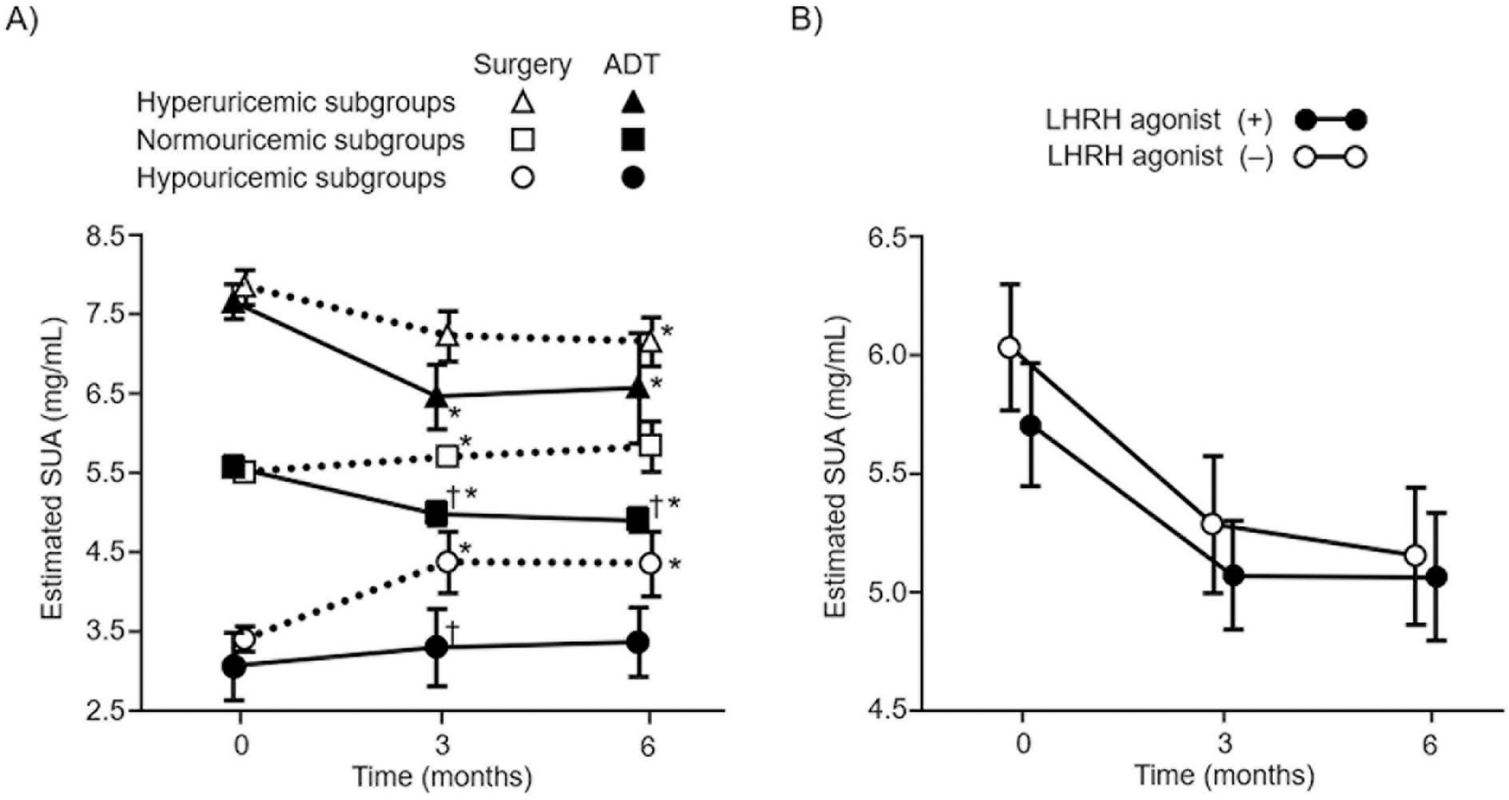
Longitudinal changes in serum urate (SUA) levels according to the subgroups. Hyperuricemia (SUA ≥7.0 mg/dL), normouricemia (4.0≤ and <7.0 mg/dL), and hypouricemia (<4.0 mg/dL) were defined by using the baseline SUA levels. P values were corrected by the Bonferroni method. * p <0.05 between the baseline and 6-month time points; †, p <0.05 when compared between the surgery and ADT groups at each time point.

When patients were stratified into the three subgroups according to their baseline SUA levels, the serial SUA levels significantly decreased in the hyperuricemic and normouricemic patients of the ADT group at the 6-month follow-up (Fig 3A). Consequently, although the proportions of baseline hyperuricemia were comparable between the ADT and surgery groups (18.0% versus 15.9%), the ADT group had a significantly lower proportion of hyperuricemic patients than that of the surgery group (4.7% versus 16.5%, p < 0.001) at 6 months. Additionally, because the hypouricemic patients in the surgery group had a significant increase in the SUA levels, the surgery group had a significantly lower proportion of hypouricemic patients than that of the ADT group (5.9% versus 14.1%, p <0.001) 6 months after the treatment.

When patients in the ADT group were stratified into two subgroups based on LHRH agonist use, the temporal change in the SUA levels was similar between the LHRH and AR subgroup (Fig 3B). The baseline characteristics of the two subgroups are summarized in S1 Table.

In the univariate analyses to detect two-way interactions, the longitudinal SUA levels were significantly influenced by the group (ADT versus surgery), the presence of baseline hyperuricemia or stage 4 prostate cancer, a poor ECOG performance status (score 3 to 4), and concurrent radiotherapy (Table 2). Interestingly, the SUA levels were significantly decreased by 0.81 (95% CI 0.55 to 1.08) mg/dL at the 6-month follow-up in patients with baseline hyperuricemia whereas patients without baseline hyperuricemia did not show a significant change in uric acid level (β = 0.13, 95% CI -0.05 to 0.15). The presence of metastatic prostate cancer or concurrent radiotherapy was also associated with a significant decrease in the SUA level during the entire study period. However, patients with a poor functional status had numerically lower SUA levels at baseline; but the difference in the SUA levels disappeared after 6 months.

**Table 2:** Effect of the interaction between a specific clinical factor and time on the longitudinal serum uric acid (SUA) levels.

	β (95% CI)*	P value^†^	N
Time × group (ADT-control)		<0.001	489
Baseline	0.06 (-0.19 to 0.32)		
3-month	-0.70 (-0.93 to -0.47)		
6-month	-0.82 (-1.16 to -0.49)		
Time × baseline hyperuricemia		<0.001	489
Baseline	2.50 (2.32 to 2.69)		
3-month	1.64 (1.36 to 1.93)		
6-month	1.57 (1.20 to 1.94)		
Time × stage 4 cancer		<0.001	489
Baseline	-0.06 (-0.50 to 0.38)		
3-month	-0.74 (-1.12 to -0.37)		
6-month	-0.73 (-1.23 to -0.23)		
Time × poor functional status^¶^ at baseline		<0.001	466
Baseline	-0.19 (-1.65 to 1.28)		
3-month	-0.60 (-2.06 to 0.86)		
6-month	0.02 (-1.19 to 1.24)		
Time × concurrent radiotherapy		<0.001	488
Baseline	0.08 (-0.23 to 0.38)		
3-month	-0.76 (-1.07 to -0.45)		
6-month	-0.49 (-0.75 to -0.23)		
Time × age		0.649	489
Time × BMI		0.185	489
Time × current alcohol drink		0.206	483
Time × ever-smoking		0.352	480

*, indicates the difference in the SUA levels between the ADT and control groups at a specific time point;

†, p value for the interaction in the type 3 test. If an interaction showed a relevant significance (p <0.1), the differences in the SUA between stratified subgroups at three time points (baseline, 3- and 6-month) were presented;

¶, defined as Eastern Cooperative Oncology Group (ECOG) functional status of 3 or 4; BMI, body mass index; CI, confidence interval.

### Longitudinal serum urate level change in the multivariate analyses

We generated the multivariable GEE models adjusting for time and clinical factors associated with the SUA levels to assess the impact of ADT on the temporal change in the SUA levels. The ADT group had significantly reduced SUA levels by 0.43 mg/dL (95% CI -0.67 to -0.19) at 3 months and by 0.37 mg/dL (95% CI -0.64 to -0.10) at 6 months. In contrast, the SUA levels in the surgery group were increased by 0.27 mg/dL 3 months after the surgical treatment and by 0.39 mg/dL 6 months after the surgical treatment (Table 3).

**Table 3:** Effect of androgen-deprivation therapy (ADT) on the longitudinal serum uric acid (SUA) change in the whole population and post-matched population.

	Adjusted β* (95% CI) at 3-month	Adjusted β* (95% CI) at 6-month
Whole study population^†^ (n = 489)		
ADT group	-0.43 (-0.67 to -0.19)	-0.37 (-0.64 to -0.10)
Surgery group	0.27 (0.16 to 0.37)	0.39 (0.11 to 0.68)
Post-matched population^¶^ (n = 180)		
ADT group	-0.41 (-0.68 to -0.15)	-0.45 (-0.74 to -0.15)
Surgery group	0.38 (0.20 to 0.56)	0.34 (0.16 to 0.51)

*, the difference in the SUA compared with the baseline;

†, multivariable model was adjusted for the interaction between time and relevant clinical factors such as cancer stage, ECOG functional status, concurrent radiotherapy and the presence of baseline hyperuricemia;

¶, multivariable model was adjusted for interaction between time and relevant clinical factors such as ECOG functional status, concurrent radiotherapy, and the presence of baseline hyperuricemia; CI, confidence interval.

### Sensitivity analysis in the propensity score–matched subgroups

The propensity-score–matched analysis included 90 participants in each group (their baseline characteristics are summarized in S2 Table). All the variables used in the propensity score matching were balanced between the two treatment groups. However, although the proportions of hyperuricemic subjects were not different (13.3% in the post-matched surgical subgroup versus 17.8% in the post-matched ADT subgroups), the post-matched ADT subgroup had higher SUA levels than the post-matched surgery subgroup (5.43 ±1.30 versus 5.84 ±1.17 mg/dL, respectively; p = 0.027).

In the multivariable GEE models, the post-matched ADT subgroups had significantly decreased SUA levels by 0.41 (95% CI -0.68 to -0.68) mg/dL after 3 months and by 0.45 (95% CI -0.74 to -0.15) mg/dL after 6 months of ADT (Table 3). As in the total patients undergoing surgical treatment, the post-matched surgery subgroup also showed an increase in the longitudinal SUA levels at 3 and 6 months.

## Discussion

The uricosuric action of estrogen has been suggested to be the mechanism underlying the gender difference in SUA levels or in the prevalence of gout. The SUA levels increases in postmenopausal women or in female-to-male transgender patients, whereas the SUA levels decrease in women taking hormone replacement therapy or in male-to-female transgender patients [3–6]. However, only a few studies have investigated whether androgen could affect urate homeostasis.

Because urate cannot freely cross the cell membrane, specific transporters are required for urate handling in the body. Among the urate transporters, urate transporter 1 (URAT1) in the brush border membrane of the proximal tubule has a key role in the renal reabsorption of urate. The URAT1 expression levels are higher in male mice than in female mice, and they are positively and negatively affected by testosterone and estrogen, respectively [18–20]. Therefore, it is possible that androgen and estrogen are factors that affect the SUA levels independently.

In previous cross-sectional studies, the SUA levels were reported to be significantly elevated in men with androgen deficiency [21,22] and negatively associated with the circulating testosterone levels in men [23–26]. However, Rosen *et al*. showed no difference in the serum testosterone levels between asymptomatic hyperuricemic and normouricemic men [27]. Furthermore, the effect of testosterone manipulation on the SUA levels has been inconsistently reported. Exogenous androgen injection significantly decreased the SUA levels in men with gout or with late-onset hypogonadism [8,9]. However, surgical or medical castration in prostate cancer patients led to a reduction in the SUA level in a small-scale or single-arm study [10,11]. Such inconsistency might arise from different study designs, small sample size, underlying diseases of the study participants, or various testosterone manipulations among the previous studies. The present study shows that the serial SUA levels were significantly reduced in men receiving androgen deprivation, and it could provide evidence for the physiological effect of male sex hormones on urate homeostasis.

The introduction of LHRH agonists results in the achievement of castration levels of testosterone and a decrement in the estrogen levels [28]. Although the administration of the AR antagonists leads to an increase in the endogenous levels of both testosterone and estrogen, AR antagonist monotherapy results in a functional castration in men [29–31]. The current study shows that the SUA levels were significantly decreased after 6 months in both the LHRH and AR subgroups. These results support the idea that post-ADT reduction in the SUA levels could be explained by testosterone deprivation and is independent of estrogen action. Androgen could activate the promoter activity of a human urate transporter URAT1 [19], and ADT may negatively affect the expression of URAT1, a pharmacological target of uricosuric drugs.

In terms of the magnitude of change in the SUA levels, its level was estimated to decrease by approximately 0.4 to 0.5 mg/dL 6 months after ADT, shown in our multivariate analysis and in Nishiyama *et al*. [11]. Additionally, previous studies have revealed that the SUA levels were reduced to a similar extent (0.2 to 0.7 mL/dL) by female hormone replacement in postmenopausal women [32]. However, male-to-female transgenders have shown a higher decrease in the SUA levels (−0.7 and −1.2 mg/dL in Nicholls *et al*.’s and Yahyaoui *et al*.’s studies, respectively) after both androgen deprivation and estrogen supplementation [7,33]. These findings also suggest that androgen could be another important player in sex hormone-mediated urate homeostasis.

Unexpectedly, our study showed that baseline hyperuricemia was significantly associated with the temporal changes of the SUA levels. Because the serial SUA levels were decreased in hyperuricemic subjects of both the ADT and surgery groups (Fig 3A), a change in a modifiable lifestyle factor that leads to hyperuricemia could influence the post-treatment SUA levels. Although the difference in the SUA levels between the ADT and surgery groups did not reach a statistical significance, their changes were greater in the ADT group than those in the surgery group. Moreover, in the normouricemic subgroups, ADT led to a significantly greater reduction in the post-treatment SUA levels when compared to the surgery. A polymorphism of ras-responsive element-binding protein 1 (RREB1), a gene downstream of AR signaling, has been reported to regulate the SUA levels [34]. Several cohort studies have shown that gout increases the risk of benign prostatic hyperplasia and prostate cancer [35,36]. These findings suggest that an underlying mechanistic link may exist among genetic variation in androgen sensitivity, androgen-mediated urate transporter regulation, and the SUA levels.

In the present study, the ADT group had more patients with an advanced cancer stage and poor functional status compared to the surgery group. Anorexia or reduced oral intake is frequently associated with tumor growth [37]. These differences in the baseline characteristics might have an influence on the longitudinal SUA level change. However, the baseline SUA levels were comparable between the ADT and surgery groups, and the serial blood chemistry measurements did not show ongoing deterioration in the nutritional, renal, or disease status. Moreover, in the univariate GEE analysis, the longitudinal change in the SUA level was not associated with that in the cholesterol, BUN, total protein, and albumin levels. Furthermore, the tumor burden was successfully reduced by ADT or surgical treatment in our patients with prostate cancer; the serial PSA levels were significantly suppressed in both groups. Accordingly, advanced cancer stage or nutritional factors could not significantly affect the results of this study.

Our surgery group showed that the longitudinal SUA levels significantly increased across the study period after adjusting for confounders in both the total and propensity score-matched populations. However, in the surgery group, 27 (50.0%) of the 54 hyperuricemia patients were found to be normouricemic after 6 months, and especially, the SUA levels of the hypouricemic subjects were increased over the follow-up period. Thus, such an increment in the surgical group might reflect merely their natural recovery course following surgery for prostate cancer; 18 (56.2%) of the 32 hypouricemic patients at baseline were classified as normouricemia after 6 months. Thus, we believe that the increment has no clinical significance in the surgery group. Higher percentages of current drinkers in the surgery group may explain a temporal increase in the SUA, but we could not collect data on whether patients continued their alcohol use or abstained after the treatment for prostate cancer.

Although we provided evidence that testosterone could independently regulate urate homeostasis, this study has some limitations that should be considered when interpreting the results. First, given the retrospective longitudinal design of the study, our results could be biased by unmeasured confounders or poorly estimated dietary or lifestyle variables. Second, many subjects (77.9%) were excluded due to the unavailability of serial SUA levels although the exclusion rates were comparable between the ADT (77.0%) and surgery (78.2%) groups. These high rates might not be associated with a specific practice pattern of urologists at our institution because regular monitoring of the SUA levels is not necessary during the follow-up care for prostate cancer. Additionally, it may be influenced by the presence of comorbidities or some unmeasured factors such as patients’ compliance to blood test and physicians’ preference in ordering blood chemistry assays. Consequently, the sample size may not be large enough to draw strong conclusions in this study. Third, because of the short follow-up period, we could not evaluate the long-term effect of ADT. Finally, we did not have data on the renal handling of urate during ADT or on the change in the urate transporter expression in the kidney. Therefore, this study could not present an underlying mechanism for the urate-lowering effect of ADT.

In conclusion, our results show that the longitudinal SUA levels were significantly lowered in men undergoing testosterone deprivation, which suggest that androgen could directly affect urate homeostasis independent of estrogen.

## Supporting information

S1 File Data file analyzed in this study. (PDF)

S1 Fig Standardized difference of covariates before and after the propensity score matching. (TIF)

S1 Table Baseline features of luteinizing hormone-releasing hormone (LHRH) and androgen receptor (AR) subgroups. AR subgroup received monotherapy with AR antagonists.
(DOCX)

S2 Table Baseline characteristics of the propensity-score–matched patients. (DOCX)
