# Supplementary material for: Influence of androgen deprivation therapy on serum urate levels in patients with prostate cancer: A retrospective observational study
Source: PLoS One. 2018 Dec 17;13(12):e0209049. doi: 10.1371/journal.pone.0209049 (PMC6296534; doi:10.1371/journal.pone.0209049)
Supplement: S1 File — (PDF) [file pone.0209049.s001.pdf]

Variable	Description
id	Patient's ID
Group	0, surgery group ; 1, ADT group
BMI	BMI
Current_ETOH	Current alcohol drink
Ever_smoke	Ever smokers
HTN	Hypertension
DM	Diabetes mellitus
CAD	Coronary artery disease
DYSLIPID	Dyslipidemia
Stage_4	Stage 4 prostate cancer
ECOG	ECOG status (0-4)
Con_asa	Concomitant aspirin use
Con_thia	Concomitant thiazide use
con_loopD	Concomitant loop diuretics use
con_ARB	Concomitant angiotensin receptor blocker use
con_statin	Concomitant statin use
Anti_Androgen	Anti-androgen use
Bicalutamide	Bicalutamide
Cyproterone	Cyproterone
GNRH	GnRH use
Leuprorelin	Leuprorelin
Goserelin	Goserelin
Triptorelin	Triptorelin
Conc_RT	Concomitant radiotherapy for prostate cancer
Base_UA	Baseline uric acid level
Base_hyperUA	Baseline hyperuricemia
Base_hypoUA	Baseline hypouriceia
time	1 : baseline / 2 : 3 months / 3 : 6 months
UA	SUA level at time point
BUN	BUN level at time point
CR	Serum creatinine level at time point
PROT	Serum protein level at time point
ALB	Serum albumin level at time point
CHOL	Serum cholesterol level at time point

data_ADT_SUA

id	Group	BMI	Current_ETOH	Ever_smoke	HTN	DM	CAD	DYSLIPID	Stage_4	ECOG	Con_asa	Con_thia	con_loopD	con_ARB	con_statin	Anti_Androgen	Bicalutamide	Cyproterone	GNRH	Leuprorelin	Goserelin	Triptorelin	Conc_RT	Base_UA	Base_hyperUA	Base_hypoUA	time	UA	BUN	CR	PROT	ALB	CHOL
1	0	24.3	1	0	0	0	0	1	0	1	0	0	0	0	0	0	0	0	0	0	0	0	0	2.4	0	1	1	2.4	16	1.06	8.0	5.0	205
1	0	24.3	1	0	0	0	0	1	0	1	0	0	0	0	0	0	0	0	0	0	0	0	0	2.4	0	1	2	5.8	22	0.96	6.8	4.2	163
1	0	24.3	1	0	0	0	0	1	0	1	0	0	0	0	0	0	0	0	0	0	0	0	0	2.4	0	1	3	6.1	17	0.87	7.3	4.6	175
2	0	18.8	1	1	0	0	0	0	0	2	0	0	0	0	0	0	0	0	0	0	0	0	0	2.4	0	1	1	2.4	12	0.65	7.0	4.0	156
2	0	18.8	1	1	0	0	0	0	0	2	0	0	0	0	0	0	0	0	0	0	0	0	0	2.4	0	1	2	5.1	16	0.77	7.4	4.3	183
2	0	18.8	1	1	0	0	0	0	0	2	0	0	0	0	0	0	0	0	0	0	0	0	0	2.4	0	1	3	4.1	10	0.82	6.4	3.8	172
3	0	23.6	1	0	0	0	0	0	0	2	0	0	0	0	0	0	0	0	0	0	0	0	0	6.2	0	0	1	6.2	17	1.20	7.0	4.0	173
3	0	23.6	1	0	0	0	0	0	0	2	0	0	0	0	0	0	0	0	0	0	0	0	0	6.2	0	0	2	5.5	21	1.10	7.2	4.3	202
3	0	23.6	1	0	0	0	0	0	0	2	0	0	0	0	0	0	0	0	0	0	0	0	0	6.2	0	0	3	6.0	15	1.00	6.9	4.2	202
4	1	26.9	0	0	0	0	0	0	0	1	0	0	0	0	0	1	1	0	1	1	0	0	1	4.1	0	0	1	4.1	14	1.10	7.0	4.0	218
4	1	26.9	0	0	0	0	0	0	0	1	0	0	0	0	0	1	1	0	1	1	0	0	1	4.1	0	0	2	4.0	20	1.10	6.9	3.8	190
4	1	26.9	0	0	0	0	0	0	0	1	0	0	0	0	0	1	1	0	1	1	0	0	1	4.1	0	0	3	4.1	16	1.20	7.0	3.8	240
5	0	21.6	0	1	0	1	0	1	0	1	0	0	0	0	1	0	0	0	0	0	0	0	0	6.7	0	0	1	6.7	20	0.92	7.0	4.0	131
5	0	21.6	0	1	0	1	0	1	0	1	0	0	0	0	1	0	0	0	0	0	0	0	0	6.7	0	0	2	6.2	17	0.96	7.2	4.5	106
5	0	21.6	0	1	0	1	0	1	0	1	0	0	0	0	1	0	0	0	0	0	0	0	0	6.7	0	0	3	6.1	20	0.96	7.3	4.5	113
6	0	25.9	1	1	0	1	0	0	0	2	1	0	0	0	0	0	0	0	0	0	0	0	0	5.1	0	0	1	5.1	14	0.78	7.0	4.0	182
6	0	25.9	1	1	0	1	0	0	0	2	1	0	0	0	0	0	0	0	0	0	0	0	0	5.1	0	0	2	5.0	15	0.95	6.9	4.2	212
6	0	25.9	1	1	0	1	0	0	0	2	1	0	0	0	0	0	0	0	0	0	0	0	0	5.1	0	0	3	5.1	22	1.07	6.8	4.2	165
7	0	27.3	1	1	0	0	0	0	0	0	0	0	0	0	0	0	0	0	0	0	0	0	0	6.1	0	0	1	6.1	21	0.91	7.0	4.0	161
7	0	27.3	1	1	0	0	0	0	0	0	0	0	0	0	0	0	0	0	0	0	0	0	0	6.1	0	0	2	7.1	25	0.82	7.2	4.3	172
7	0	27.3	1	1	0	0	0	0	0	0	0	0	0	0	0	0	0	0	0	0	0	0	0	6.1	0	0	3	6.0	19	0.86	7.0	4.2	138
8	0	26.1	1	1	1	0	0	0	0	2	0	0	0	1	0	0	0	0	0	0	0	0	0	5.8	0	0	1	5.8	16	1.12	8.0	5.0	160
8	0	26.1	1	1	1	0	0	0	0	2	0	0	0	1	0	0	0	0	0	0	0	0	0	5.8	0	0	2	6.5	19	0.97	6.9	4.1	143
8	0	26.1	1	1	1	0	0	0	0	2	0	0	0	1	0	0	0	0	0	0	0	0	0	5.8	0	0	3	5.7	18	1.08	6.8	4.2	143
9	0	25.6	1	1	0	1	0	1	0	1	1	0	0	0	1	0	0	0	0	0	0	0	0	5.7	0	0	1	5.7	19	1.25	7.0	5.0	130
9	0	25.6	1	1	0	1	0	1	0	1	1	0	0	0	1	0	0	0	0	0	0	0	0	5.7	0	0	2	5.7	20	1.19	7.1	4.5	137
9	0	25.6	1	1	0	1	0	1	0	1	1	0	0	0	1	0	0	0	0	0	0	0	0	5.7	0	0	3	6.2	25	1.12	7.3	4.6	142
10	1	19.2	1	0	0	1	0	0	0	1	1	0	0	0	0	1	1	0	1	0	1	0	1	4.9	0	0	1	4.9	17	1.06	7.0	5.0	175
10	1	19.2	1	0	0	1	0	0	0	1	1	0	0	0	0	1	1	0	1	0	1	0	1	4.9	0	0	2	4.2	18	0.95	7.0	4.5	190
10	1	19.2	1	0	0	1	0	0	0	1	1	0	0	0	0	1	1	0	1	0	1	0	1	4.9	0	0	3	4.6	15	0.95	7.4	5.0	187
11	1	24.0	0	1	1	0	0	0	1	1	1	0	0	1	0	1	1	0	1	0	1	0	0	7.6	1	0	1	7.6	20	0.73	7.0	4.0	216
11	1	24.0	0	1	1	0	0	0	1	1	1	0	0	1	0	1	1	0	1	0	1	0	0	7.6	1	0	2	4.1	22	0.70	7.8	5.0	276
11	1	24.0	0	1	1	0	0	0	1	1	1	0	0	1	0	1	1	0	1	0	1	0	0	7.6	1	0	3	3.8	21	0.64	7.3	4.6	163
12	0	26.4	0	0	0	0	0	0	0	1	0	0	0	0	0	0	0	0	0	0	0	0	0	6.1	0	0	1	6.1	15	0.91	8.0	4.0	208
12	0	26.4	0	0	0	0	0	0	0	1	0	0	0	0	0	0	0	0	0	0	0	0	0	6.1	0	0	2	6.4	14	0.90	7.2	4.0	172
12	0	26.4	0	0	0	0	0	0	0	1	0	0	0	0	0	0	0	0	0	0	0	0	0	6.1	0	0	3	6.8	19	0.87	7.4	4.3	185
13	0	23.6	1	1	0	0	0	1	0	1	0	0	0	0	1	0	0	0	0	0	0	0	0	6.9	0	0	1	6.9	17	1.20	7.0	4.0	193
13	0	23.6	1	1	0	0	0	1	0	1	0	0	0	0	1	0	0	0	0	0	0	0	0	6.9	0	0	2	6.0	22	1.05	7.2	4.7	200
13	0	23.6	1	1	0	0	0	1	0	1	0	0	0	0	1	0	0	0	0	0	0	0	0	6.9	0	0	3	6.3	19	1.08	7.5	4.9	207
14	0	23.3	0	0	0	1	0	0	0	2	1	0	0	0	0	0	0	0	0	0	0	0	0	7.7	1	0	1	7.7	19	1.30	8.0	4.0	178
14	0	23.3	0	0	0	1	0	0	0	2	1	0	0	0	0	0	0	0	0	0	0	0	0	7.7	1	0	2	6.2	17	1.10	7.3	4.0	166
14	0	23.3	0	0	0	1	0	0	0	2	1	0	0	0	0	0	0	0	0	0	0	0	0	7.7	1	0	3	5.9	16	1.10	6.9	4.3	177
15	0	21.0	0	0	0	0	0	0	0	1	0	0	0	0	0	0	0	0	0	0	0	0	0	4.9	0	0	1	4.9	17	0.93	7.0	4.0	180
15	0	21.0	0	0	0	0	0	0	0	1	0	0	0	0	0	0	0	0	0	0	0	0	0	4.9	0	0	2	5.4	14	0.96	7.0	4.4	190
15	0	21.0	0	0	0	0	0	0	0	1	0	0	0	0	0	0	0	0	0	0	0	0	0	4.9	0	0	3	6.0	16	0.88	6.7	4.1	192
16	1	27.1	0	0	1	0	0	1	0	1	0	0	0	0	1	1	1	0	0	0	0	0	0	4.2	0	0	1	4.2	13	0.88	7.0	4.0	159
16	1	27.1	0	0	1	0	0	1	0	1	0	0	0	0	1	1	1	0	0	0	0	0	0	4.2	0	0	2	4.5	14	0.80	6.7	4.2	191
16	1	27.1	0	0	1	0	0	1	0	1	0	0	0	0	1	1	1	0	0	0	0	0	0	4.2	0	0	3	3.9	15	0.70	7.7	4.2	182
17	0	20.2	1	0	0	0	0	0	0	2	0	0	0	0	0	0	0	0	0	0	0	0	0	5.2	0	0	1	5.2	17	1.30	8.0	5.0	242
17	0	20.2	1	0	0	0	0	0	0	2	0	0	0	0	0	0	0	0	0	0	0	0	0	5.2	0	0	2	5.3	17	1.20	7.5	4.5	214

data_ADT_SUA

id	Group	BMI	Current_ETOH	Ever_smoke	HTN	DM	CAD	DYSLIPID	Stage_4	ECOG	Con_asa	Con_thia	con_loopD	con_ARB	con_statin	Anti_Androgen	Bicalutamide	Cyproterone	GNRH	Leuprorelin	Goserelin	Triptorelin	Conc_RT	Base_UA	Base_hyperUA	Base_hypoUA	time	UA	BUN	CR	PROT	ALB	CHOL
17	0	20.2	1	0	0	0	0	0	0	2	0	0	0	0	0	0	0	0	0	0	0	0	0	5.2	0	0	3	5.1	17	1.10	7.5	4.4	186
18	0	24.4	0	1	1	0	0	0	0	2	0	0	0	1	0	0	0	0	0	0	0	0	0	6.4	0	0	1	6.4	13	0.90	7.0	4.0	202
18	0	24.4	0	1	1	0	0	0	0	2	0	0	0	1	0	0	0	0	0	0	0	0	0	6.4	0	0	2	5.5	20	0.91	7.1	4.5	181
18	0	24.4	0	1	1	0	0	0	0	2	0	0	0	1	0	0	0	0	0	0	0	0	0	6.4	0	0	3	5.3	12	0.95	7.2	4.2	194
19	1	23.2	0	1	1	1	0	0	0	1	1	0	0	0	0	1	1	0	1	0	1	0	1	6.6	0	0	1	6.6	11	0.80	7.0	4.0	164
19	1	23.2	0	1	1	1	0	0	0	1	1	0	0	0	0	1	1	0	1	0	1	0	1	6.6	0	0	2	5.0	15	0.70	6.8	4.2	149
19	1	23.2	0	1	1	1	0	0	0	1	1	0	0	0	0	1	1	0	1	0	1	0	1	6.6	0	0	3	4.4	12	0.70	7.6	4.5	186
20	0	27.4	0	1	0	0	0	0	0	2	0	0	0	0	0	0	0	0	0	0	0	0	0	7.2	1	0	1	7.2	19	0.75	6.0	3.0	144
20	0	27.4	0	1	0	0	0	0	0	2	0	0	0	0	0	0	0	0	0	0	0	0	0	7.2	1	0	2	5.9	16	0.68	7.3	4.1	218
20	0	27.4	0	1	0	0	0	0	0	2	0	0	0	0	0	0	0	0	0	0	0	0	0	7.2	1	0	3	6.9	20	0.73	7.7	4.3	216
21	0	29.4	1	1	0	1	0	0	0	1	0	0	0	0	0	0	0	0	0	0	0	0	0	6.6	0	0	1	6.6	19	0.95	7.0	4.0	206
21	0	29.4	1	1	0	1	0	0	0	1	0	0	0	0	0	0	0	0	0	0	0	0	0	6.6	0	0	2	6.6	14	0.87	6.5	4.0	135
21	0	29.4	1	1	0	1	0	0	0	1	0	0	0	0	0	0	0	0	0	0	0	0	0	6.6	0	0	3	8.5	20	0.84	7.3	4.5	197
22	0	25.9	1	1	1	0	0	1	0	1	1	0	0	1	1	0	0	0	0	0	0	0	0	8.3	1	0	1	8.3	14	1.10	7.0	5.0	146
22	0	25.9	1	1	1	0	0	1	0	1	1	0	0	1	1	0	0	0	0	0	0	0	0	8.3	1	0	2	4.7	10	1.00	7.1	4.5	174
22	0	25.9	1	1	1	0	0	1	0	1	1	0	0	1	1	0	0	0	0	0	0	0	0	8.3	1	0	3	5.9	13	1.00	7.2	4.7	156
23	0	25.8	1	0	1	0	0	1	0	2	0	0	0	1	1	0	0	0	0	0	0	0	0	5.8	0	0	1	5.8	19	1.06	7.0	4.0	159
23	0	25.8	1	0	1	0	0	1	0	2	0	0	0	1	1	0	0	0	0	0	0	0	0	5.8	0	0	2	6.3	14	0.81	7.1	4.4	124
23	0	25.8	1	0	1	0	0	1	0	2	0	0	0	1	1	0	0	0	0	0	0	0	0	5.8	0	0	3	6.9	13	0.98	7.3	4.4	171
24	0	24.4	0	0	0	0	0	0	0	1	0	0	0	0	0	0	0	0	0	0	0	0	0	5.9	0	0	1	5.9	15	1.01	7.0	4.0	236
24	0	24.4	0	0	0	0	0	0	0	1	0	0	0	0	0	0	0	0	0	0	0	0	0	5.9	0	0	2	6.4	17	0.98	7.0	4.5	216
24	0	24.4	0	0	0	0	0	0	0	1	0	0	0	0	0	0	0	0	0	0	0	0	0	5.9	0	0	3	5.5	19	0.98	6.9	4.4	231
25	0	26.3	1	1	1	0	0	0	0	2	0	0	0	1	0	0	0	0	0	0	0	0	0	4.0	0	0	1	4.0	16	0.82	7.0	4.0	228
25	0	26.3	1	1	1	0	0	0	0	2	0	0	0	1	0	0	0	0	0	0	0	0	0	4.0	0	0	2	4.0	17	0.85	6.7	4.1	190
25	0	26.3	1	1	1	0	0	0	0	2	0	0	0	1	0	0	0	0	0	0	0	0	0	4.0	0	0	3	4.2	21	0.86	7.7	4.7	224
26	0	27.7	1	0	1	0	0	0	0	1	1	1	0	1	0	0	0	0	0	0	0	0	0	7.4	1	0	1	7.4	21	0.97	7.0	4.0	194
26	0	27.7	1	0	1	0	0	0	0	1	1	1	0	1	0	0	0	0	0	0	0	0	0	7.4	1	0	2	6.8	13	0.85	7.3	4.7	158
26	0	27.7	1	0	1	0	0	0	0	1	1	1	0	1	0	0	0	0	0	0	0	0	0	7.4	1	0	3	6.1	13	0.94	6.8	4.4	142
27	0	24.0	1	0	1	0	0	0	0	0	0	1	0	1	0	0	0	0	0	0	0	0	0	4.9	0	0	1	4.9	23	1.10	7.0	4.0	182
27	0	24.0	1	0	1	0	0	0	0	0	0	1	0	1	0	0	0	0	0	0	0	0	0	4.9	0	0	2	5.6	17	1.10	7.4	4.7	214
27	0	24.0	1	0	1	0	0	0	0	0	0	1	0	1	0	0	0	0	0	0	0	0	0	4.9	0	0	3	5.4	17	0.96	7.1	4.4	173
28	0	28.1	0	0	0	0	0	0	0	1	0	0	0	0	0	0	0	0	0	0	0	0	0	10.2	1	0	1	10.2	11	1.00	8.0	4.0	160
28	0	28.1	0	0	0	0	0	0	0	1	0	0	0	0	0	0	0	0	0	0	0	0	0	10.2	1	0	2	6.0	16	1.00	7.2	4.4	165
28	0	28.1	0	0	0	0	0	0	0	1	0	0	0	0	0	0	0	0	0	0	0	0	0	10.2	1	0	3	5.8	14	1.00	7.5	4.4	189
29	1	24.1	0	1	1	0	0	0	0	1	0	0	0	1	0	1	1	0	1	1	0	0	0	8.2	1	0	1	8.2	16	0.71	7.0	4.0	192
29	1	24.1	0	1	1	0	0	0	0	1	0	0	0	1	0	1	1	0	1	1	0	0	0	8.2	1	0	2	7.3	22	0.73	6.7	4.1	170
29	1	24.1	0	1	1	0	0	0	0	1	0	0	0	1	0	1	1	0	1	1	0	0	0	8.2	1	0	3	6.7	24	0.80	7.1	4.3	170
30	0	21.2	0	1	0	0	0	0	0	0	0	0	0	0	0	0	0	0	0	0	0	0	0	6.2	0	0	1	6.2	23	1.08	7.0	4.0	139
30	0	21.2	0	1	0	0	0	0	0	0	0	0	0	0	0	0	0	0	0	0	0	0	0	6.2	0	0	2	4.9	15	1.06	7.4	4.0	141
30	0	21.2	0	1	0	0	0	0	0	0	0	0	0	0	0	0	0	0	0	0	0	0	0	6.2	0	0	3	6.2	14	1.03	7.6	3.9	150
31	0	25.8	1	0	1	0	0	1	0	1	1	0	0	1	1	0	0	0	0	0	0	0	0	6.5	0	0	1	6.5	11	0.94	7.0	4.0	221
31	0	25.8	1	0	1	0	0	1	0	1	1	0	0	1	1	0	0	0	0	0	0	0	0	6.5	0	0	2	6.3	12	0.72	7.2	4.6	178
31	0	25.8	1	0	1	0	0	1	0	1	1	0	0	1	1	0	0	0	0	0	0	0	0	6.5	0	0	3	6.6	14	0.71	7.3	4.7	180
32	0	22.5	1	1	0	0	0	0	0	3	0	0	0	0	0	0	0	0	0	0	0	0	0	6.5	0	0	1	6.5	10	0.96	7.0	4.0	174
32	0	22.5	1	1	0	0	0	0	0	3	0	0	0	0	0	0	0	0	0	0	0	0	0	6.5	0	0	2	7.8	9	0.89	6.8	3.7	140
32	0	22.5	1	1	0	0	0	0	0	3	0	0	0	0	0	0	0	0	0	0	0	0	0	6.5	0	0	3	8.0	11	0.87	6.6	3.8	167
33	0	23.0	0	0	0	0	0	0	0	1	1	0	0	0	0	0	0	0	0	0	0	0	0	6.8	0	0	1	6.8	16	0.97	7.0	4.0	175
33	0	23.0	0	0	0	0	0	0	0	1	1	0	0	0	0	0	0	0	0	0	0	0	0	6.8	0	0	2	7.4	17	0.87	7.0	4.5	159
33	0	23.0	0	0	0	0	0	0	0	1	1	0	0	0	0	0	0	0	0	0	0	0	0	6.8	0	0	3	6.3	15	0.85	6.8	4.3	142
34	1	26.2	0	0	1	1	0	0	1	2	0	0	0	1	0	1	1	0	1	0	1	0	0	6.2	0	0	1	6.2	32	1.07	7.0	4.0	147

id	Group	BMI	Current_ETOH	Ever_smoke	HTN	DM	CAD	DYSLIPID	Stage_4	ECOG	Con_asa	Con_thia	con_loopD	con_ARB	con_statin	Anti_Androgen	Bicalutamide	Cyproterone	GNRH	Leuprorelin	Goserelin	Triptorelin	Conc_RT	Base_UA	Base_hyperUA	Base_hypoUA	time	UA	BUN	CR	PROT	ALB	CHOL
34	1	26.2	0	0	1	1	0	0	1	2	0	0	0	1	0	1	1	0	1	0	1	0	0	6.2	0	0	2	3.6	19	0.92	7.3	4.6	162
34	1	26.2	0	0	1	1	0	0	1	2	0	0	0	1	0	1	1	0	1	0	1	0	0	6.2	0	0	3	6.3	22	0.93	7.6	4.8	211
35	0	25.7	1	1	1	1	0	1	0	1	1	0	0	0	1	0	0	0	0	0	0	0	0	5.1	0	0	1	5.1	12	0.60	7.0	4.0	134
35	0	25.7	1	1	1	1	0	1	0	1	1	0	0	0	1	0	0	0	0	0	0	0	0	5.1	0	0	2	4.8	15	0.90	6.7	4.3	143
35	0	25.7	1	1	1	1	0	1	0	1	1	0	0	0	1	0	0	0	0	0	0	0	0	5.1	0	0	3	4.3	14	1.10	6.6	4.4	120
36	0	22.4	1	1	0	0	0	0	0	0	0	0	0	0	1	0	0	0	0	0	0	0	0	4.7	0	0	1	4.7	11	0.89	6.0	4.0	147
36	0	22.4	1	1	0	0	0	0	0	0	0	0	0	0	1	0	0	0	0	0	0	0	0	4.7	0	0	2	6.4	21	0.83	7.8	4.8	127
36	0	22.4	1	1	0	0	0	0	0	0	0	0	0	0	1	0	0	0	0	0	0	0	0	4.7	0	0	3	5.7	17	0.77	7.2	4.8	163
37	0	21.9	0	1	0	0	0	0	0	0	0	0	0	0	0	0	0	0	0	0	0	0	0	3.5	0	1	1	3.5	13	0.98	8.0	4.0	151
37	0	21.9	0	1	0	0	0	0	0	0	0	0	0	0	0	0	0	0	0	0	0	0	0	3.5	0	1	2	6.8	13	1.03	6.6	4.0	173
37	0	21.9	0	1	0	0	0	0	0	0	0	0	0	0	0	0	0	0	0	0	0	0	0	3.5	0	1	3	7.4	15	1.09	6.4	4.0	173
38	0	24.4	0	0	0	0	0	0	0	2	1	1	0	0	0	0	0	0	0	0	0	0	0	10.2	1	0	1	10.2	19	1.10	8.0	5.0	200
38	0	24.4	0	0	0	0	0	0	0	2	1	1	0	0	0	0	0	0	0	0	0	0	0	10.2	1	0	2	7.8	11	1.10	7.6	4.5	206
38	0	24.4	0	0	0	0	0	0	0	2	1	1	0	0	0	0	0	0	0	0	0	0	0	10.2	1	0	3	7.5	14	1.00	8.1	4.6	147
39	1	23.1	0	0	1	0	0	1	0	1	0	0	0	0	1	1	1	0	1	1	0	0	1	5.2	0	0	1	5.2	12	0.94	8.0	4.0	258
39	1	23.1	0	0	1	0	0	1	0	1	0	0	0	0	1	1	1	0	1	1	0	0	1	5.2	0	0	2	7.2	21	0.96	7.7	4.2	195
39	1	23.1	0	0	1	0	0	1	0	1	0	0	0	0	1	1	1	0	1	1	0	0	1	5.2	0	0	3	6.8	18	0.82	8.0	4.3	185
40	0	24.4	0	0	0	1	0	1	0	1	0	0	0	0	1	0	0	0	0	0	0	0	0	3.0	0	1	1	3.0	10	0.87	7.0	4.0	191
40	0	24.4	0	0	0	1	0	1	0	1	0	0	0	0	1	0	0	0	0	0	0	0	0	3.0	0	1	2	5.7	9	0.93	7.0	4.2	206
40	0	24.4	0	0	0	1	0	1	0	1	0	0	0	0	1	0	0	0	0	0	0	0	0	3.0	0	1	3	6.0	9	1.05	7.0	4.3	169
41	0	24.5	1	1	0	0	0	0	0	1	0	0	0	0	0	0	0	0	0	0	0	0	0	4.3	0	0	1	4.3	12	0.83	7.0	4.0	191
41	0	24.5	1	1	0	0	0	0	0	1	0	0	0	0	0	0	0	0	0	0	0	0	0	4.3	0	0	2	6.1	14	0.72	7.1	4.4	186
41	0	24.5	1	1	0	0	0	0	0	1	0	0	0	0	0	0	0	0	0	0	0	0	0	4.3	0	0	3	5.8	12	0.72	7.6	4.7	222
42	1	26.9	0	1	0	0	0	0	0	1	0	0	0	0	0	1	1	0	1	1	0	0	1	5.6	0	0	1	5.6	27	1.10	8.0	4.0	204
42	1	26.9	0	1	0	0	0	0	0	1	0	0	0	0	0	1	1	0	1	1	0	0	1	5.6	0	0	2	5.9	18	1.10	7.6	4.7	170
42	1	26.9	0	1	0	0	0	0	0	1	0	0	0	0	0	1	1	0	1	1	0	0	1	5.6	0	0	3	5.9	18	1.00	7.5	4.8	197
43	1	23.9	1	1	0	1	0	0	1	0	0	0	0	0	0	1	1	0	1	1	0	0	0	7.1	1	0	1	7.1	18	1.20	8.0	4.0	188
43	1	23.9	1	1	0	1	0	0	1	0	0	0	0	0	0	1	1	0	1	1	0	0	0	7.1	1	0	2	5.6	20	1.16	7.8	4.6	163
43	1	23.9	1	1	0	1	0	0	1	0	0	0	0	0	0	1	1	0	1	1	0	0	0	7.1	1	0	3	6.5	18	1.16	7.6	4.6	186
44	0	25.0	0	0	1	1	0	1	0	1	1	0	0	1	1	0	0	0	0	0	0	0	0	5.0	0	0	1	5.0	12	0.84	8.0	4.0	152
44	0	25.0	0	0	1	1	0	1	0	1	1	0	0	1	1	0	0	0	0	0	0	0	0	5.0	0	0	2	4.9	16	0.92	7.3	4.1	177
44	0	25.0	0	0	1	1	0	1	0	1	1	0	0	1	1	0	0	0	0	0	0	0	0	5.0	0	0	3	4.8	18	0.81	6.9	4.0	153
45	0	26.0	1	1	1	1	0	1	0	2	0	0	0	1	1	0	0	0	0	0	0	0	0	4.0	0	0	1	4.0	12	1.15	7.0	5.0	131
45	0	26.0	1	1	1	1	0	1	0	2	0	0	0	1	1	0	0	0	0	0	0	0	0	4.0	0	0	2	4.3	12	0.95	7.0	4.3	144
45	0	26.0	1	1	1	1	0	1	0	2	0	0	0	1	1	0	0	0	0	0	0	0	0	4.0	0	0	3	4.0	9	0.83	7.2	4.4	141
46	0	23.5	1	1	0	0	0	0	0	2	0	0	0	0	0	0	0	0	0	0	0	0	0	4.8	0	0	1	4.8	20	0.87	7.0	4.0	195
46	0	23.5	1	1	0	0	0	0	0	2	0	0	0	0	0	0	0	0	0	0	0	0	0	4.8	0	0	2	7.0	23	0.84	7.2	4.3	222
46	0	23.5	1	1	0	0	0	0	0	2	0	0	0	0	0	0	0	0	0	0	0	0	0	4.8	0	0	3	6.1	17	0.83	7.1	4.1	201
47	0	26.9	1	1	1	0	0	0	0	2	0	0	0	0	0	0	0	0	0	0	0	0	0	7.2	1	0	1	7.2	15	0.90	7.0	4.0	223
47	0	26.9	1	1	1	0	0	0	0	2	0	0	0	0	0	0	0	0	0	0	0	0	0	7.2	1	0	2	6.4	15	1.00	7.2	4.2	229
47	0	26.9	1	1	1	0	0	0	0	2	0	0	0	0	0	0	0	0	0	0	0	0	0	7.2	1	0	3	7.4	14	1.10	6.7	4.2	215
48	0	25.1	0	0	1	0	0	0	0	1	0	1	0	1	0	0	0	0	0	0	0	0	0	7.2	1	0	1	7.2	13	0.68	8.0	4.0	223
48	0	25.1	0	0	1	0	0	0	0	1	0	1	0	1	0	0	0	0	0	0	0	0	0	7.2	1	0	2	8.5	19	0.71	7.5	4.1	192
48	0	25.1	0	0	1	0	0	0	0	1	0	1	0	1	0	0	0	0	0	0	0	0	0	7.2	1	0	3	8.5	21	0.72	7.8	4.2	194
49	0	25.5	1	1	1	0	0	1	0	1	0	0	0	1	0	0	0	0	0	0	0	0	0	5.3	0	0	1	5.3	16	1.04	7.0	4.0	179
49	0	25.5	1	1	1	0	0	1	0	1	0	0	0	1	0	0	0	0	0	0	0	0	0	5.3	0	0	2	6.0	13	0.95	7.5	4.4	191
49	0	25.5	1	1	1	0	0	1	0	1	0	0	0	1	0	0	0	0	0	0	0	0	0	5.3	0	0	3	5.7	18	0.94	7.5	4.3	192
50	1	25.5	0	0	1	0	0	0	0	1	0	0	0	1	0	0	0	0	1	0	1	0	0	5.4	0	0	1	5.4	16	1.00	7.0	4.0	172
50	1	25.5	0	0	1	0	0	0	0	1	0	0	0	1	0	0	0	0	1	0	1	0	0	5.4	0	0	2	5.4	16	1.20	7.1	4.5	208
50	1	25.5	0	0	1	0	0	0	0	1	0	0	0	1	0	0	0	0	1	0	1	0	0	5.4	0	0	3	4.7	14	1.20	7.3	4.6	221

id	Group	BMI	Current_ETOH	Ever_smoke	HTN	DM	CAD	DYSLIPID	Stage_4	ECOG	Con_asa	Con_thia	con_loopD	con_ARB	con_statin	Anti_Androgen	Bicalutamide	Cyproterone	GNRH	Leuprorelin	Goserelin	Triptorelin	Conc_RT	Base_UA	Base_hyperUA	Base_hypoUA	time	UA	BUN	CR	PROT	ALB	CHOL
51	0	26.1	0	0	1	1	0	0	0	1	0	1	0	1	0	0	0	0	0	0	0	0	0	5.5	0	0	1	5.5	14	1.20	8.0	5.0	158
51	0	26.1	0	0	1	1	0	0	0	1	0	1	0	1	0	0	0	0	0	0	0	0	0	5.5	0	0	2	6.9	16	1.00	7.7	4.6	181
51	0	26.1	0	0	1	1	0	0	0	1	0	1	0	1	0	0	0	0	0	0	0	0	0	5.5	0	0	3	7.5	18	1.10	7.8	5.0	200
52	1	22.8	0	0	1	0	0	1	0	2	0	0	0	0	1	1	1	0	1	0	1	0	1	3.0	0	1	1	3.0	13	0.98	7.0	5.0	174
52	1	22.8	0	0	1	0	0	1	0	2	0	0	0	0	1	1	1	0	1	0	1	0	1	3.0	0	1	2	3.2	14	0.96	7.5	4.7	174
52	1	22.8	0	0	1	0	0	1	0	2	0	0	0	0	1	1	1	0	1	0	1	0	1	3.0	0	1	3	2.8	14	0.95	7.4	4.7	186
53	0	30.9	0	0	1	1	0	1	0	2	0	0	0	1	1	0	0	0	0	0	0	0	0	3.5	0	1	1	3.5	22	1.00	8.0	5.0	190
53	0	30.9	0	0	1	1	0	1	0	2	0	0	0	1	1	0	0	0	0	0	0	0	0	3.5	0	1	2	5.2	12	0.60	7.5	4.5	152
53	0	30.9	0	0	1	1	0	1	0	2	0	0	0	1	1	0	0	0	0	0	0	0	0	3.5	0	1	3	4.6	14	0.90	7.6	4.5	128
54	0	27.3	1	1	1	0	0	0	0	2	0	0	0	1	0	0	0	0	0	0	0	0	0	3.6	0	1	1	3.6	16	1.20	7.0	4.0	188
54	0	27.3	1	1	1	0	0	0	0	2	0	0	0	1	0	0	0	0	0	0	0	0	0	3.6	0	1	2	4.9	21	1.05	7.2	4.3	180
54	0	27.3	1	1	1	0	0	0	0	2	0	0	0	1	0	0	0	0	0	0	0	0	0	3.6	0	1	3	3.8	14	1.00	7.2	4.2	172
55	1	24.6	0	0	0	1	0	1	0	1	0	0	0	0	1	1	1	0	0	0	0	0	1	4.3	0	0	1	4.3	18	1.00	7.2	4.2	187
55	1	24.6	0	0	0	1	0	1	0	1	0	0	0	0	1	1	1	0	0	0	0	0	1	4.3	0	0	2	4.5	13	0.94	7.1	4.3	160
55	1	24.6	0	0	0	1	0	1	0	1	0	0	0	0	1	1	1	0	0	0	0	0	1	4.3	0	0	3	4.3	20	0.94	7.3	4.4	181
56	0	19.8	0	1	1	0	0	0	0	1	0	0	0	0	0	0	0	0	0	0	0	0	0	5.5	0	0	1	5.5	25	0.82	7.0	4.0	180
56	0	19.8	0	1	1	0	0	0	0	1	0	0	0	0	0	0	0	0	0	0	0	0	0	5.5	0	0	2	6.1	16	0.75	7.1	4.6	181
56	0	19.8	0	1	1	0	0	0	0	1	0	0	0	0	0	0	0	0	0	0	0	0	0	5.5	0	0	3	5.4	20	0.73	7.3	4.7	204
57	1	24.7	0	0	1	0	0	0	0	1	0	0	0	0	0	1	1	0	1	0	1	0	1	4.8	0	0	1	4.8	16	0.75	6.0	4.0	170
57	1	24.7	0	0	1	0	0	0	0	1	0	0	0	0	0	1	1	0	1	0	1	0	1	4.8	0	0	2	5.1	14	0.68	6.3	4.1	181
57	1	24.7	0	0	1	0	0	0	0	1	0	0	0	0	0	1	1	0	1	0	1	0	1	4.8	0	0	3	4.2	14	0.67	6.6	4.4	190
58	1	24.7	0	1	0	0	0	0	1	2	0	0	0	0	0	1	1	0	1	0	1	0	1	7.0	1	0	1	7.0	14	0.82	8.0	4.0	183
58	1	24.7	0	1	0	0	0	0	1	2	0	0	0	0	0	1	1	0	1	0	1	0	1	7.0	1	0	2	8.1	28	0.82	7.6	4.3	238
58	1	24.7	0	1	0	0	0	0	1	2	0	0	0	0	0	1	1	0	1	0	1	0	1	7.0	1	0	3	7.0	17	0.80	7.4	4.1	222
59	0	24.5	0	1	0	0	0	0	0	0	0	0	0	0	0	0	0	0	0	0	0	0	0	5.8	0	0	1	5.8	18	1.06	7.0	4.0	218
59	0	24.5	0	1	0	0	0	0	0	0	0	0	0	0	0	0	0	0	0	0	0	0	0	5.8	0	0	2	7.6	15	0.88	7.5	4.5	229
59	0	24.5	0	1	0	0	0	0	0	0	0	0	0	0	0	0	0	0	0	0	0	0	0	5.8	0	0	3	6.5	13	0.79	7.0	4.2	182
60	0	29.9	0	0	1	0	0	0	0	1	1	0	0	1	0	0	0	0	0	0	0	0	1	4.0	0	0	1	4.0	16	1.00	8.0	4.0	216
60	0	29.9	0	0	1	0	0	0	0	1	1	0	0	1	0	0	0	0	0	0	0	0	1	4.0	0	0	2	4.1	16	0.90	7.2	4.4	219
60	0	29.9	0	0	1	0	0	0	0	1	1	0	0	1	0	0	0	0	0	0	0	0	1	4.0	0	0	3	4.6	20	0.90	6.9	4.3	187
61	0	27.0	1	1	1	0	0	1	0	0	1	0	0	1	1	0	0	0	0	0	0	0	0	5.9	0	0	1	5.9	18	0.97	7.0	4.0	143
61	0	27.0	1	1	1	0	0	1	0	0	1	0	0	1	1	0	0	0	0	0	0	0	0	5.9	0	0	2	5.3	17	1.06	6.8	3.9	141
61	0	27.0	1	1	1	0	0	1	0	0	1	0	0	1	1	0	0	0	0	0	0	0	0	5.9	0	0	3	6.2	20	0.92	6.2	3.6	116
62	0	26.5	0	1	0	0	0	0	0	2	0	0	0	0	0	0	0	0	0	0	0	0	0	7.6	1	0	1	7.6	18	0.96	7.0	5.0	234
62	0	26.5	0	1	0	0	0	0	0	2	0	0	0	0	0	0	0	0	0	0	0	0	0	7.6	1	0	2	4.9	20	1.11	7.7	4.6	214
62	0	26.5	0	1	0	0	0	0	0	2	0	0	0	0	0	0	0	0	0	0	0	0	0	7.6	1	0	3	4.1	22	1.13	7.6	4.1	218
63	0	24.6	0	1	0	0	0	1	0	0	0	0	0	0	1	0	0	0	0	0	0	0	0	6.0	0	0	1	6.0	16	0.95	7.0	4.0	263
63	0	24.6	0	1	0	0	0	1	0	0	0	0	0	0	1	0	0	0	0	0	0	0	0	6.0	0	0	2	7.8	14	0.92	6.5	4.2	206
63	0	24.6	0	1	0	0	0	1	0	0	0	0	0	0	1	0	0	0	0	0	0	0	0	6.0	0	0	3	5.2	15	0.85	6.7	4.5	178
64	0	24.7	1	1	1	0	0	1	0	1	0	0	0	1	1	0	0	0	0	0	0	0	0	3.6	0	1	1	3.6	11	0.88	8.0	4.0	129
64	0	24.7	1	1	1	0	0	1	0	1	0	0	0	1	1	0	0	0	0	0	0	0	0	3.6	0	1	2	2.6	13	0.74	7.5	4.5	142
64	0	24.7	1	1	1	0	0	1	0	1	0	0	0	1	1	0	0	0	0	0	0	0	0	3.6	0	1	3	2.9	18	0.81	7.8	4.6	153
65	0	21.7	0	0	0	0	0	0	0	0	0	0	0	0	0	0	0	0	0	0	0	0	0	4.8	0	0	1	4.8	16	0.87	7.0	4.0	205
65	0	21.7	0	0	0	0	0	0	0	0	0	0	0	0	0	0	0	0	0	0	0	0	0	4.8	0	0	2	4.8	19	0.90	6.6	4.2	186
65	0	21.7	0	0	0	0	0	0	0	0	0	0	0	0	0	0	0	0	0	0	0	0	0	4.8	0	0	3	5.4	25	0.94	6.8	4.4	199
66	0	21.2	0	1	0	0	0	1	0	2	0	0	0	0	1	0	0	0	0	0	0	0	0	6.5	0	0	1	6.5	18	1.00	6.0	4.0	256
66	0	21.2	0	1	0	0	0	1	0	2	0	0	0	0	1	0	0	0	0	0	0	0	0	6.5	0	0	2	5.9	19	1.00	6.3	3.8	206
66	0	21.2	0	1	0	0	0	1	0	2	0	0	0	0	1	0	0	0	0	0	0	0	0	6.5	0	0	3	4.9	14	1.00	6.1	4.0	169
67	0	24.2	0	0	1	0	0	0	0	1	0	1	0	0	0	0	0	0	0	0	0	0	0	7.8	1	0	1	7.8	14	1.03	7.0	4.0	171
67	0	24.2	0	0	1	0	0	0	0	1	0	1	0	0	0	0	0	0	0	0	0	0	0	7.8	1	0	2	8.9	18	0.83	6.7	4.1	160

id	Group	BMI	Current_ETOH	Ever_smoke	HTN	DM	CAD	DYSLIPID	Stage_4	ECOG	Con_asa	Con_thia	con_loopD	con_ARB	con_statin	Anti_Androgen	Bicalutamide	Cyproterone	GNRH	Leuprorelin	Goserelin	Triptorelin	Conc_RT	Base_UA	Base_hyperUA	Base_hypoUA	time	UA	BUN	CR	PROT	ALB	CHOL
67	0	24.2	0	0	1	0	0	0	0	1	0	1	0	0	0	0	0	0	0	0	0	0	0	7.8	1	0	3	9.3	16	0.75	7.0	4.3	178
68	0	24.2	1	1	0	0	0	0	0	1	0	0	0	0	0	0	0	0	0	0	0	0	1	4.2	0	0	1	4.2	15	0.96	7.0	4.0	214
68	0	24.2	1	1	0	0	0	0	0	1	0	0	0	0	0	0	0	0	0	0	0	0	1	4.2	0	0	2	4.6	13	0.91	6.5	4.1	166
68	0	24.2	1	1	0	0	0	0	0	1	0	0	0	0	0	0	0	0	0	0	0	0	1	4.2	0	0	3	4.2	12	0.81	6.9	4.3	180
69	0	21.5	1	1	0	0	0	0	0	1	0	0	0	0	0	0	0	0	0	0	0	0	0	5.5	0	0	1	5.5	15	0.96	8.0	5.0	232
69	0	21.5	1	1	0	0	0	0	0	1	0	0	0	0	0	0	0	0	0	0	0	0	0	5.5	0	0	2	5.4	16	0.87	7.7	4.7	221
69	0	21.5	1	1	0	0	0	0	0	1	0	0	0	0	0	0	0	0	0	0	0	0	0	5.5	0	0	3	5.6	16	0.81	7.4	4.5	188
70	1	25.0	1	0	1	1	0	0	0	1	1	0	0	1	0	1	1	0	1	1	0	0	1	6.3	0	0	1	6.3	17	1.04	7.0	4.0	193
70	1	25.0	1	0	1	1	0	0	0	1	1	0	0	1	0	1	1	0	1	1	0	0	1	6.3	0	0	2	5.1	19	1.03	7.7	4.4	209
70	1	25.0	1	0	1	1	0	0	0	1	1	0	0	1	0	1	1	0	1	1	0	0	1	6.3	0	0	3	4.6	19	1.02	7.3	4.1	220
71	1	24.3	0	1	1	0	0	0	0	1	0	0	1	0	0	1	1	0	0	0	0	0	1	8.0	1	0	1	8.0	16	1.10	7.0	4.0	196
71	1	24.3	0	1	1	0	0	0	0	1	0	0	1	0	0	1	1	0	0	0	0	0	1	8.0	1	0	2	6.7	16	1.00	7.1	4.3	182
71	1	24.3	0	1	1	0	0	0	0	1	0	0	1	0	0	1	1	0	0	0	0	0	1	8.0	1	0	3	5.8	14	1.00	7.1	4.4	194
72	0	26.8	1	0	0	1	0	0	0	2	0	0	0	0	0	0	0	0	0	0	0	0	0	7.6	1	0	1	7.6	18	1.00	9.0	4.0	212
72	0	26.8	1	0	0	1	0	0	0	2	0	0	0	0	0	0	0	0	0	0	0	0	0	7.6	1	0	2	6.5	18	0.90	6.7	3.8	233
72	0	26.8	1	0	0	1	0	0	0	2	0	0	0	0	0	0	0	0	0	0	0	0	0	7.6	1	0	3	6.3	15	0.70	7.3	4.0	187
73	1	22.7	1	1	0	0	0	0	0	1	0	0	0	0	0	0	0	0	1	0	1	0	0	5.4	0	0	1	5.4	15	1.50	8.0	4.0	144
73	1	22.7	1	1	0	0	0	0	0	1	0	0	0	0	0	0	0	0	1	0	1	0	0	5.4	0	0	2	4.8	17	1.10	7.3	4.2	175
73	1	22.7	1	1	0	0	0	0	0	1	0	0	0	0	0	0	0	0	1	0	1	0	0	5.4	0	0	3	4.2	14	1.00	8.0	4.5	180
74	0	17.5	0	0	0	0	0	0	0	2	0	0	0	0	0	0	0	0	0	0	0	0	0	4.7	0	0	1	4.7	18	1.02	7.0	5.0	156
74	0	17.5	0	0	0	0	0	0	0	2	0	0	0	0	0	0	0	0	0	0	0	0	0	4.7	0	0	2	6.5	16	0.91	6.7	4.2	144
74	0	17.5	0	0	0	0	0	0	0	2	0	0	0	0	0	0	0	0	0	0	0	0	0	4.7	0	0	3	5.2	17	0.98	6.6	4.2	138
75	1	21.0	0	1	0	0	0	0	0	1	0	0	0	0	0	1	1	0	1	1	0	0	1	5.6	0	0	1	5.6	12	1.10	7.0	4.0	154
75	1	21.0	0	1	0	0	0	0	0	1	0	0	0	0	0	1	1	0	1	1	0	0	1	5.6	0	0	2	4.6	12	1.10	7.2	4.1	157
75	1	21.0	0	1	0	0	0	0	0	1	0	0	0	0	0	1	1	0	1	1	0	0	1	5.6	0	0	3	4.8	13	1.00	6.6	3.9	154
76	0	24.6	1	1	1	1	0	1	0	2	0	0	0	1	1	0	0	0	0	0	0	0	0	5.2	0	0	1	5.2	15	0.89	8.0	5.0	151
76	0	24.6	1	1	1	1	0	1	0	2	0	0	0	1	1	0	0	0	0	0	0	0	0	5.2	0	0	2	5.2	21	0.81	7.7	4.5	162
76	0	24.6	1	1	1	1	0	1	0	2	0	0	0	1	1	0	0	0	0	0	0	0	0	5.2	0	0	3	4.6	19	0.82	7.5	4.5	153
77	1	22.0	0	0	1	0	0	0	1	1	0	1	0	1	0	1	0	1	1	1	0	0	0	7.0	1	0	1	7.0	14	0.87	8.0	4.0	201
77	1	22.0	0	0	1	0	0	0	1	1	0	1	0	1	0	1	0	1	1	1	0	0	0	7.0	1	0	2	7.3	18	1.03	7.4	4.2	174
77	1	22.0	0	0	1	0	0	0	1	1	0	1	0	1	0	1	0	1	1	1	0	0	0	7.0	1	0	3	6.2	17	0.85	7.0	3.9	170
78	0	21.5	0	1	0	0	0	1	0	2	0	0	0	0	1	0	0	0	0	0	0	0	0	5.7	0	0	1	5.7	13	1.20	8.0	5.0	152
78	0	21.5	0	1	0	0	0	1	0	2	0	0	0	0	1	0	0	0	0	0	0	0	0	5.7	0	0	2	4.8	13	1.20	7.6	4.5	144
78	0	21.5	0	1	0	0	0	1	0	2	0	0	0	0	1	0	0	0	0	0	0	0	0	5.7	0	0	3	6.3	12	1.20	7.3	4.4	173
79	0	29.3	0	0	1	1	0	0	0	2	0	0	0	1	0	0	0	0	0	0	0	0	0	6.3	0	0	1	6.3	17	1.41	7.0	4.0	176
79	0	29.3	0	0	1	1	0	0	0	2	0	0	0	1	0	0	0	0	0	0	0	0	0	6.3	0	0	2	4.7	12	1.09	7.0	4.0	162
79	0	29.3	0	0	1	1	0	0	0	2	0	0	0	1	0	0	0	0	0	0	0	0	0	6.3	0	0	3	5.8	11	1.08	6.7	3.8	144
80	0	19.1	0	1	0	0	0	0	0	2	0	0	0	0	0	0	0	0	0	0	0	0	0	5.4	0	0	1	5.4	17	0.93	7.0	4.0	158
80	0	19.1	0	1	0	0	0	0	0	2	0	0	0	0	0	0	0	0	0	0	0	0	0	5.4	0	0	2	5.5	20	0.98	7.0	4.5	151
80	0	19.1	0	1	0	0	0	0	0	2	0	0	0	0	0	0	0	0	0	0	0	0	0	5.4	0	0	3	5.8	20	0.92	6.9	4.5	162
81	1	21.4	1	1	1	0	0	0	1	1	0	0	1	0	0	1	1	0	1	0	1	0	0	6.3	0	0	1	6.3	20	0.80	7.0	4.0	171
81	1	21.4	1	1	1	0	0	0	1	1	0	0	1	0	0	1	1	0	1	0	1	0	0	6.3	0	0	2	6.3	18	1.10	7.3	4.4	194
81	1	21.4	1	1	1	0	0	0	1	1	0	0	1	0	0	1	1	0	1	0	1	0	0	6.3	0	0	3	5.7	22	0.60	7.6	4.7	190
82	0	25.5	1	1	1	0	0	0	0	1	1	0	0	1	0	0	0	0	0	0	0	0	0	5.8	0	0	1	5.8	19	1.00	7.0	5.0	175
82	0	25.5	1	1	1	0	0	0	0	1	1	0	0	1	0	0	0	0	0	0	0	0	0	5.8	0	0	2	7.4	19	0.90	7.1	4.5	172
82	0	25.5	1	1	1	0	0	0	0	1	1	0	0	1	0	0	0	0	0	0	0	0	0	5.8	0	0	3	5.8	15	0.94	7.1	4.6	159
83	0	25.4	1	1	0	0	0	0	0	1	0	0	0	0	0	0	0	0	0	0	0	0	0	5.7	0	0	1	5.7	9	1.20	7.0	4.0	187
83	0	25.4	1	1	0	0	0	0	0	1	0	0	0	0	0	0	0	0	0	0	0	0	0	5.7	0	0	2	5.2	11	1.00	8.1	4.2	193
83	0	25.4	1	1	0	0	0	0	0	1	0	0	0	0	0	0	0	0	0	0	0	0	0	5.7	0	0	3	6.7	14	1.20	8.0	4.1	179
84	0	25.1	1	1	1	1	0	0	0	2	0	1	0	1	0	0	0	0	0	0	0	0	0	7.6	1	0	1	7.6	18	1.10	8.0	4.0	141

id	Group	BMI	Current_ETOH	Ever_smoke	HTN	DM	CAD	DYSLIPID	Stage_4	ECOG	Con_asa	Con_thia	con_loopD	con_ARB	con_statin	Anti_Androgen	Bicalutamide	Cyproterone	GNRH	Leuprorelin	Goserelin	Triptorelin	Conc_RT	Base_UA	Base_hyperUA	Base_hypoUA	time	UA	BUN	CR	PROT	ALB	CHOL
84	0	25.1	1	1	1	1	0	0	0	2	0	1	0	1	0	0	0	0	0	0	0	0	0	7.6	1	0	2	7.8	25	1.00	7.8	4.2	122
84	0	25.1	1	1	1	1	0	0	0	2	0	1	0	1	0	0	0	0	0	0	0	0	0	7.6	1	0	3	7.6	19	0.90	7.7	4.0	142
85	0	28.2	1	1	0	1	0	1	0	2	0	0	0	0	1	0	0	0	0	0	0	0	0	6.4	0	0	1	6.4	21	1.40	7.0	5.0	219
85	0	28.2	1	1	0	1	0	1	0	2	0	0	0	0	1	0	0	0	0	0	0	0	0	6.4	0	0	2	5.6	18	1.20	7.1	4.6	194
85	0	28.2	1	1	0	1	0	1	0	2	0	0	0	0	1	0	0	0	0	0	0	0	0	6.4	0	0	3	5.8	13	1.10	6.8	4.4	206
86	1	24.9	1	0	1	0	0	0	0	2	0	0	0	1	0	1	1	0	1	0	1	0	0	5.7	0	0	1	5.7	15	1.20	6.0	4.0	172
86	1	24.9	1	0	1	0	0	0	0	2	0	0	0	1	0	1	1	0	1	0	1	0	0	5.7	0	0	2	4.9	14	1.06	6.7	4.2	194
86	1	24.9	1	0	1	0	0	0	0	2	0	0	0	1	0	1	1	0	1	0	1	0	0	5.7	0	0	3	5.7	15	1.05	6.8	4.0	201
87	0	24.2	1	0	0	0	0	0	0	2	0	0	0	0	0	0	0	0	0	0	0	0	0	6.1	0	0	1	6.1	14	1.00	7.0	4.0	194
87	0	24.2	1	0	0	0	0	0	0	2	0	0	0	0	0	0	0	0	0	0	0	0	0	6.1	0	0	2	5.9	14	1.10	7.0	4.2	157
87	0	24.2	1	0	0	0	0	0	0	2	0	0	0	0	0	0	0	0	0	0	0	0	0	6.1	0	0	3	6.3	16	1.00	7.0	4.2	166
88	0	23.4	1	0	1	0	0	0	0	0	1	1	0	1	0	0	0	0	0	0	0	0	0	7.2	1	0	1	7.2	20	0.80	8.0	5.0	205
88	0	23.4	1	0	1	0	0	0	0	0	1	1	0	1	0	0	0	0	0	0	0	0	0	7.2	1	0	2	6.3	14	0.90	7.7	4.7	192
88	0	23.4	1	0	1	0	0	0	0	0	1	1	0	1	0	0	0	0	0	0	0	0	0	7.2	1	0	3	7.0	14	0.90	7.7	4.8	185
89	0	24.1	0	0	1	0	0	0	0	2	0	1	0	1	0	0	0	0	0	0	0	0	0	2.7	0	1	1	2.7	14	1.04	7.0	4.0	206
89	0	24.1	0	0	1	0	0	0	0	2	0	1	0	1	0	0	0	0	0	0	0	0	0	2.7	0	1	2	3.2	14	0.96	6.3	4.1	192
89	0	24.1	0	0	1	0	0	0	0	2	0	1	0	1	0	0	0	0	0	0	0	0	0	2.7	0	1	3	3.4	13	0.93	7.0	4.0	204
90	0	24.3	0	1	1	1	0	0	1	0	0	0	0	1	0	0	0	0	0	0	0	0	0	4.6	0	0	1	4.6	10	1.10	6.0	4.0	123
90	0	24.3	0	1	1	1	0	0	1	0	0	0	0	1	0	0	0	0	0	0	0	0	0	4.6	0	0	2	5.0	13	1.20	6.6	4.1	165
90	0	24.3	0	1	1	1	0	0	1	0	0	0	0	1	0	0	0	0	0	0	0	0	0	4.6	0	0	3	4.5	10	1.00	5.5	3.6	158
91	0	25.3	1	1	1	0	0	0	0	2	0	1	0	1	0	0	0	0	0	0	0	0	0	5.3	0	0	1	5.3	17	1.03	7.0	4.0	190
91	0	25.3	1	1	1	0	0	0	0	2	0	1	0	1	0	0	0	0	0	0	0	0	0	5.3	0	0	2	6.8	17	1.02	7.2	4.5	219
91	0	25.3	1	1	1	0	0	0	0	2	0	1	0	1	0	0	0	0	0	0	0	0	0	5.3	0	0	3	7.2	15	0.99	7.1	4.4	190
92	0	22.9	0	1	0	0	0	0	0	2	0	0	0	0	0	0	0	0	0	0	0	0	0	7.1	1	0	1	7.1	17	0.93	7.0	5.0	256
92	0	22.9	0	1	0	0	0	0	0	2	0	0	0	0	0	0	0	0	0	0	0	0	0	7.1	1	0	2	7.4	17	0.90	6.9	4.6	227
92	0	22.9	0	1	0	0	0	0	0	2	0	0	0	0	0	0	0	0	0	0	0	0	0	7.1	1	0	3	7.6	18	0.79	6.8	4.6	216
93	0	17.0	0	0	0	0	0	0	0	2	0	0	0	0	0	0	0	0	0	0	0	0	0	3.7	0	1	1	3.7	11	0.75	7.0	4.0	132
93	0	17.0	0	0	0	0	0	0	0	2	0	0	0	0	0	0	0	0	0	0	0	0	0	3.7	0	1	2	4.6	11	0.73	6.5	3.5	105
93	0	17.0	0	0	0	0	0	0	0	2	0	0	0	0	0	0	0	0	0	0	0	0	0	3.7	0	1	3	3.2	11	0.71	6.9	3.2	137
94	0	23.4	0	1	1	0	0	1	0	1	1	1	0	1	1	0	0	0	0	0	0	0	0	6.5	0	0	1	6.5	13	1.10	7.0	5.0	126
94	0	23.4	0	1	1	0	0	1	0	1	1	1	0	1	1	0	0	0	0	0	0	0	0	6.5	0	0	2	6.4	12	1.00	7.4	4.7	153
94	0	23.4	0	1	1	0	0	1	0	1	1	1	0	1	1	0	0	0	0	0	0	0	0	6.5	0	0	3	8.2	14	1.00	7.4	4.6	131
95	0	26.3	0	0	0	1	0	1	0	1	1	0	0	0	1	0	0	0	0	0	0	0	0	6.7	0	0	1	6.7	20	1.07	8.0	4.0	136
95	0	26.3	0	0	0	1	0	1	0	1	1	0	0	0	1	0	0	0	0	0	0	0	0	6.7	0	0	2	6.3	20	0.95	7.3	4.5	122
95	0	26.3	0	0	0	1	0	1	0	1	1	0	0	0	1	0	0	0	0	0	0	0	0	6.7	0	0	3	6.8	21	0.96	7.2	4.3	131
96	0	21.5	0	0	1	1	0	1	0	0	1	0	0	0	1	0	0	0	0	0	0	0	0	6.5	0	0	1	6.5	12	1.00	8.0	4.0	148
96	0	21.5	0	0	1	1	0	1	0	0	1	0	0	0	1	0	0	0	0	0	0	0	0	6.5	0	0	2	8.1	15	1.10	7.9	4.5	139
96	0	21.5	0	0	1	1	0	1	0	0	1	0	0	0	1	0	0	0	0	0	0	0	0	6.5	0	0	3	7.8	14	1.00	7.9	4.4	131
97	0	23.0	1	0	0	0	0	0	0	2	0	0	0	0	0	0	0	0	0	0	0	0	0	4.2	0	0	1	4.2	16	1.15	7.0	4.0	208
97	0	23.0	1	0	0	0	0	0	0	2	0	0	0	0	0	0	0	0	0	0	0	0	0	4.2	0	0	2	4.0	16	1.06	6.8	4.2	203
97	0	23.0	1	0	0	0	0	0	0	2	0	0	0	0	0	0	0	0	0	0	0	0	0	4.2	0	0	3	3.9	11	0.93	7.0	4.4	209
98	0	22.1	1	1	1	1	0	1	1	1	0	0	0	1	1	0	0	0	0	0	0	0	0	8.6	1	0	1	8.6	20	1.02	7.0	4.0	234
98	0	22.1	1	1	1	1	0	1	1	1	0	0	0	1	1	0	0	0	0	0	0	0	0	8.6	1	0	2	6.7	19	0.93	7.3	4.3	199
98	0	22.1	1	1	1	1	0	1	1	1	0	0	0	1	1	0	0	0	0	0	0	0	0	8.6	1	0	3	5.9	18	0.91	7.0	4.3	125
99	1	24.6	0	0	0	1	0	1	0	1	0	0	0	0	1	1	1	0	1	0	1	0	1	6.0	0	0	1	6.0	16	0.79	7.0	4.0	136
99	1	24.6	0	0	0	1	0	1	0	1	0	0	0	0	1	1	1	0	1	0	1	0	1	6.0	0	0	2	3.8	15	0.87	7.3	4.6	133
99	1	24.6	0	0	0	1	0	1	0	1	0	0	0	0	1	1	1	0	1	0	1	0	1	6.0	0	0	3	5.1	19	0.94	7.0	4.4	110
100	0	27.6	0	0	0	0	0	0	0	0	0	0	0	0	0	0	0	0	0	0	0	0	0	4.9	0	0	1	4.9	15	1.08	8.0	4.0	189
100	0	27.6	0	0	0	0	0	0	0	0	0	0	0	0	0	0	0	0	0	0	0	0	0	4.9	0	0	2	5.8	15	0.88	7.3	4.4	193
100	0	27.6	0	0	0	0	0	0	0	0	0	0	0	0	0	0	0	0	0	0	0	0	0	4.9	0	0	3	5.8	24	0.85	7.3	4.3	187

id	Group	BMI	Current_ETOH	Ever_smoke	HTN	DM	CAD	DYSLIPID	Stage_4	ECOG	Con_asa	Con_thia	con_loopD	con_ARB	con_statin	Anti_Androgen	Bicalutamide	Cyproterone	GNRH	Leuprorelin	Goserelin	Triptorelin	Conc_RT	Base_UA	Base_hyperUA	Base_hypoUA	time	UA	BUN	CR	PROT	ALB	CHOL
101	0	22.1	1	0	0	0	0	1	0	0	0	0	0	0	1	0	0	0	0	0	0	0	0	4.7	0	0	1	4.7	14	1.20	7.0	4.0	126
101	0	22.1	1	0	0	0	0	1	0	0	0	0	0	0	1	0	0	0	0	0	0	0	0	4.7	0	0	2	6.4	11	1.00	7.5	4.6	205
101	0	22.1	1	0	0	0	0	1	0	0	0	0	0	0	1	0	0	0	0	0	0	0	0	4.7	0	0	3	6.6	15	1.00	7.3	4.2	177
102	0	21.1	0	1	0	0	0	0	0	0	0	0	0	0	0	0	0	0	0	0	0	0	0	5.1	0	0	1	5.1	14	1.30	7.0	4.0	248
102	0	21.1	0	1	0	0	0	0	0	0	0	0	0	0	0	0	0	0	0	0	0	0	0	5.1	0	0	2	6.3	16	1.20	6.5	4.1	181
102	0	21.1	0	1	0	0	0	0	0	0	0	0	0	0	0	0	0	0	0	0	0	0	0	5.1	0	0	3	5.5	15	1.20	6.8	4.3	205
103	1	28.0	0	0	1	1	0	1	0	2	1	0	0	0	1	1	1	0	1	0	1	0	1	6.0	0	0	1	6.0	13	0.76	8.0	4.0	142
103	1	28.0	0	0	1	1	0	1	0	2	1	0	0	0	1	1	1	0	1	0	1	0	1	6.0	0	0	2	4.5	15	0.86	7.9	4.6	170
103	1	28.0	0	0	1	1	0	1	0	2	1	0	0	0	1	1	1	0	1	0	1	0	1	6.0	0	0	3	4.6	14	0.96	7.5	4.4	144
104	0	27.8	0	0	0	0	0	1	0	1	0	0	0	0	1	0	0	0	0	0	0	0	0	6.8	0	0	1	6.8	17	1.10	7.0	4.0	165
104	0	27.8	0	0	0	0	0	1	0	1	0	0	0	0	1	0	0	0	0	0	0	0	0	6.8	0	0	2	7.7	20	0.70	7.3	4.1	163
104	0	27.8	0	0	0	0	0	1	0	1	0	0	0	0	1	0	0	0	0	0	0	0	0	6.8	0	0	3	8.1	16	1.00	6.9	3.9	150
105	1	27.3	0	0	1	0	0	0	0	1	0	0	0	0	0	1	1	0	0	0	0	0	1	7.6	1	0	1	7.6	22	1.11	7.0	4.0	201
105	1	27.3	0	0	1	0	0	0	0	1	0	0	0	0	0	1	1	0	0	0	0	0	1	7.6	1	0	2	5.3	23	1.14	7.3	4.4	237
105	1	27.3	0	0	1	0	0	0	0	1	0	0	0	0	0	1	1	0	0	0	0	0	1	7.6	1	0	3	6.0	22	1.02	6.8	4.4	231
106	1	27.7	1	1	0	0	0	1	0	1	0	0	0	0	0	1	1	0	0	0	0	0	1	4.0	0	0	1	4.0	13	0.68	7.0	4.0	190
106	1	27.7	1	1	0	0	0	1	0	1	0	0	0	0	0	1	1	0	0	0	0	0	1	4.0	0	0	2	3.5	16	0.70	7.1	4.5	169
106	1	27.7	1	1	0	0	0	1	0	1	0	0	0	0	0	1	1	0	0	0	0	0	1	4.0	0	0	3	3.7	13	0.60	7.4	4.4	209
107	0	25.6	0	1	1	0	0	0	0	2	1	0	0	0	0	0	0	0	0	0	0	0	0	5.9	0	0	1	5.9	16	1.10	7.0	4.0	210
107	0	25.6	0	1	1	0	0	0	0	2	1	0	0	0	0	0	0	0	0	0	0	0	0	5.9	0	0	2	6.6	14	1.10	7.3	4.5	219
107	0	25.6	0	1	1	0	0	0	0	2	1	0	0	0	0	0	0	0	0	0	0	0	0	5.9	0	0	3	6.3	14	1.20	7.1	4.4	229
108	1	23.7	0	0	0	0	0	0	0	1	0	0	0	0	0	1	1	0	0	0	0	0	1	5.2	0	0	1	5.2	17	1.20	7.0	4.0	268
108	1	23.7	0	0	0	0	0	0	0	1	0	0	0	0	0	1	1	0	0	0	0	0	1	5.2	0	0	2	4.6	21	1.20	6.8	4.4	217
108	1	23.7	0	0	0	0	0	0	0	1	0	0	0	0	0	1	1	0	0	0	0	0	1	5.2	0	0	3	4.6	21	1.10	6.4	4.2	190
109	0	23.9	1	1	0	0	0	0	0	0	0	0	0	0	0	0	0	0	0	0	0	0	0	6.8	0	0	1	6.8	17	0.96	7.0	5.0	249
109	0	23.9	1	1	0	0	0	0	0	0	0	0	0	0	0	0	0	0	0	0	0	0	0	6.8	0	0	2	6.0	14	1.15	7.0	4.5	205
109	0	23.9	1	1	0	0	0	0	0	0	0	0	0	0	0	0	0	0	0	0	0	0	0	6.8	0	0	3	6.1	15	1.19	7.0	4.4	211
110	0	25.4	0	0	1	0	0	0	0	2	0	0	0	1	0	0	0	0	0	0	0	0	0	6.0	0	0	1	6.0	18	1.00	7.0	5.0	186
110	0	25.4	0	0	1	0	0	0	0	2	0	0	0	1	0	0	0	0	0	0	0	0	0	6.0	0	0	2	6.4	14	0.97	7.3	4.7	192
110	0	25.4	0	0	1	0	0	0	0	2	0	0	0	1	0	0	0	0	0	0	0	0	0	6.0	0	0	3	6.3	15	0.95	7.0	4.5	170
111	0	30.9	1	1	1	0	0	0	0	1	0	1	0	1	0	0	0	0	0	0	0	0	0	5.0	0	0	1	5.0	18	0.90	8.0	5.0	196
111	0	30.9	1	1	1	0	0	0	0	1	0	1	0	1	0	0	0	0	0	0	0	0	0	5.0	0	0	2	5.2	12	1.00	7.1	4.7	193
111	0	30.9	1	1	1	0	0	0	0	1	0	1	0	1	0	0	0	0	0	0	0	0	0	5.0	0	0	3	5.2	14	1.10	7.3	4.7	193
112	0	22.1	0	1	1	0	0	0	0	2	0	0	0	0	0	0	0	0	0	0	0	0	0	5.4	0	0	1	5.4	14	0.99	7.0	4.0	175
112	0	22.1	0	1	1	0	0	0	0	2	0	0	0	0	0	0	0	0	0	0	0	0	0	5.4	0	0	2	5.7	19	0.96	7.0	4.3	169
112	0	22.1	0	1	1	0	0	0	0	2	0	0	0	0	0	0	0	0	0	0	0	0	0	5.4	0	0	3	6.1	12	0.90	7.0	4.2	174
113	0	25.7	1	0	1	0	0	0	0	1	0	0	0	1	0	0	0	0	0	0	0	0	0	7.3	1	0	1	7.3	20	1.16	8.0	4.0	223
113	0	25.7	1	0	1	0	0	0	0	1	0	0	0	1	0	0	0	0	0	0	0	0	0	7.3	1	0	2	6.1	14	1.04	7.5	4.5	175
113	0	25.7	1	0	1	0	0	0	0	1	0	0	0	1	0	0	0	0	0	0	0	0	0	7.3	1	0	3	6.4	14	1.00	7.1	4.4	180
114	0	28.2	1	1	1	0	0	0	0	1	1	0	0	0	0	0	0	0	0	0	0	0	0	5.8	0	0	1	5.8	19	1.12	7.0	4.0	146
114	0	28.2	1	1	1	0	0	0	0	1	1	0	0	0	0	0	0	0	0	0	0	0	0	5.8	0	0	2	4.8	16	1.09	7.1	4.1	147
114	0	28.2	1	1	1	0	0	0	0	1	1	0	0	0	0	0	0	0	0	0	0	0	0	5.8	0	0	3	5.7	12	1.02	7.1	4.2	153
115	1	27.4	1	0	0	0	0	1	1	1	0	0	0	0	1	1	1	0	1	0	1	0	0	5.7	0	0	1	5.7	16	0.87	7.0	4.0	198
115	1	27.4	1	0	0	0	0	1	1	1	0	0	0	0	1	1	1	0	1	0	1	0	0	5.7	0	0	2	5.6	21	0.89	7.7	4.6	253
115	1	27.4	1	0	0	0	0	1	1	1	0	0	0	0	1	1	1	0	1	0	1	0	0	5.7	0	0	3	4.7	15	0.92	7.5	4.5	150
116	1	25.7	0	0	0	0	0	1	0	1	0	0	0	0	1	1	1	0	1	0	1	0	0	6.5	0	0	1	6.5	18	0.89	8.0	4.0	198
116	1	25.7	0	0	0	0	0	1	0	1	0	0	0	0	1	1	1	0	1	0	1	0	0	6.5	0	0	2	6.0	21	0.96	7.3	4.3	214
116	1	25.7	0	0	0	0	0	1	0	1	0	0	0	0	1	1	1	0	1	0	1	0	0	6.5	0	0	3	5.6	21	0.99	7.5	4.5	211
117	1	28.6	1	0	1	0	0	1	0	3	0	0	0	1	1	1	1	0	1	0	0	1	0	6.4	0	0	1	6.4	20	1.10	7.0	4.0	189
117	1	28.6	1	0	1	0	0	1	0	3	0	0	0	1	1	1	1	0	1	0	0	1	0	6.4	0	0	2	5.8	23	1.10	7.6	4.6	243

id	Group	BMI	Current_ETOH	Ever_smoke	HTN	DM	CAD	DYSLIPID	Stage_4	ECOG	Con_asa	Con_thia	con_loopD	con_ARB	con_statin	Anti_Androgen	Bicalutamide	Cyproterone	GNRH	Leuprorelin	Goserelin	Triptorelin	Conc_RT	Base_UA	Base_hyperUA	Base_hypoUA	time	UA	BUN	CR	PROT	ALB	CHOL
117	1	28.6	1	0	1	0	0	1	0	3	0	0	0	1	1	1	1	0	1	0	0	1	0	6.4	0	0	3	4.7	18	0.70	6.9	4.4	214
118	0	20.9	0	0	1	1	0	1	0	1	0	0	0	0	1	0	0	0	0	0	0	0	1	3.8	0	1	1	3.8	24	1.10	7.0	4.0	122
118	0	20.9	0	0	1	1	0	1	0	1	0	0	0	0	1	0	0	0	0	0	0	0	1	3.8	0	1	2	5.3	25	1.20	7.4	4.2	143
118	0	20.9	0	0	1	1	0	1	0	1	0	0	0	0	1	0	0	0	0	0	0	0	1	3.8	0	1	3	5.1	27	1.10	7.0	4.1	153
119	0	23.9	0	1	1	0	0	0	0	1	0	1	0	0	0	0	0	0	0	0	0	0	0	5.6	0	0	1	5.6	12	0.74	7.0	4.0	230
119	0	23.9	0	1	1	0	0	0	0	1	0	1	0	0	0	0	0	0	0	0	0	0	0	5.6	0	0	2	4.3	11	0.71	6.9	4.4	159
119	0	23.9	0	1	1	0	0	0	0	1	0	1	0	0	0	0	0	0	0	0	0	0	0	5.6	0	0	3	4.2	11	0.60	6.7	4.3	143
120	1	18.4	0	1	0	0	0	0	0	1	0	0	0	0	0	1	1	0	1	0	1	0	1	4.2	0	0	1	4.2	14	0.89	6.0	4.0	149
120	1	18.4	0	1	0	0	0	0	0	1	0	0	0	0	0	1	1	0	1	0	1	0	1	4.2	0	0	2	4.8	12	0.94	7.2	4.1	159
120	1	18.4	0	1	0	0	0	0	0	1	0	0	0	0	0	1	1	0	1	0	1	0	1	4.2	0	0	3	5.2	12	0.95	7.4	4.1	164
121	0	25.9	0	0	1	1	0	1	0	1	1	1	0	1	1	0	0	0	0	0	0	0	0	4.0	0	0	1	4.0	29	1.26	7.0	4.0	146
121	0	25.9	0	0	1	1	0	1	0	1	1	1	0	1	1	0	0	0	0	0	0	0	0	4.0	0	0	2	4.6	20	1.10	6.9	4.2	170
121	0	25.9	0	0	1	1	0	1	0	1	1	1	0	1	1	0	0	0	0	0	0	0	0	4.0	0	0	3	4.7	23	1.09	7.5	4.6	173
122	0	24.4	0	0	1	0	0	0	0	0	1	1	0	0	0	0	0	0	0	0	0	0	0	6.5	0	0	1	6.5	19	1.16	7.0	5.0	215
122	0	24.4	0	0	1	0	0	0	0	0	1	1	0	0	0	0	0	0	0	0	0	0	0	6.5	0	0	2	7.9	23	1.20	7.3	4.8	254
122	0	24.4	0	0	1	0	0	0	0	0	1	1	0	0	0	0	0	0	0	0	0	0	0	6.5	0	0	3	8.4	24	1.09	7.2	4.6	240
123	0	27.1	0	1	1	1	0	0	0	2	1	0	0	1	0	0	0	0	0	0	0	0	0	3.1	0	1	1	3.1	11	0.84	7.0	5.0	191
123	0	27.1	0	1	1	1	0	0	0	2	1	0	0	1	0	0	0	0	0	0	0	0	0	3.1	0	1	2	2.7	13	0.83	7.1	4.5	211
123	0	27.1	0	1	1	1	0	0	0	2	1	0	0	1	0	0	0	0	0	0	0	0	0	3.1	0	1	3	3.3	10	0.67	7.3	4.8	219
124	1	25.0	1	0	0	0	0	0	1	1	0	0	0	0	0	1	0	1	1	0	1	0	1	6.5	0	0	1	6.5	21	1.26	7.0	5.0	198
124	1	25.0	1	0	0	0	0	0	1	1	0	0	0	0	0	1	0	1	1	0	1	0	1	6.5	0	0	2	6.0	19	1.05	6.3	4.1	109
124	1	25.0	1	0	0	0	0	0	1	1	0	0	0	0	0	1	0	1	1	0	1	0	1	6.5	0	0	3	4.8	16	1.05	6.4	4.2	131
125	0	20.9	1	0	1	0	0	0	0	2	0	0	0	0	0	0	0	0	0	0	0	0	0	2.8	0	1	1	2.8	13	1.30	8.0	5.0	183
125	0	20.9	1	0	1	0	0	0	0	2	0	0	0	0	0	0	0	0	0	0	0	0	0	2.8	0	1	2	3.3	14	1.10	6.8	4.1	168
125	0	20.9	1	0	1	0	0	0	0	2	0	0	0	0	0	0	0	0	0	0	0	0	0	2.8	0	1	3	2.7	16	1.10	6.7	4.0	166
126	0	25.2	1	0	0	0	0	0	0	0	0	0	0	0	0	0	0	0	0	0	0	0	0	6.4	0	0	1	6.4	15	1.03	8.0	5.0	244
126	0	25.2	1	0	0	0	0	0	0	0	0	0	0	0	0	0	0	0	0	0	0	0	0	6.4	0	0	2	4.8	23	1.01	7.0	4.5	200
126	0	25.2	1	0	0	0	0	0	0	0	0	0	0	0	0	0	0	0	0	0	0	0	0	6.4	0	0	3	5.8	15	0.96	7.4	4.6	225
127	0	25.4	0	0	0	0	0	0	0	0	0	0	0	0	0	0	0	0	0	0	0	0	0	6.0	0	0	1	6.0	17	1.00	7.0	5.0	171
127	0	25.4	0	0	0	0	0	0	0	0	0	0	0	0	0	0	0	0	0	0	0	0	0	6.0	0	0	2	6.2	17	1.00	8.0	4.9	180
127	0	25.4	0	0	0	0	0	0	0	0	0	0	0	0	0	0	0	0	0	0	0	0	0	6.0	0	0	3	6.3	12	0.90	7.3	4.5	158
128	1	23.6	0	0	1	0	0	0	1	1	0	0	0	0	0	1	1	0	1	0	1	0	1	5.4	0	0	1	5.4	29	0.96	7.0	4.0	172
128	1	23.6	0	0	1	0	0	0	1	1	0	0	0	0	0	1	1	0	1	0	1	0	1	5.4	0	0	2	4.8	33	0.96	7.1	4.1	166
128	1	23.6	0	0	1	0	0	0	1	1	0	0	0	0	0	1	1	0	1	0	1	0	1	5.4	0	0	3	4.0	22	0.90	6.6	4.0	168
129	0	23.9	1	0	0	0	0	0	0	2	0	0	0	0	0	0	0	0	0	0	0	0	0	7.9	1	0	1	7.9	13	1.20	7.0	4.0	223
129	0	23.9	1	0	0	0	0	0	0	2	0	0	0	0	0	0	0	0	0	0	0	0	0	7.9	1	0	2	6.3	11	1.10	7.0	4.3	242
129	0	23.9	1	0	0	0	0	0	0	2	0	0	0	0	0	0	0	0	0	0	0	0	0	7.9	1	0	3	7.4	16	1.06	6.8	4.2	251
130	0	26.4	1	1	0	0	0	0	0	1	0	0	0	0	0	0	0	0	0	0	0	0	0	6.6	0	0	1	6.6	11	0.90	8.0	5.0	176
130	0	26.4	1	1	0	0	0	0	0	1	0	0	0	0	0	0	0	0	0	0	0	0	0	6.6	0	0	2	7.8	12	1.00	6.9	4.3	150
130	0	26.4	1	1	0	0	0	0	0	1	0	0	0	0	0	0	0	0	0	0	0	0	0	6.6	0	0	3	7.4	12	1.00	7.4	4.7	143
131	1	28.9	0	0	1	0	0	0	0	2	0	0	0	0	0	1	1	0	1	0	1	0	1	8.2	1	0	1	8.2	14	1.10	7.0	4.0	182
131	1	28.9	0	0	1	0	0	0	0	2	0	0	0	0	0	1	1	0	1	0	1	0	1	8.2	1	0	2	7.7	12	1.10	7.3	4.6	205
131	1	28.9	0	0	1	0	0	0	0	2	0	0	0	0	0	1	1	0	1	0	1	0	1	8.2	1	0	3	8.1	12	1.10	7.1	4.4	201
132	0	26.6	1	0	1	0	0	0	0	0	0	1	0	1	0	0	0	0	0	0	0	0	0	8.1	1	0	1	8.1	16	0.97	8.0	5.0	221
132	0	26.6	1	0	1	0	0	0	0	0	0	1	0	1	0	0	0	0	0	0	0	0	0	8.1	1	0	2	6.6	13	0.98	6.9	4.3	172
132	0	26.6	1	0	1	0	0	0	0	0	0	1	0	1	0	0	0	0	0	0	0	0	0	8.1	1	0	3	7.3	13	1.01	7.2	4.4	212
133	0	23.6	0	0	1	0	0	0	0	1	0	0	0	0	0	0	0	0	0	0	0	0	0	5.3	0	0	1	5.3	12	1.20	8.0	5.0	143
133	0	23.6	0	0	1	0	0	0	0	1	0	0	0	0	0	0	0	0	0	0	0	0	0	5.3	0	0	2	7.2	16	1.20	8.2	4.6	173
133	0	23.6	0	0	1	0	0	0	0	1	0	0	0	0	0	0	0	0	0	0	0	0	0	5.3	0	0	3	6.4	13	1.10	7.9	4.6	149
134	0	24.1	1	0	1	1	0	0	0	1	1	1	0	1	0	0	0	0	0	0	0	0	0	4.9	0	0	1	4.9	18	0.85	7.0	4.0	188

data_ADT_SUA

id	Group	BMI	Current_ETOH	Ever_smoke	HTN	DM	CAD	DYSLIPID	Stage_4	ECOG	Con_asa	Con_thia	con_loopD	con_ARB	con_statin	Anti_Androgen	Bicalutamide	Cyproterone	GNRH	Leuprorelin	Goserelin	Triptorelin	Conc_RT	Base_UA	Base_hyperUA	Base_hypoUA	time	UA	BUN	CR	PROT	ALB	CHOL
134	0	24.1	1	0	1	1	0	0	0	1	1	1	0	1	0	0	0	0	0	0	0	0	0	4.9	0	0	2	5.3	26	0.88	6.6	3.9	151
134	0	24.1	1	0	1	1	0	0	0	1	1	1	0	1	0	0	0	0	0	0	0	0	0	4.9	0	0	3	4.0	16	0.82	6.8	4.1	165
135	0	23.3	1	0	0	0	0	0	0	0	0	0	0	0	0	0	0	0	0	0	0	0	0	5.8	0	0	1	5.8	17	0.87	8.0	5.0	183
135	0	23.3	1	0	0	0	0	0	0	0	0	0	0	0	0	0	0	0	0	0	0	0	0	5.8	0	0	2	5.7	15	0.79	7.9	4.7	207
135	0	23.3	1	0	0	0	0	0	0	0	0	0	0	0	0	0	0	0	0	0	0	0	0	5.8	0	0	3	5.6	15	0.74	7.9	4.3	204
136	0	25.5	0	0	0	0	0	0	0	1	0	0	0	0	0	0	0	0	0	0	0	0	0	4.2	0	0	1	4.2	12	1.10	7.0	5.0	180
136	0	25.5	0	0	0	0	0	0	0	1	0	0	0	0	0	0	0	0	0	0	0	0	0	4.2	0	0	2	4.7	15	1.10	7.1	4.6	197
136	0	25.5	0	0	0	0	0	0	0	1	0	0	0	0	0	0	0	0	0	0	0	0	0	4.2	0	0	3	4.9	13	1.00	6.7	4.4	170
137	1	22.4	1	0	0	1	0	0	0	1	1	0	0	0	0	1	1	0	0	0	0	0	1	5.5	0	0	1	5.5	19	1.30	6.0	4.0	177
137	1	22.4	1	0	0	1	0	0	0	1	1	0	0	0	0	1	1	0	0	0	0	0	1	5.5	0	0	2	5.3	23	1.20	6.2	3.9	175
137	1	22.4	1	0	0	1	0	0	0	1	1	0	0	0	0	1	1	0	0	0	0	0	1	5.5	0	0	3	4.8	15	1.10	6.5	3.9	178
138	0	24.5	0	1	1	1	0	0	0	1	0	0	0	1	0	0	0	0	0	0	0	0	0	4.1	0	0	1	4.1	15	1.10	7.0	4.0	125
138	0	24.5	0	1	1	1	0	0	0	1	0	0	0	1	0	0	0	0	0	0	0	0	0	4.1	0	0	2	3.8	21	1.07	6.7	3.9	134
138	0	24.5	0	1	1	1	0	0	0	1	0	0	0	1	0	0	0	0	0	0	0	0	0	4.1	0	0	3	3.3	18	0.95	6.8	4.0	91
139	1	26.9	1	1	1	0	0	0	0	1	0	0	0	0	0	1	1	0	0	0	0	0	1	4.9	0	0	1	4.9	15	1.11	7.0	4.0	180
139	1	26.9	1	1	1	0	0	0	0	1	0	0	0	0	0	1	1	0	0	0	0	0	1	4.9	0	0	2	4.7	12	1.05	7.3	4.5	198
139	1	26.9	1	1	1	0	0	0	0	1	0	0	0	0	0	1	1	0	0	0	0	0	1	4.9	0	0	3	4.2	23	0.97	6.9	4.2	208
140	0	23.4	0	1	0	0	0	0	0	0	1	0	0	0	0	0	0	0	0	0	0	0	0	6.4	0	0	1	6.4	22	1.00	7.0	4.0	240
140	0	23.4	0	1	0	0	0	0	0	0	1	0	0	0	0	0	0	0	0	0	0	0	0	6.4	0	0	2	6.7	20	1.10	6.9	4.3	253
140	0	23.4	0	1	0	0	0	0	0	0	1	0	0	0	0	0	0	0	0	0	0	0	0	6.4	0	0	3	7.4	18	1.20	7.2	4.2	228
141	0	22.9	1	1	0	0	0	0	0	2	0	0	0	0	0	0	0	0	0	0	0	0	0	4.5	0	0	1	4.5	16	1.11	6.0	4.0	189
141	0	22.9	1	1	0	0	0	0	0	2	0	0	0	0	0	0	0	0	0	0	0	0	0	4.5	0	0	2	5.7	18	1.18	6.4	4.1	166
141	0	22.9	1	1	0	0	0	0	0	2	0	0	0	0	0	0	0	0	0	0	0	0	0	4.5	0	0	3	6.1	18	1.11	6.6	4.1	178
142	0	21.9	1	1	0	0	0	0	0	1	0	0	0	0	0	0	0	0	0	0	0	0	0	6.3	0	0	1	6.3	14	0.86	8.0	5.0	176
142	0	21.9	1	1	0	0	0	0	0	1	0	0	0	0	0	0	0	0	0	0	0	0	0	6.3	0	0	2	6.2	12	0.83	7.2	4.8	169
142	0	21.9	1	1	0	0	0	0	0	1	0	0	0	0	0	0	0	0	0	0	0	0	0	6.3	0	0	3	6.2	14	0.79	7.4	4.9	158
143	0	25.1	0	0	0	0	0	0	0	1	0	0	0	0	0	0	0	0	0	0	0	0	0	5.8	0	0	1	5.8	14	1.05	8.0	5.0	222
143	0	25.1	0	0	0	0	0	0	0	1	0	0	0	0	0	0	0	0	0	0	0	0	0	5.8	0	0	2	6.6	14	1.01	7.6	4.5	180
143	0	25.1	0	0	0	0	0	0	0	1	0	0	0	0	0	0	0	0	0	0	0	0	0	5.8	0	0	3	5.8	21	1.02	7.7	4.7	186
144	1	23.3	0	1	0	0	0	0	0	1	0	0	0	0	0	1	1	0	1	1	0	0	0	5.1	0	0	1	5.1	15	0.77	7.0	4.0	224
144	1	23.3	0	1	0	0	0	0	0	1	0	0	0	0	0	1	1	0	1	1	0	0	0	5.1	0	0	2	3.9	24	0.70	7.3	4.6	244
144	1	23.3	0	1	0	0	0	0	0	1	0	0	0	0	0	1	1	0	1	1	0	0	0	5.1	0	0	3	4.6	21	0.68	7.7	4.8	261
145	1	24.1	0	1	1	0	0	0	1	2	0	0	0	1	0	1	1	0	1	0	1	0	0	6.3	0	0	1	6.3	18	0.99	7.0	4.0	170
145	1	24.1	0	1	1	0	0	0	1	2	0	0	0	1	0	1	1	0	1	0	1	0	0	6.3	0	0	2	5.5	18	0.99	6.8	4.4	182
145	1	24.1	0	1	1	0	0	0	1	2	0	0	0	1	0	1	1	0	1	0	1	0	0	6.3	0	0	3	5.8	16	0.98	7.0	4.4	179
146	0	24.6	1	0	0	0	0	0	0	1	0	0	0	0	0	0	0	0	0	0	0	0	0	5.1	0	0	1	5.1	10	1.05	7.0	5.0	181
146	0	24.6	1	0	0	0	0	0	0	1	0	0	0	0	0	0	0	0	0	0	0	0	0	5.1	0	0	2	5.6	13	1.08	6.9	4.5	170
146	0	24.6	1	0	0	0	0	0	0	1	0	0	0	0	0	0	0	0	0	0	0	0	0	5.1	0	0	3	4.7	12	1.04	6.6	4.4	160
147	0	23.9	1	1	1	0	0	0	0	2	0	0	0	1	0	0	0	0	0	0	0	0	0	3.9	0	1	1	3.9	13	1.10	7.0	4.0	180
147	0	23.9	1	1	1	0	0	0	0	2	0	0	0	1	0	0	0	0	0	0	0	0	0	3.9	0	1	2	4.3	11	1.20	7.0	4.3	152
147	0	23.9	1	1	1	0	0	0	0	2	0	0	0	1	0	0	0	0	0	0	0	0	0	3.9	0	1	3	3.5	13	1.00	6.6	4.2	178
148	1	24.4	0	1	0	1	0	1	0	1	0	0	0	0	0	1	1	0	1	1	0	0	1	6.0	0	0	1	6.0	24	1.16	8.0	4.0	134
148	1	24.4	0	1	0	1	0	1	0	1	0	0	0	0	0	1	1	0	1	1	0	0	1	6.0	0	0	2	5.4	18	0.89	8.1	4.4	196
148	1	24.4	0	1	0	1	0	1	0	1	0	0	0	0	0	1	1	0	1	1	0	0	1	6.0	0	0	3	4.7	20	0.85	7.6	3.9	185
149	0	32.5	1	0	1	0	0	0	0	1	1	0	0	1	0	0	0	0	0	0	0	0	0	6.1	0	0	1	6.1	16	1.20	8.0	5.0	179
149	0	32.5	1	0	1	0	0	0	0	1	1	0	0	1	0	0	0	0	0	0	0	0	0	6.1	0	0	2	5.1	12	1.10	7.3	4.6	148
149	0	32.5	1	0	1	0	0	0	0	1	1	0	0	1	0	0	0	0	0	0	0	0	0	6.1	0	0	3	5.3	14	1.10	7.4	4.6	184
150	0	22.9	0	0	1	0	0	1	0	2	0	0	0	0	1	0	0	0	0	0	0	0	0	5.0	0	0	1	5.0	19	0.77	7.0	4.0	197
150	0	22.9	0	0	1	0	0	1	0	2	0	0	0	0	1	0	0	0	0	0	0	0	0	5.0	0	0	2	5.0	13	0.68	7.3	4.5	154
150	0	22.9	0	0	1	0	0	1	0	2	0	0	0	0	1	0	0	0	0	0	0	0	0	5.0	0	0	3	4.7	25	0.67	6.5	4.1	141

id	Group	BMI	Current_ETOH	Ever_smoke	HTN	DM	CAD	DYSLIPID	Stage_4	ECOG	Con_asa	Con_thia	con_loopD	con_ARB	con_statin	Anti_Androgen	Bicalutamide	Cyproterone	GNRH	Leuprorelin	Goserelin	Triptorelin	Conc_RT	Base_UA	Base_hyperUA	Base_hypoUA	time	UA	BUN	CR	PROT	ALB	CHOL
151	0	26.5	1	0	1	0	0	0	0	1	1	0	0	0	0	0	0	0	0	0	0	0	0	5.3	0	0	1	5.3	13	1.00	7.0	4.0	213
151	0	26.5	1	0	1	0	0	0	0	1	1	0	0	0	0	0	0	0	0	0	0	0	0	5.3	0	0	2	5.6	13	0.89	7.2	4.2	226
151	0	26.5	1	0	1	0	0	0	0	1	1	0	0	0	0	0	0	0	0	0	0	0	0	5.3	0	0	3	5.6	16	0.93	7.4	4.3	208
152	1	22.5	0	0	1	0	0	1	0	1	0	0	0	0	1	1	1	0	1	0	1	0	1	6.1	0	0	1	6.1	17	0.84	7.0	5.0	246
152	1	22.5	0	0	1	0	0	1	0	1	0	0	0	0	1	1	1	0	1	0	1	0	1	6.1	0	0	2	5.1	20	0.74	7.2	4.6	174
152	1	22.5	0	0	1	0	0	1	0	1	0	0	0	0	1	1	1	0	1	0	1	0	1	6.1	0	0	3	5.7	18	0.73	7.3	4.5	211
153	1	23.5	1	0	1	1	0	1	1	2	1	0	0	0	1	1	1	0	1	0	1	0	0	5.4	0	0	1	5.4	18	1.02	7.0	4.0	137
153	1	23.5	1	0	1	1	0	1	1	2	1	0	0	0	1	1	1	0	1	0	1	0	0	5.4	0	0	2	4.9	16	1.01	7.9	4.9	180
153	1	23.5	1	0	1	1	0	1	1	2	1	0	0	0	1	1	1	0	1	0	1	0	0	5.4	0	0	3	4.8	20	0.94	7.1	4.3	130
154	0	24.0	1	0	0	0	0	0	0	1	0	0	0	0	0	0	0	0	0	0	0	0	0	6.3	0	0	1	6.3	15	1.20	7.0	4.0	176
154	0	24.0	1	0	0	0	0	0	0	1	0	0	0	0	0	0	0	0	0	0	0	0	0	6.3	0	0	2	7.8	15	0.99	7.3	4.7	173
154	0	24.0	1	0	0	0	0	0	0	1	0	0	0	0	0	0	0	0	0	0	0	0	0	6.3	0	0	3	8.0	21	0.90	6.9	4.4	160
155	0	27.1	0	0	0	0	0	0	0	0	0	0	0	0	0	0	0	0	0	0	0	0	0	7.9	1	0	1	7.9	15	1.20	8.0	5.0	248
155	0	27.1	0	0	0	0	0	0	0	0	0	0	0	0	0	0	0	0	0	0	0	0	0	7.9	1	0	2	7.5	13	1.20	7.0	4.4	219
155	0	27.1	0	0	0	0	0	0	0	0	0	0	0	0	0	0	0	0	0	0	0	0	0	7.9	1	0	3	7.6	13	1.10	7.6	4.6	252
156	0	21.0	1	1	0	0	0	0	0	0	0	0	0	0	0	0	0	0	0	0	0	0	0	5.6	0	0	1	5.6	12	0.77	7.0	5.0	182
156	0	21.0	1	1	0	0	0	0	0	0	0	0	0	0	0	0	0	0	0	0	0	0	0	5.6	0	0	2	4.9	12	0.79	7.2	4.6	184
156	0	21.0	1	1	0	0	0	0	0	0	0	0	0	0	0	0	0	0	0	0	0	0	0	5.6	0	0	3	4.3	12	0.85	6.8	4.3	142
157	0	29.3	1	0	1	0	0	0	0	0	0	1	0	1	0	0	0	0	0	0	0	0	0	3.9	0	1	1	3.9	15	1.43	7.0	4.0	201
157	0	29.3	1	0	1	0	0	0	0	0	0	1	0	1	0	0	0	0	0	0	0	0	0	3.9	0	1	2	4.0	20	#NULL!	6.6	4.1	191
157	0	29.3	1	0	1	0	0	0	0	0	0	1	0	1	0	0	0	0	0	0	0	0	0	3.9	0	1	3	4.4	20	#NULL!	7.0	4.3	196
158	0	23.6	1	1	0	0	0	0	0	0	0	0	0	0	0	0	0	0	0	0	0	0	0	4.7	0	0	1	4.7	14	0.90	8.0	5.0	188
158	0	23.6	1	1	0	0	0	0	0	0	0	0	0	0	0	0	0	0	0	0	0	0	0	4.7	0	0	2	6.4	12	1.00	7.5	4.5	233
158	0	23.6	1	1	0	0	0	0	0	0	0	0	0	0	0	0	0	0	0	0	0	0	0	4.7	0	0	3	6.1	11	1.10	7.4	4.4	240
159	0	21.8	1	1	0	0	0	0	0	1	0	0	0	0	0	0	0	0	0	0	0	0	0	3.6	0	1	1	3.6	17	0.83	7.0	4.0	182
159	0	21.8	1	1	0	0	0	0	0	1	0	0	0	0	0	0	0	0	0	0	0	0	0	3.6	0	1	2	4.7	14	0.80	7.6	4.3	214
159	0	21.8	1	1	0	0	0	0	0	1	0	0	0	0	0	0	0	0	0	0	0	0	0	3.6	0	1	3	4.0	15	0.75	7.5	4.3	197
160	1	20.0	0	0	1	0	0	0	1	2	1	0	0	0	0	1	1	0	1	0	1	0	0	5.1	0	0	1	5.1	16	1.14	7.0	4.0	152
160	1	20.0	0	0	1	0	0	0	1	2	1	0	0	0	0	1	1	0	1	0	1	0	0	5.1	0	0	2	4.6	23	0.96	7.1	4.3	157
160	1	20.0	0	0	1	0	0	0	1	2	1	0	0	0	0	1	1	0	1	0	1	0	0	5.1	0	0	3	5.3	21	0.94	7.0	4.2	153
161	0	25.6	1	0	1	0	0	0	0	1	1	1	0	0	0	0	0	0	0	0	0	0	0	4.8	0	0	1	4.8	10	0.99	7.0	4.0	236
161	0	25.6	1	0	1	0	0	0	0	1	1	1	0	0	0	0	0	0	0	0	0	0	0	4.8	0	0	2	6.3	14	0.80	7.0	4.3	195
161	0	25.6	1	0	1	0	0	0	0	1	1	1	0	0	0	0	0	0	0	0	0	0	0	4.8	0	0	3	5.4	15	0.79	7.3	4.4	235
162	0	23.1	0	0	0	0	0	0	0	1	0	0	0	0	0	0	0	0	0	0	0	0	0	5.1	0	0	1	5.1	9	1.00	7.0	5.0	149
162	0	23.1	0	0	0	0	0	0	0	1	0	0	0	0	0	0	0	0	0	0	0	0	0	5.1	0	0	2	5.5	8	0.90	7.2	4.7	154
162	0	23.1	0	0	0	0	0	0	0	1	0	0	0	0	0	0	0	0	0	0	0	0	0	5.1	0	0	3	5.3	17	0.90	7.1	4.6	106
163	0	22.2	0	1	0	0	0	1	0	0	1	0	0	1	1	0	0	0	0	0	0	0	0	7.2	1	0	1	7.2	13	1.20	7.0	5.0	122
163	0	22.2	0	1	0	0	0	1	0	0	1	0	0	1	1	0	0	0	0	0	0	0	0	7.2	1	0	2	7.8	12	1.20	7.0	4.3	130
163	0	22.2	0	1	0	0	0	1	0	0	1	0	0	1	1	0	0	0	0	0	0	0	0	7.2	1	0	3	6.1	21	1.00	6.8	4.1	128
164	0	23.9	1	1	0	0	0	0	0	0	0	0	0	0	0	0	0	0	0	0	0	0	0	5.2	0	0	1	5.2	22	0.89	7.0	4.0	205
164	0	23.9	1	1	0	0	0	0	0	0	0	0	0	0	0	0	0	0	0	0	0	0	0	5.2	0	0	2	5.1	17	0.96	6.6	4.3	178
164	0	23.9	1	1	0	0	0	0	0	0	0	0	0	0	0	0	0	0	0	0	0	0	0	5.2	0	0	3	4.2	16	1.05	7.0	4.1	160
165	1	25.2	1	0	0	0	0	0	0	1	0	0	0	0	0	1	1	0	1	0	1	0	1	7.6	1	0	1	7.6	15	0.88	7.0	4.0	184
165	1	25.2	1	0	0	0	0	0	0	1	0	0	0	0	0	1	1	0	1	0	1	0	1	7.6	1	0	2	6.4	12	0.87	8.0	4.3	210
165	1	25.2	1	0	0	0	0	0	0	1	0	0	0	0	0	1	1	0	1	0	1	0	1	7.6	1	0	3	5.9	19	0.87	7.6	4.3	227
166	0	20.6	1	0	1	0	0	0	0	1	1	0	0	1	0	0	0	0	0	0	0	0	0	5.0	0	0	1	5.0	12	1.20	7.0	4.0	188
166	0	20.6	1	0	1	0	0	0	0	1	1	0	0	1	0	0	0	0	0	0	0	0	0	5.0	0	0	2	6.1	15	1.10	7.8	4.5	186
166	0	20.6	1	0	1	0	0	0	0	1	1	0	0	1	0	0	0	0	0	0	0	0	0	5.0	0	0	3	5.3	15	1.20	7.8	4.7	181
167	0	27.9	0	1	1	0	0	1	0	1	0	0	0	0	1	0	0	0	0	0	0	0	0	4.3	0	0	1	4.3	8	0.76	8.0	5.0	141
167	0	27.9	0	1	1	0	0	1	0	1	0	0	0	0	1	0	0	0	0	0	0	0	0	4.3	0	0	2	5.5	10	0.80	8.1	4.7	134

data_ADT_SUA

id	Group	BMI	Current_ETOH	Ever_smoke	HTN	DM	CAD	DYSLIPID	Stage_4	ECOG	Con_asa	Con_thia	con_loopD	con_ARB	con_statin	Anti_Androgen	Bicalutamide	Cyproterone	GNRH	Leuprorelin	Goserelin	Triptorelin	Conc_RT	Base_UA	Base_hyperUA	Base_hypoUA	time	UA	BUN	CR	PROT	ALB	CHOL
167	0	27.9	0	1	1	0	0	1	0	1	0	0	0	0	1	0	0	0	0	0	0	0	0	4.3	0	0	3	5.1	11	0.84	7.8	4.7	130
168	0	25.3	0	0	0	1	0	0	0	1	0	0	0	0	0	0	0	0	0	0	0	0	0	7.2	1	0	1	7.2	16	0.91	7.0	4.0	201
168	0	25.3	0	0	0	1	0	0	0	1	0	0	0	0	0	0	0	0	0	0	0	0	0	7.2	1	0	2	6.8	13	0.82	6.9	4.2	124
168	0	25.3	0	0	0	1	0	0	0	1	0	0	0	0	0	0	0	0	0	0	0	0	0	7.2	1	0	3	7.1	10	0.87	7.2	4.4	156
169	0	25.1	0	0	0	1	0	0	0	1	0	0	0	0	0	0	0	0	0	0	0	0	0	9.1	1	0	1	9.1	14	1.03	7.0	4.0	168
169	0	25.1	0	0	0	1	0	0	0	1	0	0	0	0	0	0	0	0	0	0	0	0	0	9.1	1	0	2	9.4	19	0.97	7.1	4.3	175
169	0	25.1	0	0	0	1	0	0	0	1	0	0	0	0	0	0	0	0	0	0	0	0	0	9.1	1	0	3	7.4	15	0.99	7.0	4.4	174
170	0	26.1	1	1	1	1	0	0	0	1	0	1	0	1	0	0	0	0	0	0	0	0	0	4.7	0	0	1	4.7	15	1.05	6.0	4.0	150
170	0	26.1	1	1	1	1	0	0	0	1	0	1	0	1	0	0	0	0	0	0	0	0	0	4.7	0	0	2	4.5	23	1.02	6.8	4.4	168
170	0	26.1	1	1	1	1	0	0	0	1	0	1	0	1	0	0	0	0	0	0	0	0	0	4.7	0	0	3	4.5	21	0.95	6.8	4.5	180
171	0	25.3	0	0	1	1	0	1	0	1	0	0	0	0	1	0	0	0	0	0	0	0	0	4.9	0	0	1	4.9	15	1.10	7.0	5.0	178
171	0	25.3	0	0	1	1	0	1	0	1	0	0	0	0	1	0	0	0	0	0	0	0	0	4.9	0	0	2	5.8	17	1.00	7.7	4.6	194
171	0	25.3	0	0	1	1	0	1	0	1	0	0	0	0	1	0	0	0	0	0	0	0	0	4.9	0	0	3	5.4	18	1.00	7.7	4.7	168
172	0	24.9	0	1	1	0	0	0	0	1	0	0	0	0	0	0	0	0	0	0	0	0	0	5.9	0	0	1	5.9	18	0.91	7.0	5.0	199
172	0	24.9	0	1	1	0	0	0	0	1	0	0	0	0	0	0	0	0	0	0	0	0	0	5.9	0	0	2	6.5	16	1.00	6.8	4.5	214
172	0	24.9	0	1	1	0	0	0	0	1	0	0	0	0	0	0	0	0	0	0	0	0	0	5.9	0	0	3	5.7	14	0.86	6.9	4.6	219
173	0	27.9	1	1	1	1	0	1	0	1	1	1	1	0	1	0	0	0	0	0	0	0	0	2.9	0	1	1	2.9	22	0.91	7.0	5.0	168
173	0	27.9	1	1	1	1	0	1	0	1	1	1	1	0	1	0	0	0	0	0	0	0	0	2.9	0	1	2	3.0	26	0.85	7.7	4.8	176
173	0	27.9	1	1	1	1	0	1	0	1	1	1	1	0	1	0	0	0	0	0	0	0	0	2.9	0	1	3	3.4	24	0.76	7.5	4.5	172
174	0	24.6	1	1	0	0	0	0	0	0	0	0	0	0	0	0	0	0	0	0	0	0	0	7.6	1	0	1	7.6	19	0.87	7.0	5.0	181
174	0	24.6	1	1	0	0	0	0	0	0	0	0	0	0	0	0	0	0	0	0	0	0	0	7.6	1	0	2	10.5	23	0.85	7.0	4.3	163
174	0	24.6	1	1	0	0	0	0	0	0	0	0	0	0	0	0	0	0	0	0	0	0	0	7.6	1	0	3	8.5	22	0.84	7.1	4.5	201
175	0	20.8	1	1	1	0	0	1	0	1	0	0	0	1	1	0	0	0	0	0	0	0	0	7.5	1	0	1	7.5	21	0.90	6.0	4.0	122
175	0	20.8	1	1	1	0	0	1	0	1	0	0	0	1	1	0	0	0	0	0	0	0	0	7.5	1	0	2	5.9	28	0.92	6.2	4.1	108
175	0	20.8	1	1	1	0	0	1	0	1	0	0	0	1	1	0	0	0	0	0	0	0	0	7.5	1	0	3	6.1	19	0.99	7.1	4.8	134
176	0	26.1	0	0	0	0	0	0	0	1	0	0	0	0	0	0	0	0	0	0	0	0	1	6.4	0	0	1	6.4	16	1.00	7.0	5.0	253
176	0	26.1	0	0	0	0	0	0	0	1	0	0	0	0	0	0	0	0	0	0	0	0	1	6.4	0	0	2	6.9	16	1.00	6.8	4.2	203
176	0	26.1	0	0	0	0	0	0	0	1	0	0	0	0	0	0	0	0	0	0	0	0	1	6.4	0	0	3	6.7	11	1.00	6.6	4.2	217
177	0	24.0	0	0	1	1	0	1	0	1	0	0	0	1	1	0	0	0	0	0	0	0	0	5.4	0	0	1	5.4	17	1.20	7.0	4.0	143
177	0	24.0	0	0	1	1	0	1	0	1	0	0	0	1	1	0	0	0	0	0	0	0	0	5.4	0	0	2	6.4	22	1.10	7.2	4.4	145
177	0	24.0	0	0	1	1	0	1	0	1	0	0	0	1	1	0	0	0	0	0	0	0	0	5.4	0	0	3	5.8	23	1.10	7.2	4.3	151
178	1	27.9	0	0	1	0	0	0	0	1	1	1	0	1	0	1	1	0	1	1	0	0	1	5.7	0	0	1	5.7	12	0.80	7.0	4.0	153
178	1	27.9	0	0	1	0	0	0	0	1	1	1	0	1	0	1	1	0	1	1	0	0	1	5.7	0	0	2	4.4	15	0.87	7.2	4.4	171
178	1	27.9	0	0	1	0	0	0	0	1	1	1	0	1	0	1	1	0	1	1	0	0	1	5.7	0	0	3	4.5	10	0.99	7.2	4.6	182
179	1	25.8	0	0	1	0	0	0	0	0	0	0	0	1	0	0	0	0	1	1	0	0	0	5.8	0	0	1	5.8	13	1.10	8.0	5.0	160
179	1	25.8	0	0	1	0	0	0	0	0	0	0	0	1	0	0	0	0	1	1	0	0	0	5.8	0	0	2	4.7	16	1.14	7.4	4.5	188
179	1	25.8	0	0	1	0	0	0	0	0	0	0	0	1	0	0	0	0	1	1	0	0	0	5.8	0	0	3	4.3	15	1.08	7.3	4.8	164
180	0	22.9	0	0	1	0	0	0	0	2	1	0	0	0	0	0	0	0	0	0	0	0	0	3.7	0	1	1	3.7	8	0.86	6.0	4.0	98
180	0	22.9	0	0	1	0	0	0	0	2	1	0	0	0	0	0	0	0	0	0	0	0	0	3.7	0	1	2	4.7	20	0.88	7.1	4.4	193
180	0	22.9	0	0	1	0	0	0	0	2	1	0	0	0	0	0	0	0	0	0	0	0	0	3.7	0	1	3	4.5	15	0.96	7.7	4.9	182
181	1	26.1	1	0	0	1	0	0	0	1	0	0	0	0	0	1	1	0	0	0	0	0	1	6.4	0	0	1	6.4	15	1.10	7.0	4.0	193
181	1	26.1	1	0	0	1	0	0	0	1	0	0	0	0	0	1	1	0	0	0	0	0	1	6.4	0	0	2	5.9	21	1.10	6.9	4.2	173
181	1	26.1	1	0	0	1	0	0	0	1	0	0	0	0	0	1	1	0	0	0	0	0	1	6.4	0	0	3	5.1	13	1.20	6.8	4.2	189
182	1	23.5	0	0	1	1	0	0	1	1	1	0	0	0	0	1	1	0	1	1	0	0	0	1.7	0	1	1	1.7	14	0.91	7.0	4.0	159
182	1	23.5	0	0	1	1	0	0	1	1	1	0	0	0	0	1	1	0	1	1	0	0	0	1.7	0	1	2	1.6	10	0.99	7.1	4.4	176
182	1	23.5	0	0	1	1	0	0	1	1	1	0	0	0	0	1	1	0	1	1	0	0	0	1.7	0	1	3	2.4	11	1.07	7.4	4.6	188
183	1	19.4	1	1	1	0	0	1	1	1	0	0	0	0	1	1	1	0	1	1	0	0	0	5.9	0	0	1	5.9	11	1.10	6.0	2.0	93
183	1	19.4	1	1	1	0	0	1	1	1	0	0	0	0	1	1	1	0	1	1	0	0	0	5.9	0	0	2	3.1	12	1.00	7.7	3.8	131
183	1	19.4	1	1	1	0	0	1	1	1	0	0	0	0	1	1	1	0	1	1	0	0	0	5.9	0	0	3	4.9	18	0.80	7.1	4.2	162
184	0	23.9	0	0	1	0	0	0	0	0	0	0	0	1	0	0	0	0	0	0	0	0	0	4.8	0	0	1	4.8	14	0.81	7.0	5.0	206

id	Group	BMI	Current_ETOH	Ever_smoke	HTN	DM	CAD	DYSLIPID	Stage_4	ECOG	Con_asa	Con_thia	con_loopD	con_ARB	con_statin	Anti_Androgen	Bicalutamide	Cyproterone	GNRH	Leuprorelin	Goserelin	Triptorelin	Conc_RT	Base_UA	Base_hyperUA	Base_hypoUA	time	UA	BUN	CR	PROT	ALB	CHOL
184	0	23.9	0	0	1	0	0	0	0	0	0	0	0	1	0	0	0	0	0	0	0	0	0	4.8	0	0	2	4.1	13	0.79	7.5	4.8	203
184	0	23.9	0	0	1	0	0	0	0	0	0	0	0	1	0	0	0	0	0	0	0	0	0	4.8	0	0	3	3.9	18	0.74	7.6	4.8	197
185	1	27.4	0	0	1	0	0	1	0	1	0	0	0	0	1	1	1	0	1	1	0	0	0	5.5	0	0	1	5.5	16	1.10	7.0	4.0	222
185	1	27.4	0	0	1	0	0	1	0	1	0	0	0	0	1	1	1	0	1	1	0	0	0	5.5	0	0	2	5.1	13	0.95	6.8	4.3	243
185	1	27.4	0	0	1	0	0	1	0	1	0	0	0	0	1	1	1	0	1	1	0	0	0	5.5	0	0	3	5.9	12	0.90	7.0	4.3	267
186	0	23.1	0	0	0	0	0	0	0	1	0	0	0	0	0	0	0	0	0	0	0	0	0	4.0	0	0	1	4.0	14	1.05	6.0	4.0	153
186	0	23.1	0	0	0	0	0	0	0	1	0	0	0	0	0	0	0	0	0	0	0	0	0	4.0	0	0	2	5.1	16	0.77	6.9	4.2	163
186	0	23.1	0	0	0	0	0	0	0	1	0	0	0	0	0	0	0	0	0	0	0	0	0	4.0	0	0	3	4.6	17	0.79	6.9	4.2	157
187	1	26.8	0	1	0	0	0	0	0	1	0	0	0	0	0	1	1	0	1	1	0	0	1	8.0	1	0	1	8.0	10	1.11	7.0	4.0	182
187	1	26.8	0	1	0	0	0	0	0	1	0	0	0	0	0	1	1	0	1	1	0	0	1	8.0	1	0	2	6.2	16	1.04	7.0	4.5	205
187	1	26.8	0	1	0	0	0	0	0	1	0	0	0	0	0	1	1	0	1	1	0	0	1	8.0	1	0	3	5.8	12	1.00	7.1	4.7	191
188	1	26.8	0	#NULL!	0	0	0	0	1	1	0	0	0	0	0	1	1	0	1	1	0	0	0	7.8	1	0	1	7.8	15	0.99	7.0	4.0	238
188	1	26.8	0	#NULL!	0	0	0	0	1	1	0	0	0	0	0	1	1	0	1	1	0	0	0	7.8	1	0	2	6.8	12	1.07	7.3	4.5	220
188	1	26.8	0	#NULL!	0	0	0	0	1	1	0	0	0	0	0	1	1	0	1	1	0	0	0	7.8	1	0	3	5.9	18	0.96	7.2	4.3	254
189	0	20.6	0	1	0	0	0	0	0	1	0	0	0	0	0	0	0	0	0	0	0	0	0	6.8	0	0	1	6.8	12	1.00	7.0	4.0	184
189	0	20.6	0	1	0	0	0	0	0	1	0	0	0	0	0	0	0	0	0	0	0	0	0	6.8	0	0	2	7.0	11	0.87	6.9	4.1	181
189	0	20.6	0	1	0	0	0	0	0	1	0	0	0	0	0	0	0	0	0	0	0	0	0	6.8	0	0	3	6.2	11	0.84	7.6	4.6	208
190	1	23.7	1	1	0	0	0	0	0	1	0	0	0	0	0	1	1	0	1	1	0	0	1	4.8	0	0	1	4.8	14	1.19	8.0	4.0	159
190	1	23.7	1	1	0	0	0	0	0	1	0	0	0	0	0	1	1	0	1	1	0	0	1	4.8	0	0	2	4.1	19	1.09	7.6	4.5	200
190	1	23.7	1	1	0	0	0	0	0	1	0	0	0	0	0	1	1	0	1	1	0	0	1	4.8	0	0	3	4.1	17	0.95	7.5	4.5	166
191	0	19.3	0	1	0	0	0	0	0	0	0	0	0	0	0	0	0	0	0	0	0	0	0	5.1	0	0	1	5.1	15	0.98	8.0	5.0	212
191	0	19.3	0	1	0	0	0	0	0	0	0	0	0	0	0	0	0	0	0	0	0	0	0	5.1	0	0	2	5.3	21	1.01	7.4	4.6	191
191	0	19.3	0	1	0	0	0	0	0	0	0	0	0	0	0	0	0	0	0	0	0	0	0	5.1	0	0	3	4.8	16	1.03	7.2	4.4	170
192	1	22.8	1	0	1	0	0	0	0	1	1	0	0	0	0	1	1	0	1	1	0	0	0	6.7	0	0	1	6.7	20	0.78	7.0	4.0	219
192	1	22.8	1	0	1	0	0	0	0	1	1	0	0	0	0	1	1	0	1	1	0	0	0	6.7	0	0	2	4.1	18	1.00	7.5	4.3	230
192	1	22.8	1	0	1	0	0	0	0	1	1	0	0	0	0	1	1	0	1	1	0	0	0	6.7	0	0	3	4.1	20	0.77	6.8	3.9	181
193	1	21.5	0	0	0	0	0	0	0	1	0	0	0	0	0	1	1	0	1	1	0	0	0	5.6	0	0	1	5.6	15	0.93	7.0	4.0	212
193	1	21.5	0	0	0	0	0	0	0	1	0	0	0	0	0	1	1	0	1	1	0	0	0	5.6	0	0	2	5.4	22	0.95	7.1	4.4	237
193	1	21.5	0	0	0	0	0	0	0	1	0	0	0	0	0	1	1	0	1	1	0	0	0	5.6	0	0	3	4.9	18	1.01	7.4	4.7	234
194	0	24.0	1	1	1	0	0	0	0	1	0	0	0	0	0	0	0	0	0	0	0	0	0	6.2	0	0	1	6.2	11	0.86	7.0	4.0	138
194	0	24.0	1	1	1	0	0	0	0	1	0	0	0	0	0	0	0	0	0	0	0	0	0	6.2	0	0	2	4.4	10	0.86	7.1	4.3	138
194	0	24.0	1	1	1	0	0	0	0	1	0	0	0	0	0	0	0	0	0	0	0	0	0	6.2	0	0	3	4.6	10	0.77	7.1	4.3	164
195	0	22.3	1	1	1	1	0	0	0	1	0	0	0	1	0	0	0	0	0	0	0	0	0	2.7	0	1	1	2.7	13	0.88	7.0	4.0	174
195	0	22.3	1	1	1	1	0	0	0	1	0	0	0	1	0	0	0	0	0	0	0	0	0	2.7	0	1	2	3.7	13	0.91	6.8	4.3	160
195	0	22.3	1	1	1	1	0	0	0	1	0	0	0	1	0	0	0	0	0	0	0	0	0	2.7	0	1	3	4.8	12	1.00	6.6	4.2	151
196	0	19.1	1	0	0	0	0	0	0	1	1	0	0	0	0	0	0	0	0	0	0	0	0	4.2	0	0	1	4.2	13	0.87	8.0	5.0	207
196	0	19.1	1	0	0	0	0	0	0	1	1	0	0	0	0	0	0	0	0	0	0	0	0	4.2	0	0	2	4.3	14	0.79	7.2	4.2	191
196	0	19.1	1	0	0	0	0	0	0	1	1	0	0	0	0	0	0	0	0	0	0	0	0	4.2	0	0	3	5.0	15	0.78	7.2	4.4	182
197	0	23.0	1	1	0	0	0	0	0	0	0	0	0	0	0	0	0	0	0	0	0	0	0	3.9	0	1	1	3.9	16	0.72	7.0	4.0	190
197	0	23.0	1	1	0	0	0	0	0	0	0	0	0	0	0	0	0	0	0	0	0	0	0	3.9	0	1	2	4.0	20	0.78	6.8	4.3	189
197	0	23.0	1	1	0	0	0	0	0	0	0	0	0	0	0	0	0	0	0	0	0	0	0	3.9	0	1	3	4.4	18	0.92	6.3	3.8	185
198	0	24.2	1	0	1	0	0	1	0	1	0	1	0	0	1	0	0	0	0	0	0	0	0	5.5	0	0	1	5.5	16	0.90	8.0	5.0	209
198	0	24.2	1	0	1	0	0	1	0	1	0	1	0	0	1	0	0	0	0	0	0	0	0	5.5	0	0	2	5.7	19	1.02	7.5	4.5	205
198	0	24.2	1	0	1	0	0	1	0	1	0	1	0	0	1	0	0	0	0	0	0	0	0	5.5	0	0	3	4.8	17	1.03	7.2	4.4	192
199	1	25.1	1	1	0	0	0	0	0	1	0	0	0	0	0	0	0	0	1	0	1	0	0	5.3	0	0	1	5.3	15	0.99	6.0	4.0	172
199	1	25.1	1	1	0	0	0	0	0	1	0	0	0	0	0	0	0	0	1	0	1	0	0	5.3	0	0	2	4.1	15	0.95	6.5	3.8	165
199	1	25.1	1	1	0	0	0	0	0	1	0	0	0	0	0	0	0	0	1	0	1	0	0	5.3	0	0	3	4.6	14	0.94	7.2	4.2	137
200	0	21.5	1	0	0	0	0	0	0	0	0	0	0	0	0	0	0	0	0	0	0	0	0	7.4	1	0	1	7.4	17	1.09	7.0	4.0	192
200	0	21.5	1	0	0	0	0	0	0	0	0	0	0	0	0	0	0	0	0	0	0	0	0	7.4	1	0	2	8.1	18	1.12	7.0	4.4	174
200	0	21.5	1	0	0	0	0	0	0	0	0	0	0	0	0	0	0	0	0	0	0	0	0	7.4	1	0	3	6.7	15	1.18	6.9	3.9	143

id	Group	BMI	Current_ETOH	Ever_smoke	HTN	DM	CAD	DYSLIPID	Stage_4	ECOG	Con_asa	Con_thia	con_loopD	con_ARB	con_statin	Anti_Androgen	Bicalutamide	Cyproterone	GNRH	Leuprorelin	Goserelin	Triptorelin	Conc_RT	Base_UA	Base_hyperUA	Base_hypoUA	time	UA	BUN	CR	PROT	ALB	CHOL
201	0	27.5	0	0	1	0	0	0	0	1	0	1	0	1	0	0	0	0	0	0	0	0	0	7.6	1	0	1	7.6	18	1.00	7.0	4.0	192
201	0	27.5	0	0	1	0	0	0	0	1	0	1	0	1	0	0	0	0	0	0	0	0	0	7.6	1	0	2	7.4	17	1.00	7.1	4.3	174
201	0	27.5	0	0	1	0	0	0	0	1	0	1	0	1	0	0	0	0	0	0	0	0	0	7.6	1	0	3	6.8	16	0.81	7.3	4.4	181
202	0	22.9	0	1	0	1	0	1	0	1	0	0	0	0	1	0	0	0	0	0	0	0	0	6.5	0	0	1	6.5	10	0.71	7.0	4.0	145
202	0	22.9	0	1	0	1	0	1	0	1	0	0	0	0	1	0	0	0	0	0	0	0	0	6.5	0	0	2	4.8	11	0.61	6.7	4.1	165
202	0	22.9	0	1	0	1	0	1	0	1	0	0	0	0	1	0	0	0	0	0	0	0	0	6.5	0	0	3	5.7	10	0.59	7.0	4.4	178
203	1	29.6	0	0	1	0	0	1	1	1	0	0	0	0	1	1	1	0	0	0	0	0	1	6.4	0	0	1	6.4	17	0.94	8.0	5.0	148
203	1	29.6	0	0	1	0	0	1	1	1	0	0	0	0	1	1	1	0	0	0	0	0	1	6.4	0	0	2	5.6	13	0.92	6.9	4.2	198
203	1	29.6	0	0	1	0	0	1	1	1	0	0	0	0	1	1	1	0	0	0	0	0	1	6.4	0	0	3	4.7	15	0.93	7.5	4.6	151
204	0	23.1	1	0	1	0	0	0	0	1	1	0	0	0	0	0	0	0	0	0	0	0	0	5.2	0	0	1	5.2	12	1.00	7.0	5.0	202
204	0	23.1	1	0	1	0	0	0	0	1	1	0	0	0	0	0	0	0	0	0	0	0	0	5.2	0	0	2	4.9	15	1.00	6.9	4.5	178
204	0	23.1	1	0	1	0	0	0	0	1	1	0	0	0	0	0	0	0	0	0	0	0	0	5.2	0	0	3	5.5	13	1.00	6.8	4.4	180
205	0	21.3	1	1	0	0	0	0	0	0	0	0	0	0	0	0	0	0	0	0	0	0	0	6.2	0	0	1	6.2	20	0.97	7.0	4.0	164
205	0	21.3	1	1	0	0	0	0	0	0	0	0	0	0	0	0	0	0	0	0	0	0	0	6.2	0	0	2	5.6	17	0.93	7.1	4.4	147
205	0	21.3	1	1	0	0	0	0	0	0	0	0	0	0	0	0	0	0	0	0	0	0	0	6.2	0	0	3	6.0	12	0.95	5.4	3.0	117
206	0	35.9	1	1	1	0	0	0	0	2	0	1	0	1	0	0	0	0	0	0	0	0	0	3.4	0	1	1	3.4	14	0.77	7.0	4.0	172
206	0	35.9	1	1	1	0	0	0	0	2	0	1	0	1	0	0	0	0	0	0	0	0	0	3.4	0	1	2	3.6	16	0.80	6.9	4.6	163
206	0	35.9	1	1	1	0	0	0	0	2	0	1	0	1	0	0	0	0	0	0	0	0	0	3.4	0	1	3	4.3	15	0.86	6.9	4.4	192
207	1	24.1	0	1	0	0	0	0	1	1	0	0	0	0	0	1	1	0	1	0	1	0	0	4.6	0	0	1	4.6	18	0.92	8.0	5.0	211
207	1	24.1	0	1	0	0	0	0	1	1	0	0	0	0	0	1	1	0	1	0	1	0	0	4.6	0	0	2	3.6	18	0.89	7.5	4.5	213
207	1	24.1	0	1	0	0	0	0	1	1	0	0	0	0	0	1	1	0	1	0	1	0	0	4.6	0	0	3	3.6	15	0.86	7.6	4.5	211
208	1	25.1	0	0	0	0	0	0	0	1	0	0	0	0	0	1	1	0	1	1	0	0	0	7.7	1	0	1	7.7	15	1.10	6.0	4.0	179
208	1	25.1	0	0	0	0	0	0	0	1	0	0	0	0	0	1	1	0	1	1	0	0	0	7.7	1	0	2	6.3	14	1.10	7.1	4.2	215
208	1	25.1	0	0	0	0	0	0	0	1	0	0	0	0	0	1	1	0	1	1	0	0	0	7.7	1	0	3	5.3	15	1.10	6.7	4.0	202
209	1	27.1	1	1	1	0	0	0	0	1	0	0	0	1	0	1	1	0	1	1	0	0	1	6.1	0	0	1	6.1	19	0.97	7.0	4.0	170
209	1	27.1	1	1	1	0	0	0	0	1	0	0	0	1	0	1	1	0	1	1	0	0	1	6.1	0	0	2	5.9	17	1.04	6.9	4.4	187
209	1	27.1	1	1	1	0	0	0	0	1	0	0	0	1	0	1	1	0	1	1	0	0	1	6.1	0	0	3	4.7	22	1.10	6.6	4.1	163
210	0	26.1	0	1	1	1	0	0	0	2	1	0	0	1	1	0	0	0	0	0	0	0	0	8.9	1	0	1	8.9	19	0.94	6.0	4.0	191
210	0	26.1	0	1	1	1	0	0	0	2	1	0	0	1	1	0	0	0	0	0	0	0	0	8.9	1	0	2	10.1	22	1.03	6.8	4.2	244
210	0	26.1	0	1	1	1	0	0	0	2	1	0	0	1	1	0	0	0	0	0	0	0	0	8.9	1	0	3	8.7	23	0.92	6.7	4.3	221
211	0	23.2	0	1	0	0	0	0	0	2	0	0	0	0	0	0	0	0	0	0	0	0	0	6.3	0	0	1	6.3	22	1.02	8.0	4.0	175
211	0	23.2	0	1	0	0	0	0	0	2	0	0	0	0	0	0	0	0	0	0	0	0	0	6.3	0	0	2	7.8	20	1.00	8.0	4.3	172
211	0	23.2	0	1	0	0	0	0	0	2	0	0	0	0	0	0	0	0	0	0	0	0	0	6.3	0	0	3	7.2	16	0.90	7.8	4.3	150
212	0	25.7	1	1	0	0	0	0	0	0	0	0	0	0	0	0	0	0	0	0	0	0	0	5.1	0	0	1	5.1	16	1.19	8.0	5.0	148
212	0	25.7	1	1	0	0	0	0	0	0	0	0	0	0	0	0	0	0	0	0	0	0	0	5.1	0	0	2	5.5	17	0.98	7.9	4.5	146
212	0	25.7	1	1	0	0	0	0	0	0	0	0	0	0	0	0	0	0	0	0	0	0	0	5.1	0	0	3	5.9	23	1.00	7.8	4.4	128
213	1	26.2	0	1	1	0	0	0	0	1	1	1	0	1	0	1	1	0	1	1	0	0	1	6.4	0	0	1	6.4	14	1.13	7.0	4.0	196
213	1	26.2	0	1	1	0	0	0	0	1	1	1	0	1	0	1	1	0	1	1	0	0	1	6.4	0	0	2	5.1	17	1.09	6.7	4.2	203
213	1	26.2	0	1	1	0	0	0	0	1	1	1	0	1	0	1	1	0	1	1	0	0	1	6.4	0	0	3	5.2	12	1.02	6.7	4.0	213
214	1	22.7	0	0	0	0	0	0	0	1	0	0	0	0	0	1	1	0	1	0	1	0	0	3.8	0	1	1	3.8	12	1.03	6.0	4.0	183
214	1	22.7	0	0	0	0	0	0	0	1	0	0	0	0	0	1	1	0	1	0	1	0	0	3.8	0	1	2	4.3	18	1.02	6.8	4.6	226
214	1	22.7	0	0	0	0	0	0	0	1	0	0	0	0	0	1	1	0	1	0	1	0	0	3.8	0	1	3	4.2	17	0.92	6.8	4.5	217
215	1	29.8	0	0	1	1	0	0	0	2	0	0	0	1	0	1	1	0	1	0	1	0	0	5.6	0	0	1	5.6	16	1.20	7.0	4.0	213
215	1	29.8	0	0	1	1	0	0	0	2	0	0	0	1	0	1	1	0	1	0	1	0	0	5.6	0	0	2	5.2	18	1.20	7.5	4.6	218
215	1	29.8	0	0	1	1	0	0	0	2	0	0	0	1	0	1	1	0	1	0	1	0	0	5.6	0	0	3	5.9	16	1.10	7.3	4.5	211
216	0	20.2	0	0	1	0	0	0	0	2	1	0	0	0	0	0	0	0	0	0	0	0	0	4.9	0	0	1	4.9	17	0.95	7.0	4.0	182
216	0	20.2	0	0	1	0	0	0	0	2	1	0	0	0	0	0	0	0	0	0	0	0	0	4.9	0	0	2	4.7	14	0.83	7.6	4.6	198
216	0	20.2	0	0	1	0	0	0	0	2	1	0	0	0	0	0	0	0	0	0	0	0	0	4.9	0	0	3	4.3	17	0.83	7.4	4.5	183
217	1	19.5	1	0	0	0	0	0	1	3	0	0	0	0	0	1	1	0	1	0	1	0	1	6.2	0	0	1	6.2	23	1.00	7.0	4.0	137
217	1	19.5	1	0	0	0	0	0	1	3	0	0	0	0	0	1	1	0	1	0	1	0	1	6.2	0	0	2	5.2	22	1.00	6.6	4.2	115

id	Group	BMI	Current_ETOH	Ever_smoke	HTN	DM	CAD	DYSLIPID	Stage_4	ECOG	Con_asa	Con_thia	con_loopD	con_ARB	con_statin	Anti_Androgen	Bicalutamide	Cyproterone	GNRH	Leuprorelin	Goserelin	Triptorelin	Conc_RT	Base_UA	Base_hyperUA	Base_hypoUA	time	UA	BUN	CR	PROT	ALB	CHOL
217	1	19.5	1	0	0	0	0	0	1	3	0	0	0	0	0	1	1	0	1	0	1	0	1	6.2	0	0	3	4.1	19	0.90	5.9	3.2	83
218	1	25.1	1	1	1	0	0	0	0	1	0	1	0	1	0	1	1	0	1	0	1	0	1	5.4	0	0	1	5.4	16	1.10	7.0	4.0	156
218	1	25.1	1	1	1	0	0	0	0	1	0	1	0	1	0	1	1	0	1	0	1	0	1	5.4	0	0	2	5.5	14	1.10	7.0	4.3	211
218	1	25.1	1	1	1	0	0	0	0	1	0	1	0	1	0	1	1	0	1	0	1	0	1	5.4	0	0	3	5.7	11	1.10	7.3	4.6	221
219	0	27.5	0	0	1	1	0	0	0	2	0	0	0	1	1	0	0	0	0	0	0	0	0	3.0	0	1	1	3.0	26	1.00	7.0	5.0	160
219	0	27.5	0	0	1	1	0	0	0	2	0	0	0	1	1	0	0	0	0	0	0	0	0	3.0	0	1	2	2.5	16	1.00	6.8	4.6	138
219	0	27.5	0	0	1	1	0	0	0	2	0	0	0	1	1	0	0	0	0	0	0	0	0	3.0	0	1	3	3.0	21	1.10	7.1	4.7	139
220	0	25.1	0	0	0	0	0	0	0	2	0	0	0	0	0	0	0	0	0	0	0	0	0	7.8	1	0	1	7.8	17	1.10	7.0	4.0	206
220	0	25.1	0	0	0	0	0	0	0	2	0	0	0	0	0	0	0	0	0	0	0	0	0	7.8	1	0	2	7.1	15	1.10	8.1	4.4	222
220	0	25.1	0	0	0	0	0	0	0	2	0	0	0	0	0	0	0	0	0	0	0	0	0	7.8	1	0	3	6.6	19	1.00	7.8	4.3	215
221	0	22.9	0	1	0	0	0	0	0	1	0	0	0	0	0	0	0	0	0	0	0	0	0	5.7	0	0	1	5.7	20	0.93	7.0	4.0	195
221	0	22.9	0	1	0	0	0	0	0	1	0	0	0	0	0	0	0	0	0	0	0	0	0	5.7	0	0	2	6.3	22	0.86	7.4	4.1	229
221	0	22.9	0	1	0	0	0	0	0	1	0	0	0	0	0	0	0	0	0	0	0	0	0	5.7	0	0	3	6.2	25	0.92	6.9	4.1	225
222	1	25.0	0	0	1	1	0	0	0	1	0	0	0	0	0	0	0	0	1	0	1	0	0	8.6	1	0	1	8.6	13	0.70	6.0	4.0	132
222	1	25.0	0	0	1	1	0	0	0	1	0	0	0	0	0	0	0	0	1	0	1	0	0	8.6	1	0	2	8.9	22	0.77	7.4	4.8	200
222	1	25.0	0	0	1	1	0	0	0	1	0	0	0	0	0	0	0	0	1	0	1	0	0	8.6	1	0	3	9.5	19	0.61	6.8	4.6	151
223	0	27.9	0	1	0	0	0	0	0	0	0	0	0	0	0	0	0	0	0	0	0	0	0	6.4	0	0	1	6.4	19	0.80	7.0	4.0	194
223	0	27.9	0	1	0	0	0	0	0	0	0	0	0	0	0	0	0	0	0	0	0	0	0	6.4	0	0	2	6.3	21	0.90	7.0	4.7	205
223	0	27.9	0	1	0	0	0	0	0	0	0	0	0	0	0	0	0	0	0	0	0	0	0	6.4	0	0	3	7.9	10	1.00	6.9	4.4	182
224	0	28.3	0	0	1	0	0	1	0	2	0	1	0	1	1	0	0	0	0	0	0	0	0	6.8	0	0	1	6.8	11	1.10	7.0	4.0	158
224	0	28.3	0	0	1	0	0	1	0	2	0	1	0	1	1	0	0	0	0	0	0	0	0	6.8	0	0	2	7.3	13	1.10	6.8	4.3	166
224	0	28.3	0	0	1	0	0	1	0	2	0	1	0	1	1	0	0	0	0	0	0	0	0	6.8	0	0	3	7.1	12	1.00	7.0	4.3	174
225	1	24.7	0	0	1	0	0	0	0	1	0	0	0	1	1	1	1	0	0	0	0	0	1	5.0	0	0	1	5.0	11	0.97	7.0	4.0	246
225	1	24.7	0	0	1	0	0	0	0	1	0	0	0	1	1	1	1	0	0	0	0	0	1	5.0	0	0	2	4.8	15	1.01	7.4	4.5	199
225	1	24.7	0	0	1	0	0	0	0	1	0	0	0	1	1	1	1	0	0	0	0	0	1	5.0	0	0	3	4.7	20	0.95	7.1	4.2	207
226	0	25.1	1	1	1	0	0	0	0	0	1	1	0	1	0	0	0	0	0	0	0	0	0	8.2	1	0	1	8.2	12	1.20	8.0	5.0	220
226	0	25.1	1	1	1	0	0	0	0	0	1	1	0	1	0	0	0	0	0	0	0	0	0	8.2	1	0	2	8.4	21	1.00	7.3	4.6	137
226	0	25.1	1	1	1	0	0	0	0	0	1	1	0	1	0	0	0	0	0	0	0	0	0	8.2	1	0	3	8.1	16	1.10	6.9	4.3	192
227	0	25.0	0	1	0	0	0	0	0	1	0	0	0	0	1	0	0	0	0	0	0	0	0	4.7	0	0	1	4.7	13	1.10	7.0	5.0	245
227	0	25.0	0	1	0	0	0	0	0	1	0	0	0	0	1	0	0	0	0	0	0	0	0	4.7	0	0	2	4.3	10	1.10	6.9	4.2	231
227	0	25.0	0	1	0	0	0	0	0	1	0	0	0	0	1	0	0	0	0	0	0	0	0	4.7	0	0	3	4.3	11	1.20	7.3	4.6	160
228	1	22.0	0	0	0	0	0	0	1	0	0	0	0	0	0	1	1	0	1	0	1	0	0	4.8	0	0	1	4.8	13	0.97	8.0	5.0	169
228	1	22.0	0	0	0	0	0	0	1	0	0	0	0	0	0	1	1	0	1	0	1	0	0	4.8	0	0	2	4.0	19	0.96	7.6	4.5	185
228	1	22.0	0	0	0	0	0	0	1	0	0	0	0	0	0	1	1	0	1	0	1	0	0	4.8	0	0	3	4.3	16	0.94	7.5	4.6	204
229	1	21.8	0	0	0	0	0	0	0	1	0	0	0	0	0	1	1	0	0	0	0	0	1	6.3	0	0	1	6.3	15	0.87	8.0	5.0	176
229	1	21.8	0	0	0	0	0	0	0	1	0	0	0	0	0	1	1	0	0	0	0	0	1	6.3	0	0	2	6.4	14	0.85	7.2	4.6	204
229	1	21.8	0	0	0	0	0	0	0	1	0	0	0	0	0	1	1	0	0	0	0	0	1	6.3	0	0	3	5.0	14	0.82	7.5	4.7	192
230	0	23.8	1	0	1	1	0	0	0	1	1	0	0	0	0	0	0	0	0	0	0	0	0	6.0	0	0	1	6.0	16	1.10	7.0	4.0	196
230	0	23.8	1	0	1	1	0	0	0	1	1	0	0	0	0	0	0	0	0	0	0	0	0	6.0	0	0	2	5.0	16	1.00	7.3	4.5	211
230	0	23.8	1	0	1	1	0	0	0	1	1	0	0	0	0	0	0	0	0	0	0	0	0	6.0	0	0	3	6.8	20	1.00	7.0	4.3	207
231	1	24.4	1	1	0	0	0	1	0	1	0	0	0	0	1	1	1	0	1	0	1	0	1	5.5	0	0	1	5.5	17	1.10	7.0	4.0	213
231	1	24.4	1	1	0	0	0	1	0	1	0	0	0	0	1	1	1	0	1	0	1	0	1	5.5	0	0	2	5.0	22	1.10	6.6	4.0	239
231	1	24.4	1	1	0	0	0	1	0	1	0	0	0	0	1	1	1	0	1	0	1	0	1	5.5	0	0	3	4.5	21	1.10	6.8	4.5	161
232	1	29.4	1	0	0	1	0	1	1	2	0	0	0	0	1	1	1	0	1	0	1	0	0	4.8	0	0	1	4.8	9	1.10	7.0	4.0	140
232	1	29.4	1	0	0	1	0	1	1	2	0	0	0	0	1	1	1	0	1	0	1	0	0	4.8	0	0	2	4.8	10	1.10	7.1	4.3	150
232	1	29.4	1	0	0	1	0	1	1	2	0	0	0	0	1	1	1	0	1	0	1	0	0	4.8	0	0	3	3.8	12	1.00	7.1	4.4	154
233	0	26.4	0	0	0	0	0	0	0	1	0	0	0	0	0	0	0	0	0	0	0	0	0	5.6	0	0	1	5.6	17	1.10	7.0	4.0	170
233	0	26.4	0	0	0	0	0	0	0	1	0	0	0	0	0	0	0	0	0	0	0	0	0	5.6	0	0	2	4.9	18	1.20	7.3	4.4	200
233	0	26.4	0	0	0	0	0	0	0	1	0	0	0	0	0	0	0	0	0	0	0	0	0	5.6	0	0	3	4.4	21	1.20	6.8	4.2	193
234	1	26.1	1	1	1	0	0	1	0	1	0	0	0	1	1	1	1	0	1	1	0	0	1	5.0	0	0	1	5.0	18	0.76	8.0	4.0	142

data_ADT_SUA

id	Group	BMI	Current_ETOH	Ever_smoke	HTN	DM	CAD	DYSLIPID	Stage_4	ECOG	Con_asa	Con_thia	con_loopD	con_ARB	con_statin	Anti_Androgen	Bicalutamide	Cyproterone	GNRH	Leuprorelin	Goserelin	Triptorelin	Conc_RT	Base_UA	Base_hyperUA	Base_hypoUA	time	UA	BUN	CR	PROT	ALB	CHOL
234	1	26.1	1	1	1	0	0	1	0	1	0	0	0	1	1	1	1	0	1	1	0	0	1	5.0	0	0	2	4.7	17	0.72	7.8	4.7	148
234	1	26.1	1	1	1	0	0	1	0	1	0	0	0	1	1	1	1	0	1	1	0	0	1	5.0	0	0	3	4.6	17	0.61	7.5	4.5	158
235	0	27.1	1	1	0	0	0	0	0	2	0	0	0	0	0	0	0	0	0	0	0	0	0	6.8	0	0	1	6.8	19	0.60	7.0	4.0	207
235	0	27.1	1	1	0	0	0	0	0	2	0	0	0	0	0	0	0	0	0	0	0	0	0	6.8	0	0	2	6.8	13	0.68	7.0	4.3	192
235	0	27.1	1	1	0	0	0	0	0	2	0	0	0	0	0	0	0	0	0	0	0	0	0	6.8	0	0	3	7.5	20	0.73	7.0	4.5	188
236	1	23.5	1	1	0	0	0	0	1	1	0	0	0	0	0	1	1	0	1	0	1	0	1	7.6	1	0	1	7.6	15	0.86	7.0	4.0	230
236	1	23.5	1	1	0	0	0	0	1	1	0	0	0	0	0	1	1	0	1	0	1	0	1	7.6	1	0	2	6.4	19	0.91	6.8	4.5	245
236	1	23.5	1	1	0	0	0	0	1	1	0	0	0	0	0	1	1	0	1	0	1	0	1	7.6	1	0	3	6.0	17	0.83	7.1	4.7	257
237	0	23.0	0	1	1	0	0	0	0	1	1	0	0	0	0	0	0	0	0	0	0	0	0	6.1	0	0	1	6.1	18	0.72	7.0	4.0	181
237	0	23.0	0	1	1	0	0	0	0	1	1	0	0	0	0	0	0	0	0	0	0	0	0	6.1	0	0	2	5.9	19	0.76	7.1	4.5	222
237	0	23.0	0	1	1	0	0	0	0	1	1	0	0	0	0	0	0	0	0	0	0	0	0	6.1	0	0	3	6.0	19	0.78	6.9	4.4	204
238	0	21.5	0	1	0	0	0	0	0	0	0	0	0	0	0	0	0	0	0	0	0	0	0	7.0	1	0	1	7.0	13	1.17	7.0	5.0	210
238	0	21.5	0	1	0	0	0	0	0	0	0	0	0	0	0	0	0	0	0	0	0	0	0	7.0	1	0	2	6.1	16	1.13	6.7	4.3	221
238	0	21.5	0	1	0	0	0	0	0	0	0	0	0	0	0	0	0	0	0	0	0	0	0	7.0	1	0	3	5.9	17	1.13	7.1	4.6	195
239	0	24.3	0	1	0	0	0	0	0	0	0	0	0	0	0	0	0	0	0	0	0	0	0	5.3	0	0	1	5.3	9	0.86	8.0	5.0	184
239	0	24.3	0	1	0	0	0	0	0	0	0	0	0	0	0	0	0	0	0	0	0	0	0	5.3	0	0	2	6.2	13	0.98	7.4	4.0	170
239	0	24.3	0	1	0	0	0	0	0	0	0	0	0	0	0	0	0	0	0	0	0	0	0	5.3	0	0	3	5.4	10	0.81	7.5	4.4	178
240	0	23.9	1	0	0	1	0	1	0	2	0	0	0	0	1	0	0	0	0	0	0	0	0	5.5	0	0	1	5.5	22	1.00	7.0	4.0	192
240	0	23.9	1	0	0	1	0	1	0	2	0	0	0	0	1	0	0	0	0	0	0	0	0	5.5	0	0	2	5.3	16	0.98	7.4	4.6	187
240	0	23.9	1	0	0	1	0	1	0	2	0	0	0	0	1	0	0	0	0	0	0	0	0	5.5	0	0	3	5.3	16	0.92	7.1	4.2	186
241	0	23.4	0	0	0	0	0	0	0	0	0	0	0	0	0	0	0	0	0	0	0	0	0	3.3	0	1	1	3.3	15	1.20	8.0	4.0	137
241	0	23.4	0	0	0	0	0	0	0	0	0	0	0	0	0	0	0	0	0	0	0	0	0	3.3	0	1	2	4.3	17	1.20	8.3	4.0	143
241	0	23.4	0	0	0	0	0	0	0	0	0	0	0	0	0	0	0	0	0	0	0	0	0	3.3	0	1	3	3.6	12	0.80	8.2	4.2	124
242	0	24.3	1	0	1	0	0	0	0	1	0	0	0	1	0	0	0	0	0	0	0	0	0	5.8	0	0	1	5.8	20	0.97	8.0	5.0	157
242	0	24.3	1	0	1	0	0	0	0	1	0	0	0	1	0	0	0	0	0	0	0	0	0	5.8	0	0	2	5.6	18	0.99	7.2	1.4	133
242	0	24.3	1	0	1	0	0	0	0	1	0	0	0	1	0	0	0	0	0	0	0	0	0	5.8	0	0	3	5.5	20	0.96	6.8	4.2	171
243	0	23.1	1	1	0	0	0	0	0	1	0	0	0	0	0	0	0	0	0	0	0	0	0	4.4	0	0	1	4.4	20	1.19	7.0	4.0	233
243	0	23.1	1	1	0	0	0	0	0	1	0	0	0	0	0	0	0	0	0	0	0	0	0	4.4	0	0	2	6.1	13	1.03	6.5	3.9	194
243	0	23.1	1	1	0	0	0	0	0	1	0	0	0	0	0	0	0	0	0	0	0	0	0	4.4	0	0	3	5.6	19	1.03	6.5	3.9	189
244	1	24.9	0	0	0	0	0	0	0	0	0	0	0	0	0	1	1	0	0	0	0	0	1	6.5	0	0	1	6.5	19	1.10	7.0	4.0	207
244	1	24.9	0	0	0	0	0	0	0	0	0	0	0	0	0	1	1	0	0	0	0	0	1	6.5	0	0	2	5.3	15	1.10	7.3	4.5	246
244	1	24.9	0	0	0	0	0	0	0	0	0	0	0	0	0	1	1	0	0	0	0	0	1	6.5	0	0	3	5.4	14	0.90	7.2	4.5	222
245	0	21.8	1	1	1	0	0	0	0	1	0	0	0	1	0	0	0	0	0	0	0	0	0	5.2	0	0	1	5.2	16	1.10	7.0	4.0	172
245	0	21.8	1	1	1	0	0	0	0	1	0	0	0	1	0	0	0	0	0	0	0	0	0	5.2	0	0	2	5.2	14	1.10	7.0	4.2	198
245	0	21.8	1	1	1	0	0	0	0	1	0	0	0	1	0	0	0	0	0	0	0	0	0	5.2	0	0	3	6.3	18	0.99	7.0	4.2	186
246	1	25.6	1	0	0	1	0	1	0	1	0	0	0	0	1	1	1	0	0	0	0	0	1	5.2	0	0	1	5.2	13	1.10	8.0	4.0	231
246	1	25.6	1	0	0	1	0	1	0	1	0	0	0	0	1	1	1	0	0	0	0	0	1	5.2	0	0	2	4.0	16	1.10	7.1	4.1	234
246	1	25.6	1	0	0	1	0	1	0	1	0	0	0	0	1	1	1	0	0	0	0	0	1	5.2	0	0	3	4.0	20	0.90	7.2	4.2	158
247	0	23.0	1	0	0	0	0	0	0	0	0	0	0	0	0	0	0	0	0	0	0	0	0	5.0	0	0	1	5.0	17	1.16	7.0	4.0	201
247	0	23.0	1	0	0	0	0	0	0	0	0	0	0	0	0	0	0	0	0	0	0	0	0	5.0	0	0	2	4.8	13	1.14	7.1	4.4	196
247	0	23.0	1	0	0	0	0	0	0	0	0	0	0	0	0	0	0	0	0	0	0	0	0	5.0	0	0	3	4.9	18	1.03	7.4	4.6	208
248	1	19.6	0	0	0	0	0	0	1	1	0	0	0	0	0	1	1	0	1	0	1	0	1	4.8	0	0	1	4.8	17	1.10	7.0	4.0	153
248	1	19.6	0	0	0	0	0	0	1	1	0	0	0	0	0	1	1	0	1	0	1	0	1	4.8	0	0	2	4.5	15	1.10	6.9	4.0	157
248	1	19.6	0	0	0	0	0	0	1	1	0	0	0	0	0	1	1	0	1	0	1	0	1	4.8	0	0	3	4.6	13	1.20	7.1	4.3	171
249	1	27.1	1	0	0	1	0	0	0	1	0	0	0	0	0	1	1	0	1	0	1	0	0	4.2	0	0	1	4.2	11	0.89	7.0	4.0	147
249	1	27.1	1	0	0	1	0	0	0	1	0	0	0	0	0	1	1	0	1	0	1	0	0	4.2	0	0	2	4.2	15	0.76	6.5	4.6	152
249	1	27.1	1	0	0	1	0	0	0	1	0	0	0	0	0	1	1	0	1	0	1	0	0	4.2	0	0	3	3.3	12	0.77	6.9	4.7	165
250	0	21.7	0	0	0	1	0	0	0	2	0	0	0	0	0	0	0	0	0	0	0	0	0	6.2	0	0	1	6.2	25	0.94	6.0	4.0	169
250	0	21.7	0	0	0	1	0	0	0	2	0	0	0	0	0	0	0	0	0	0	0	0	0	6.2	0	0	2	6.4	18	1.03	6.9	4.3	183
250	0	21.7	0	0	0	1	0	0	0	2	0	0	0	0	0	0	0	0	0	0	0	0	0	6.2	0	0	3	7.1	16	0.91	6.7	4.2	177

data_ADT_SUA

id	Group	BMI	Current_ETOH	Ever_smoke	HTN	DM	CAD	DYSLIPID	Stage_4	ECOG	Con_asa	Con_thia	con_loopD	con_ARB	con_statin	Anti_Androgen	Bicalutamide	Cyproterone	GNRH	Leuprorelin	Goserelin	Triptorelin	Conc_RT	Base_UA	Base_hyperUA	Base_hypoUA	time	UA	BUN	CR	PROT	ALB	CHOL
251	1	26.0	0	0	1	1	0	0	1	1	0	0	0	0	0	1	1	0	1	0	1	0	0	5.1	0	0	1	5.1	21	1.09	6.0	3.0	181
251	1	26.0	0	0	1	1	0	0	1	1	0	0	0	0	0	1	1	0	1	0	1	0	0	5.1	0	0	2	5.1	21	1.11	6.5	4.0	232
251	1	26.0	0	0	1	1	0	0	1	1	0	0	0	0	0	1	1	0	1	0	1	0	0	5.1	0	0	3	4.9	15	1.07	7.1	4.3	282
252	1	23.1	0	0	0	0	0	0	0	2	0	0	0	0	0	1	1	0	1	1	0	0	0	4.1	0	0	1	4.1	14	1.11	7.0	4.0	211
252	1	23.1	0	0	0	0	0	0	0	2	0	0	0	0	0	1	1	0	1	1	0	0	0	4.1	0	0	2	3.6	17	0.93	6.9	4.3	205
252	1	23.1	0	0	0	0	0	0	0	2	0	0	0	0	0	1	1	0	1	1	0	0	0	4.1	0	0	3	4.2	14	1.04	7.2	4.7	259
253	0	23.4	0	0	0	0	0	0	0	1	1	0	0	0	0	0	0	0	0	0	0	0	0	3.8	0	1	1	3.8	15	0.90	7.0	4.0	184
253	0	23.4	0	0	0	0	0	0	0	1	1	0	0	0	0	0	0	0	0	0	0	0	0	3.8	0	1	2	6.2	17	1.00	7.0	4.4	220
253	0	23.4	0	0	0	0	0	0	0	1	1	0	0	0	0	0	0	0	0	0	0	0	0	3.8	0	1	3	5.2	17	1.10	6.8	4.2	195
254	0	23.4	0	0	1	0	0	0	0	2	0	1	0	1	0	0	0	0	0	0	0	0	0	5.0	0	0	1	5.0	20	1.06	8.0	4.0	167
254	0	23.4	0	0	1	0	0	0	0	2	0	1	0	1	0	0	0	0	0	0	0	0	0	5.0	0	0	2	6.2	18	1.05	7.7	4.4	190
254	0	23.4	0	0	1	0	0	0	0	2	0	1	0	1	0	0	0	0	0	0	0	0	0	5.0	0	0	3	6.3	19	1.02	7.6	4.3	168
255	0	22.4	0	1	0	0	0	0	0	0	0	0	0	0	0	0	0	0	0	0	0	0	0	5.1	0	0	1	5.1	13	1.00	8.0	4.0	194
255	0	22.4	0	1	0	0	0	0	0	0	0	0	0	0	0	0	0	0	0	0	0	0	0	5.1	0	0	2	5.7	18	1.00	7.4	4.4	190
255	0	22.4	0	1	0	0	0	0	0	0	0	0	0	0	0	0	0	0	0	0	0	0	0	5.1	0	0	3	5.9	20	1.00	7.5	4.3	209
256	0	24.7	0	0	0	1	0	1	0	1	0	0	0	0	1	0	0	0	0	0	0	0	0	3.4	0	1	1	3.4	16	1.10	7.0	4.0	228
256	0	24.7	0	0	0	1	0	1	0	1	0	0	0	0	1	0	0	0	0	0	0	0	0	3.4	0	1	2	4.1	13	1.05	7.2	4.4	190
256	0	24.7	0	0	0	1	0	1	0	1	0	0	0	0	1	0	0	0	0	0	0	0	0	3.4	0	1	3	3.7	15	0.97	7.0	4.3	203
257	0	25.1	0	1	1	0	0	0	0	2	0	0	0	0	0	0	0	0	0	0	0	0	0	5.5	0	0	1	5.5	13	1.20	7.0	5.0	157
257	0	25.1	0	1	1	0	0	0	0	2	0	0	0	0	0	0	0	0	0	0	0	0	0	5.5	0	0	2	5.2	14	1.10	6.7	4.2	158
257	0	25.1	0	1	1	0	0	0	0	2	0	0	0	0	0	0	0	0	0	0	0	0	0	5.5	0	0	3	5.2	13	0.92	6.9	4.4	194
258	0	23.1	1	0	0	0	0	0	0	0	0	0	0	0	0	0	0	0	0	0	0	0	0	5.1	0	0	1	5.1	12	1.00	7.0	4.0	199
258	0	23.1	1	0	0	0	0	0	0	0	0	0	0	0	0	0	0	0	0	0	0	0	0	5.1	0	0	2	4.2	17	0.80	6.7	4.4	169
258	0	23.1	1	0	0	0	0	0	0	0	0	0	0	0	0	0	0	0	0	0	0	0	0	5.1	0	0	3	5.2	17	0.60	6.8	4.2	215
259	0	24.9	0	0	0	0	0	1	0	0	0	0	0	0	1	0	0	0	0	0	0	0	0	5.0	0	0	1	5.0	11	1.10	7.0	5.0	180
259	0	24.9	0	0	0	0	0	1	0	0	0	0	0	0	1	0	0	0	0	0	0	0	0	5.0	0	0	2	4.1	14	1.00	7.1	4.6	198
259	0	24.9	0	0	0	0	0	1	0	0	0	0	0	0	1	0	0	0	0	0	0	0	0	5.0	0	0	3	4.1	13	0.94	7.2	4.7	146
260	0	26.5	1	1	0	0	0	1	0	0	0	0	0	0	1	0	0	0	0	0	0	0	0	5.9	0	0	1	5.9	14	1.03	7.0	4.0	153
260	0	26.5	1	1	0	0	0	1	0	0	0	0	0	0	1	0	0	0	0	0	0	0	0	5.9	0	0	2	6.6	19	0.95	6.6	4.1	132
260	0	26.5	1	1	0	0	0	1	0	0	0	0	0	0	1	0	0	0	0	0	0	0	0	5.9	0	0	3	5.8	17	0.84	6.8	4.2	121
261	1	22.1	0	0	0	0	0	0	1	2	0	0	0	0	0	1	1	0	1	0	1	0	0	4.6	0	0	1	4.6	24	1.20	7.0	4.0	165
261	1	22.1	0	0	0	0	0	0	1	2	0	0	0	0	0	1	1	0	1	0	1	0	0	4.6	0	0	2	5.5	14	1.14	6.4	3.6	185
261	1	22.1	0	0	0	0	0	0	1	2	0	0	0	0	0	1	1	0	1	0	1	0	0	4.6	0	0	3	4.4	14	1.10	5.2	2.9	181
262	0	24.3	1	0	1	0	0	1	0	1	0	0	0	1	1	0	0	0	0	0	0	0	0	3.9	0	1	1	3.9	13	1.05	7.0	4.0	170
262	0	24.3	1	0	1	0	0	1	0	1	0	0	0	1	1	0	0	0	0	0	0	0	0	3.9	0	1	2	4.7	23	0.87	7.2	4.1	171
262	0	24.3	1	0	1	0	0	1	0	1	0	0	0	1	1	0	0	0	0	0	0	0	0	3.9	0	1	3	4.4	14	0.95	7.4	4.5	187
263	0	23.4	0	0	0	0	0	0	0	1	0	0	0	0	0	0	0	0	0	0	0	0	0	5.5	0	0	1	5.5	14	1.10	7.0	4.0	192
263	0	23.4	0	0	0	0	0	0	0	1	0	0	0	0	0	0	0	0	0	0	0	0	0	5.5	0	0	2	6.0	17	1.11	6.6	4.1	216
263	0	23.4	0	0	0	0	0	0	0	1	0	0	0	0	0	0	0	0	0	0	0	0	0	5.5	0	0	3	6.2	23	1.18	6.6	4.1	193
264	0	26.2	1	0	0	0	0	0	0	0	0	0	0	0	0	0	0	0	0	0	0	0	0	6.3	0	0	1	6.3	14	1.04	7.0	5.0	181
264	0	26.2	1	0	0	0	0	0	0	0	0	0	0	0	0	0	0	0	0	0	0	0	0	6.3	0	0	2	4.4	13	1.05	6.9	4.5	185
264	0	26.2	1	0	0	0	0	0	0	0	0	0	0	0	0	0	0	0	0	0	0	0	0	6.3	0	0	3	7.0	13	0.87	6.8	4.4	182
265	0	26.3	1	1	1	0	0	0	0	2	1	0	0	1	0	0	0	0	0	0	0	0	0	4.3	0	0	1	4.3	18	1.00	7.0	5.0	160
265	0	26.3	1	1	1	0	0	0	0	2	1	0	0	1	0	0	0	0	0	0	0	0	0	4.3	0	0	2	3.9	16	1.06	7.1	4.5	177
265	0	26.3	1	1	1	0	0	0	0	2	1	0	0	1	0	0	0	0	0	0	0	0	0	4.3	0	0	3	5.5	12	0.94	7.1	4.6	199
266	0	25.8	1	1	0	0	0	0	0	1	0	0	0	0	0	0	0	0	0	0	0	0	1	5.4	0	0	1	5.4	16	0.83	8.0	4.0	206
266	0	25.8	1	1	0	0	0	0	0	1	0	0	0	0	0	0	0	0	0	0	0	0	1	5.4	0	0	2	5.1	11	0.80	7.3	4.1	177
266	0	25.8	1	1	0	0	0	0	0	1	0	0	0	0	0	0	0	0	0	0	0	0	1	5.4	0	0	3	5.6	11	0.76	7.2	4.3	206
267	0	28.1	1	1	1	0	0	1	0	2	1	0	0	1	1	0	0	0	0	0	0	0	0	6.4	0	0	1	6.4	16	0.98	8.0	5.0	177
267	0	28.1	1	1	1	0	0	1	0	2	1	0	0	1	1	0	0	0	0	0	0	0	0	6.4	0	0	2	7.1	13	0.99	7.4	4.8	156

id	Group	BMI	Current_ETOH	Ever_smoke	HTN	DM	CAD	DYSLIPID	Stage_4	ECOG	Con_asa	Con_thia	con_loopD	con_ARB	con_statin	Anti_Androgen	Bicalutamide	Cyproterone	GNRH	Leuprorelin	Goserelin	Triptorelin	Conc_RT	Base_UA	Base_hyperUA	Base_hypoUA	time	UA	BUN	CR	PROT	ALB	CHOL
267	0	28.1	1	1	1	0	0	1	0	2	1	0	0	1	1	0	0	0	0	0	0	0	0	6.4	0	0	3	6.1	13	1.01	6.8	4.6	149
268	1	22.7	0	0	0	0	0	0	0	0	0	0	0	0	0	0	0	0	1	0	1	0	0	6.8	0	0	1	6.8	19	1.10	7.0	4.0	212
268	1	22.7	0	0	0	0	0	0	0	0	0	0	0	0	0	0	0	0	1	0	1	0	0	6.8	0	0	2	5.3	16	0.90	7.0	4.1	241
268	1	22.7	0	0	0	0	0	0	0	0	0	0	0	0	0	0	0	0	1	0	1	0	0	6.8	0	0	3	5.4	21	0.90	7.3	4.3	246
269	0	22.9	1	0	1	0	0	0	0	2	0	0	0	0	0	0	0	0	0	0	0	0	0	5.4	0	0	1	5.4	20	1.04	7.0	4.0	225
269	0	22.9	1	0	1	0	0	0	0	2	0	0	0	0	0	0	0	0	0	0	0	0	0	5.4	0	0	2	5.8	18	1.04	7.0	4.4	219
269	0	22.9	1	0	1	0	0	0	0	2	0	0	0	0	0	0	0	0	0	0	0	0	0	5.4	0	0	3	5.8	22	1.03	7.5	4.4	238
270	1	25.5	0	1	0	0	0	0	0	2	1	0	0	0	1	1	1	0	1	0	1	0	0	7.6	1	0	1	7.6	18	1.30	7.0	4.0	251
270	1	25.5	0	1	0	0	0	0	0	2	1	0	0	0	1	1	1	0	1	0	1	0	0	7.6	1	0	2	6.6	18	1.20	7.1	4.3	215
270	1	25.5	0	1	0	0	0	0	0	2	1	0	0	0	1	1	1	0	1	0	1	0	0	7.6	1	0	3	6.0	16	1.13	7.1	4.3	239
271	0	30.0	1	1	0	0	0	1	0	2	0	0	0	0	0	0	0	0	0	0	0	0	0	7.1	1	0	1	7.1	13	0.95	7.0	4.0	198
271	0	30.0	1	1	0	0	0	1	0	2	0	0	0	0	0	0	0	0	0	0	0	0	0	7.1	1	0	2	6.9	15	0.90	7.3	4.4	178
271	0	30.0	1	1	0	0	0	1	0	2	0	0	0	0	0	0	0	0	0	0	0	0	0	7.1	1	0	3	6.3	15	0.89	7.4	4.5	154
272	1	22.3	1	0	1	0	0	1	0	2	0	1	0	1	1	1	1	0	1	0	1	0	0	5.6	0	0	1	5.6	14	1.01	8.0	4.0	203
272	1	22.3	1	0	1	0	0	1	0	2	0	1	0	1	1	1	1	0	1	0	1	0	0	5.6	0	0	2	6.8	18	0.99	8.3	4.5	226
272	1	22.3	1	0	1	0	0	1	0	2	0	1	0	1	1	1	1	0	1	0	1	0	0	5.6	0	0	3	6.4	15	0.95	8.1	4.5	180
273	0	16.6	0	1	0	0	0	0	0	2	0	0	0	0	0	0	0	0	0	0	0	0	0	4.9	0	0	1	4.9	18	1.14	6.0	4.0	174
273	0	16.6	0	1	0	0	0	0	0	2	0	0	0	0	0	0	0	0	0	0	0	0	0	4.9	0	0	2	5.0	20	1.12	6.1	3.9	161
273	0	16.6	0	1	0	0	0	0	0	2	0	0	0	0	0	0	0	0	0	0	0	0	0	4.9	0	0	3	5.0	15	0.95	6.3	3.9	164
274	0	28.4	1	1	0	0	0	0	0	1	0	0	0	0	0	0	0	0	0	0	0	0	0	5.4	0	0	1	5.4	13	1.13	7.0	4.0	242
274	0	28.4	1	1	0	0	0	0	0	1	0	0	0	0	0	0	0	0	0	0	0	0	0	5.4	0	0	2	6.4	16	0.94	6.9	4.2	232
274	0	28.4	1	1	0	0	0	0	0	1	0	0	0	0	0	0	0	0	0	0	0	0	0	5.4	0	0	3	6.5	18	1.02	6.5	3.9	220
275	1	22.3	0	1	1	0	0	0	0	1	0	0	0	0	0	1	1	0	0	0	0	0	1	7.5	1	0	1	7.5	19	0.89	7.0	4.0	148
275	1	22.3	0	1	1	0	0	0	0	1	0	0	0	0	0	1	1	0	0	0	0	0	1	7.5	1	0	2	6.3	16	0.84	6.8	4.2	174
275	1	22.3	0	1	1	0	0	0	0	1	0	0	0	0	0	1	1	0	0	0	0	0	1	7.5	1	0	3	6.7	21	0.75	7.3	4.4	203
276	0	23.1	0	0	0	0	0	0	0	0	0	0	0	0	0	0	0	0	0	0	0	0	0	5.1	0	0	1	5.1	15	1.30	7.0	4.0	169
276	0	23.1	0	0	0	0	0	0	0	0	0	0	0	0	0	0	0	0	0	0	0	0	0	5.1	0	0	2	4.4	15	1.17	7.1	4.4	175
276	0	23.1	0	0	0	0	0	0	0	0	0	0	0	0	0	0	0	0	0	0	0	0	0	5.1	0	0	3	5.0	19	1.12	7.3	4.4	154
277	0	22.9	0	0	1	0	0	0	0	1	0	0	0	1	0	0	0	0	0	0	0	0	0	6.1	0	0	1	6.1	12	1.07	7.0	4.0	171
277	0	22.9	0	0	1	0	0	0	0	1	0	0	0	1	0	0	0	0	0	0	0	0	0	6.1	0	0	2	5.6	9	0.99	7.4	4.2	163
277	0	22.9	0	0	1	0	0	0	0	1	0	0	0	1	0	0	0	0	0	0	0	0	0	6.1	0	0	3	5.0	11	1.02	7.4	4.2	163
278	0	25.0	1	0	1	0	0	0	0	2	0	0	0	0	0	0	0	0	0	0	0	0	0	5.9	0	0	1	5.9	17	1.11	7.0	4.0	147
278	0	25.0	1	0	1	0	0	0	0	2	0	0	0	0	0	0	0	0	0	0	0	0	0	5.9	0	0	2	5.4	14	1.06	7.0	4.6	150
278	0	25.0	1	0	1	0	0	0	0	2	0	0	0	0	0	0	0	0	0	0	0	0	0	5.9	0	0	3	5.2	15	1.01	7.2	4.6	163
279	0	22.6	1	0	1	0	0	0	0	1	1	0	0	0	0	0	0	0	0	0	0	0	0	4.7	0	0	1	4.7	18	0.96	6.0	4.0	171
279	0	22.6	1	0	1	0	0	0	0	1	1	0	0	0	0	0	0	0	0	0	0	0	0	4.7	0	0	2	5.9	16	0.98	6.8	4.2	138
279	0	22.6	1	0	1	0	0	0	0	1	1	0	0	0	0	0	0	0	0	0	0	0	0	4.7	0	0	3	4.5	22	1.00	7.1	4.4	154
280	0	23.1	0	1	0	0	0	1	0	0	0	0	0	0	1	0	0	0	0	0	0	0	0	5.7	0	0	1	5.7	18	0.79	7.0	4.0	175
280	0	23.1	0	1	0	0	0	1	0	0	0	0	0	0	1	0	0	0	0	0	0	0	0	5.7	0	0	2	6.2	26	0.78	7.1	4.2	170
280	0	23.1	0	1	0	0	0	1	0	0	0	0	0	0	1	0	0	0	0	0	0	0	0	5.7	0	0	3	6.1	12	0.75	7.4	4.2	131
281	1	23.9	1	0	1	0	0	0	0	1	0	1	0	1	0	1	1	0	0	0	0	0	0	7.1	1	0	1	7.1	17	0.95	8.0	5.0	216
281	1	23.9	1	0	1	0	0	0	0	1	0	1	0	1	0	1	1	0	0	0	0	0	0	7.1	1	0	2	7.2	14	0.98	7.5	4.8	179
281	1	23.9	1	0	1	0	0	0	0	1	0	1	0	1	0	1	1	0	0	0	0	0	0	7.1	1	0	3	6.0	11	1.00	7.5	4.8	194
282	1	21.8	0	0	1	0	0	0	1	2	0	0	0	1	0	1	1	0	1	0	1	0	0	4.0	0	0	1	4.0	10	0.97	7.0	5.0	145
282	1	21.8	0	0	1	0	0	0	1	2	0	0	0	1	0	1	1	0	1	0	1	0	0	4.0	0	0	2	4.1	12	0.90	7.2	4.8	167
282	1	21.8	0	0	1	0	0	0	1	2	0	0	0	1	0	1	1	0	1	0	1	0	0	4.0	0	0	3	2.6	13	0.79	7.2	4.8	136
283	0	21.0	0	0	1	0	0	0	0	2	0	0	0	0	0	0	0	0	0	0	0	0	0	5.4	0	0	1	5.4	17	0.88	7.0	5.0	211
283	0	21.0	0	0	1	0	0	0	0	2	0	0	0	0	0	0	0	0	0	0	0	0	0	5.4	0	0	2	3.5	15	0.85	7.1	4.2	172
283	0	21.0	0	0	1	0	0	0	0	2	0	0	0	0	0	0	0	0	0	0	0	0	0	5.4	0	0	3	6.1	15	0.82	7.0	4.3	177
284	0	24.8	0	1	1	0	0	0	0	2	1	0	0	1	0	0	0	0	0	0	0	0	0	6.6	0	0	1	6.6	17	0.89	7.0	5.0	150

data_ADT_SUA

id	Group	BMI	Current_ETOH	Ever_smoke	HTN	DM	CAD	DYSLIPID	Stage_4	ECOG	Con_asa	Con_thia	con_loopD	con_ARB	con_statin	Anti_Androgen	Bicalutamide	Cyproterone	GNRH	Leuprorelin	Goserelin	Triptorelin	Conc_RT	Base_UA	Base_hyperUA	Base_hypoUA	time	UA	BUN	CR	PROT	ALB	CHOL
284	0	24.8	0	1	1	0	0	0	0	2	1	0	0	1	0	0	0	0	0	0	0	0	0	6.6	0	0	2	6.5	16	0.90	6.7	4.5	151
284	0	24.8	0	1	1	0	0	0	0	2	1	0	0	1	0	0	0	0	0	0	0	0	0	6.6	0	0	3	5.7	15	0.92	6.5	4.3	144
285	1	24.7	1	1	1	0	0	1	0	1	0	1	0	0	1	1	1	0	0	0	0	0	1	4.7	0	0	1	4.7	14	0.98	7.0	4.0	190
285	1	24.7	1	1	1	0	0	1	0	1	0	1	0	0	1	1	1	0	0	0	0	0	1	4.7	0	0	2	5.4	12	1.03	6.9	4.5	210
285	1	24.7	1	1	1	0	0	1	0	1	0	1	0	0	1	1	1	0	0	0	0	0	1	4.7	0	0	3	3.8	17	1.11	6.9	4.5	211
286	1	21.8	0	1	1	0	0	0	0	1	0	1	0	0	0	1	1	0	0	0	0	0	1	6.6	0	0	1	6.6	18	0.96	6.0	4.0	162
286	1	21.8	0	1	1	0	0	0	0	1	0	1	0	0	0	1	1	0	0	0	0	0	1	6.6	0	0	2	5.0	21	1.01	6.4	3.8	148
286	1	21.8	0	1	1	0	0	0	0	1	0	1	0	0	0	1	1	0	0	0	0	0	1	6.6	0	0	3	6.0	16	1.05	6.1	3.8	152
287	0	25.2	1	1	0	0	0	0	0	0	0	0	0	0	0	0	0	0	0	0	0	0	0	6.9	0	0	1	6.9	17	0.99	7.0	4.0	176
287	0	25.2	1	1	0	0	0	0	0	0	0	0	0	0	0	0	0	0	0	0	0	0	0	6.9	0	0	2	7.0	15	0.94	7.3	4.4	184
287	0	25.2	1	1	0	0	0	0	0	0	0	0	0	0	0	0	0	0	0	0	0	0	0	6.9	0	0	3	6.7	16	0.88	7.0	4.2	185
288	0	31.7	1	0	1	1	0	0	0	1	0	1	0	1	0	0	0	0	0	0	0	0	0	4.8	0	0	1	4.8	17	1.08	7.0	5.0	208
288	0	31.7	1	0	1	1	0	0	0	1	0	1	0	1	0	0	0	0	0	0	0	0	0	4.8	0	0	2	5.0	19	0.90	7.1	4.4	140
288	0	31.7	1	0	1	1	0	0	0	1	0	1	0	1	0	0	0	0	0	0	0	0	0	4.8	0	0	3	4.1	28	0.98	6.9	4.5	168
289	0	24.6	0	0	0	0	0	0	0	0	0	0	0	0	0	0	0	0	0	0	0	0	0	7.1	1	0	1	7.1	15	0.93	8.0	5.0	244
289	0	24.6	0	0	0	0	0	0	0	0	0	0	0	0	0	0	0	0	0	0	0	0	0	7.1	1	0	2	7.6	20	0.82	7.8	4.5	257
289	0	24.6	0	0	0	0	0	0	0	0	0	0	0	0	0	0	0	0	0	0	0	0	0	7.1	1	0	3	6.5	16	0.81	8.0	4.6	235
290	0	24.2	1	1	1	0	0	0	0	1	0	0	0	1	0	0	0	0	0	0	0	0	0	4.9	0	0	1	4.9	15	0.84	7.0	4.0	99
290	0	24.2	1	1	1	0	0	0	0	1	0	0	0	1	0	0	0	0	0	0	0	0	0	4.9	0	0	2	5.6	18	0.87	6.9	4.3	91
290	0	24.2	1	1	1	0	0	0	0	1	0	0	0	1	0	0	0	0	0	0	0	0	0	4.9	0	0	3	5.7	14	0.89	6.3	3.8	96
291	0	25.3	0	1	0	0	0	0	0	0	0	0	0	0	0	0	0	0	0	0	0	0	0	9.1	1	0	1	9.1	23	0.85	7.0	4.0	172
291	0	25.3	0	1	0	0	0	0	0	0	0	0	0	0	0	0	0	0	0	0	0	0	0	9.1	1	0	2	8.2	26	0.85	6.8	4.4	206
291	0	25.3	0	1	0	0	0	0	0	0	0	0	0	0	0	0	0	0	0	0	0	0	0	9.1	1	0	3	8.7	23	0.85	7.1	4.1	209
292	0	27.4	0	0	0	0	0	0	0	0	0	0	0	1	1	0	0	0	0	0	0	0	0	5.2	0	0	1	5.2	15	0.91	7.0	4.0	228
292	0	27.4	0	0	0	0	0	0	0	0	0	0	0	1	1	0	0	0	0	0	0	0	0	5.2	0	0	2	5.2	16	0.92	7.3	4.5	232
292	0	27.4	0	0	0	0	0	0	0	0	0	0	0	1	1	0	0	0	0	0	0	0	0	5.2	0	0	3	5.5	10	0.88	7.3	4.6	216
293	0	21.4	1	0	0	0	0	0	0	0	0	0	0	0	0	0	0	0	0	0	0	0	0	5.2	0	0	1	5.2	7	0.88	7.0	4.0	148
293	0	21.4	1	0	0	0	0	0	0	0	0	0	0	0	0	0	0	0	0	0	0	0	0	5.2	0	0	2	4.7	9	0.81	6.7	3.8	136
293	0	21.4	1	0	0	0	0	0	0	0	0	0	0	0	0	0	0	0	0	0	0	0	0	5.2	0	0	3	5.5	9	0.74	7.8	4.6	170
294	0	25.6	1	1	1	0	0	0	0	2	0	0	0	0	0	0	0	0	0	0	0	0	0	6.5	0	0	1	6.5	13	0.99	7.0	4.0	214
294	0	25.6	1	1	1	0	0	0	0	2	0	0	0	0	0	0	0	0	0	0	0	0	0	6.5	0	0	2	5.2	12	0.88	7.3	4.5	195
294	0	25.6	1	1	1	0	0	0	0	2	0	0	0	0	0	0	0	0	0	0	0	0	0	6.5	0	0	3	5.3	16	0.90	7.4	4.6	209
295	0	27.8	0	0	0	0	0	0	0	0	0	0	0	0	0	0	0	0	0	0	0	0	0	7.0	1	0	1	7.0	10	0.84	7.0	4.0	186
295	0	27.8	0	0	0	0	0	0	0	0	0	0	0	0	0	0	0	0	0	0	0	0	0	7.0	1	0	2	6.3	12	0.74	7.2	4.3	181
295	0	27.8	0	0	0	0	0	0	0	0	0	0	0	0	0	0	0	0	0	0	0	0	0	7.0	1	0	3	6.7	14	0.60	7.1	4.2	166
296	0	23.7	0	0	0	0	0	0	0	0	0	0	0	0	0	0	0	0	0	0	0	0	0	6.4	0	0	1	6.4	7	1.12	7.0	4.0	146
296	0	23.7	0	0	0	0	0	0	0	0	0	0	0	0	0	0	0	0	0	0	0	0	0	6.4	0	0	2	6.0	15	1.06	7.3	4.5	170
296	0	23.7	0	0	0	0	0	0	0	0	0	0	0	0	0	0	0	0	0	0	0	0	0	6.4	0	0	3	6.9	17	0.96	7.1	4.5	167
297	0	24.6	1	0	1	0	0	1	0	2	1	1	0	1	1	0	0	0	0	0	0	0	0	8.4	1	0	1	8.4	12	0.90	7.0	4.0	133
297	0	24.6	1	0	1	0	0	1	0	2	1	1	0	1	1	0	0	0	0	0	0	0	0	8.4	1	0	2	7.9	14	0.88	7.0	4.2	178
297	0	24.6	1	0	1	0	0	1	0	2	1	1	0	1	1	0	0	0	0	0	0	0	0	8.4	1	0	3	8.1	16	0.86	6.9	4.3	184
298	1	25.6	1	1	0	0	0	0	0	1	0	0	0	0	0	1	1	0	0	0	0	0	1	5.5	0	0	1	5.5	18	0.75	7.0	4.0	192
298	1	25.6	1	1	0	0	0	0	0	1	0	0	0	0	0	1	1	0	0	0	0	0	1	5.5	0	0	2	5.4	19	0.80	7.1	4.5	196
298	1	25.6	1	1	0	0	0	0	0	1	0	0	0	0	0	1	1	0	0	0	0	0	1	5.5	0	0	3	6.0	16	0.90	7.0	4.4	155
299	0	22.1	1	1	0	0	0	0	0	0	0	0	0	0	0	0	0	0	0	0	0	0	0	6.0	0	0	1	6.0	16	0.95	8.0	5.0	200
299	0	22.1	1	1	0	0	0	0	0	0	0	0	0	0	0	0	0	0	0	0	0	0	0	6.0	0	0	2	5.5	14	0.90	7.7	4.9	221
299	0	22.1	1	1	0	0	0	0	0	0	0	0	0	0	0	0	0	0	0	0	0	0	0	6.0	0	0	3	5.0	14	0.89	7.2	4.5	196
300	1	22.8	0	1	1	0	0	0	0	1	0	0	0	0	0	1	1	0	0	0	0	0	1	7.4	1	0	1	7.4	14	0.81	7.0	5.0	139
300	1	22.8	0	1	1	0	0	0	0	1	0	0	0	0	0	1	1	0	0	0	0	0	1	7.4	1	0	2	5.0	17	0.85	7.1	4.7	176
300	1	22.8	0	1	1	0	0	0	0	1	0	0	0	0	0	1	1	0	0	0	0	0	1	7.4	1	0	3	4.8	11	1.06	6.7	4.3	141

data_ADT_SUA

id	Group	BMI	Current_ETOH	Ever_smoke	HTN	DM	CAD	DYSLIPID	Stage_4	ECOG	Con_asa	Con_thia	con_loopD	con_ARB	con_statin	Anti_Androgen	Bicalutamide	Cyproterone	GNRH	Leuprorelin	Goserelin	Triptorelin	Conc_RT	Base_UA	Base_hyperUA	Base_hypoUA	time	UA	BUN	CR	PROT	ALB	CHOL
301	1	21.5	1	0	1	0	0	1	1	1	0	0	0	1	1	1	1	0	1	0	1	0	1	5.8	0	0	1	5.8	10	0.89	8.0	5.0	192
301	1	21.5	1	0	1	0	0	1	1	1	0	0	0	1	1	1	1	0	1	0	1	0	1	5.8	0	0	2	4.4	15	0.90	7.6	4.8	165
301	1	21.5	1	0	1	0	0	1	1	1	0	0	0	1	1	1	1	0	1	0	1	0	1	5.8	0	0	3	5.6	17	0.89	7.4	4.8	155
302	0	22.9	0	0	1	1	0	1	0	1	1	1	0	1	1	0	0	0	0	0	0	0	0	4.6	0	0	1	4.6	14	0.70	6.0	4.0	134
302	0	22.9	0	0	1	1	0	1	0	1	1	1	0	1	1	0	0	0	0	0	0	0	0	4.6	0	0	2	5.4	11	0.68	7.1	4.5	148
302	0	22.9	0	0	1	1	0	1	0	1	1	1	0	1	1	0	0	0	0	0	0	0	0	4.6	0	0	3	5.7	16	0.66	6.9	4.4	135
303	0	19.8	1	1	1	0	0	1	0	0	0	1	0	1	1	0	0	0	0	0	0	0	0	4.1	0	0	1	4.1	11	1.12	7.0	4.0	159
303	0	19.8	1	1	1	0	0	1	0	0	0	1	0	1	1	0	0	0	0	0	0	0	0	4.1	0	0	2	4.5	9	1.19	7.2	4.4	145
303	0	19.8	1	1	1	0	0	1	0	0	0	1	0	1	1	0	0	0	0	0	0	0	0	4.1	0	0	3	3.9	13	1.10	7.0	4.3	163
304	0	23.3	1	0	1	0	0	1	0	1	0	1	0	1	1	0	0	0	0	0	0	0	0	6.5	0	0	1	6.5	17	1.09	8.0	5.0	146
304	0	23.3	1	0	1	0	0	1	0	1	0	1	0	1	1	0	0	0	0	0	0	0	0	6.5	0	0	2	5.7	22	1.02	7.1	4.4	172
304	0	23.3	1	0	1	0	0	1	0	1	0	1	0	1	1	0	0	0	0	0	0	0	0	6.5	0	0	3	6.3	19	0.94	7.2	4.5	151
305	1	28.4	0	0	1	0	0	0	0	2	1	0	0	1	0	1	1	0	1	1	0	0	1	6.0	0	0	1	6.0	13	0.84	7.0	4.0	158
305	1	28.4	0	0	1	0	0	0	0	2	1	0	0	1	0	1	1	0	1	1	0	0	1	6.0	0	0	2	7.0	17	0.82	7.9	4.6	210
305	1	28.4	0	0	1	0	0	0	0	2	1	0	0	1	0	1	1	0	1	1	0	0	1	6.0	0	0	3	4.8	14	0.84	7.9	4.6	214
306	1	27.6	0	1	0	0	0	0	1	1	0	0	0	0	0	1	1	0	1	0	1	0	1	6.9	0	0	1	6.9	13	0.73	7.0	4.0	169
306	1	27.6	0	1	0	0	0	0	1	1	0	0	0	0	0	1	1	0	1	0	1	0	1	6.9	0	0	2	5.4	17	0.86	7.7	4.8	277
306	1	27.6	0	1	0	0	0	0	1	1	0	0	0	0	0	1	1	0	1	0	1	0	1	6.9	0	0	3	5.2	19	0.71	7.2	4.5	228
307	0	21.7	0	0	0	0	0	0	0	0	0	0	0	0	0	0	0	0	0	0	0	0	0	4.1	0	0	1	4.1	21	1.00	6.0	4.0	133
307	0	21.7	0	0	0	0	0	0	0	0	0	0	0	0	0	0	0	0	0	0	0	0	0	4.1	0	0	2	5.3	17	0.94	6.4	4.0	152
307	0	21.7	0	0	0	0	0	0	0	0	0	0	0	0	0	0	0	0	0	0	0	0	0	4.1	0	0	3	5.1	19	0.87	6.9	4.2	151
308	0	24.8	1	0	0	0	0	0	0	0	0	0	0	0	0	0	0	0	0	0	0	0	0	6.5	0	0	1	6.5	10	0.83	8.0	4.0	161
308	0	24.8	1	0	0	0	0	0	0	0	0	0	0	0	0	0	0	0	0	0	0	0	0	6.5	0	0	2	6.9	7	0.69	8.0	4.5	154
308	0	24.8	1	0	0	0	0	0	0	0	0	0	0	0	0	0	0	0	0	0	0	0	0	6.5	0	0	3	6.4	10	0.82	8.2	4.1	155
309	0	21.1	0	1	1	0	0	0	0	2	0	0	0	0	0	0	0	0	0	0	0	0	0	5.2	0	0	1	5.2	13	1.02	7.0	4.0	162
309	0	21.1	0	1	1	0	0	0	0	2	0	0	0	0	0	0	0	0	0	0	0	0	0	5.2	0	0	2	5.8	20	0.88	8.1	4.5	187
309	0	21.1	0	1	1	0	0	0	0	2	0	0	0	0	0	0	0	0	0	0	0	0	0	5.2	0	0	3	5.8	14	0.75	7.6	4.4	194
310	0	23.5	1	1	0	0	0	0	0	0	0	0	0	0	0	0	0	0	0	0	0	0	0	5.3	0	0	1	5.3	15	1.17	7.0	4.0	173
310	0	23.5	1	1	0	0	0	0	0	0	0	0	0	0	0	0	0	0	0	0	0	0	0	5.3	0	0	2	5.8	18	1.10	6.9	4.3	173
310	0	23.5	1	1	0	0	0	0	0	0	0	0	0	0	0	0	0	0	0	0	0	0	0	5.3	0	0	3	5.5	20	1.03	7.1	4.3	169
311	1	21.6	0	0	0	0	0	0	1	1	1	0	0	0	1	1	1	0	1	0	1	0	0	5.8	0	0	1	5.8	20	0.93	6.0	4.0	202
311	1	21.6	0	0	0	0	0	0	1	1	1	0	0	0	1	1	1	0	1	0	1	0	0	5.8	0	0	2	6.8	25	0.96	6.9	4.2	294
311	1	21.6	0	0	0	0	0	0	1	1	1	0	0	0	1	1	1	0	1	0	1	0	0	5.8	0	0	3	7.2	31	0.87	7.4	4.5	287
312	0	24.9	1	0	1	0	0	0	0	1	0	1	0	1	0	0	0	0	0	0	0	0	0	7.5	1	0	1	7.5	14	0.99	8.0	5.0	184
312	0	24.9	1	0	1	0	0	0	0	1	0	1	0	1	0	0	0	0	0	0	0	0	0	7.5	1	0	2	8.9	12	0.93	7.7	4.7	178
312	0	24.9	1	0	1	0	0	0	0	1	0	1	0	1	0	0	0	0	0	0	0	0	0	7.5	1	0	3	8.9	11	0.57	7.8	4.7	202
313	1	26.8	1	1	0	0	0	0	0	1	0	0	0	0	0	0	0	0	1	1	0	0	1	4.7	0	0	1	4.7	13	0.83	7.0	4.0	246
313	1	26.8	1	1	0	0	0	0	0	1	0	0	0	0	0	0	0	0	1	1	0	0	1	4.7	0	0	2	3.9	13	0.83	6.8	4.1	236
313	1	26.8	1	1	0	0	0	0	0	1	0	0	0	0	0	0	0	0	1	1	0	0	1	4.7	0	0	3	3.7	14	0.74	6.7	4.0	230
314	1	21.8	0	1	1	0	0	0	0	1	0	0	0	1	0	1	1	0	0	0	0	0	1	4.9	0	0	1	4.9	20	0.91	7.0	5.0	182
314	1	21.8	0	1	1	0	0	0	0	1	0	0	0	1	0	1	1	0	0	0	0	0	1	4.9	0	0	2	4.5	16	1.00	6.6	4.4	168
314	1	21.8	0	1	1	0	0	0	0	1	0	0	0	1	0	1	1	0	0	0	0	0	1	4.9	0	0	3	4.8	10	0.89	6.9	4.6	178
315	0	25.2	0	1	1	0	0	1	0	2	0	0	0	0	1	0	0	0	0	0	0	0	0	5.9	0	0	1	5.9	12	0.77	7.0	4.0	167
315	0	25.2	0	1	1	0	0	1	0	2	0	0	0	0	1	0	0	0	0	0	0	0	0	5.9	0	0	2	7.1	16	0.71	7.2	4.4	151
315	0	25.2	0	1	1	0	0	1	0	2	0	0	0	0	1	0	0	0	0	0	0	0	0	5.9	0	0	3	7.7	11	0.67	7.4	4.5	174
316	0	23.9	1	0	1	1	0	0	0	1	0	0	0	1	0	0	0	0	0	0	0	0	0	4.9	0	0	1	4.9	24	0.78	7.0	4.0	142
316	0	23.9	1	0	1	1	0	0	0	1	0	0	0	1	0	0	0	0	0	0	0	0	0	4.9	0	0	2	5.6	18	0.77	7.7	4.6	197
316	0	23.9	1	0	1	1	0	0	0	1	0	0	0	1	0	0	0	0	0	0	0	0	0	4.9	0	0	3	5.8	25	0.71	7.5	4.5	185
317	0	28.0	1	1	0	0	0	0	0	1	0	0	0	0	0	0	0	0	0	0	0	0	0	5.9	0	0	1	5.9	16	1.02	7.0	4.0	194
317	0	28.0	1	1	0	0	0	0	0	1	0	0	0	0	0	0	0	0	0	0	0	0	0	5.9	0	0	2	6.3	18	1.00	7.2	4.4	221

id	Group	BMI	Current_ETOH	Ever_smoke	HTN	DM	CAD	DYSLIPID	Stage_4	ECOG	Con_asa	Con_thia	con_loopD	con_ARB	con_statin	Anti_Androgen	Bicalutamide	Cyproterone	GNRH	Leuprorelin	Goserelin	Triptorelin	Conc_RT	Base_UA	Base_hyperUA	Base_hypoUA	time	UA	BUN	CR	PROT	ALB	CHOL
317	0	28.0	1	1	0	0	0	0	0	1	0	0	0	0	0	0	0	0	0	0	0	0	0	5.9	0	0	3	6.3	15	1.01	7.3	4.6	188
318	1	21.3	1	1	1	1	0	0	1	4	0	0	0	1	0	1	1	0	1	0	1	0	1	2.2	0	1	1	2.2	3	0.98	7.0	5.0	225
318	1	21.3	1	1	1	1	0	0	1	4	0	0	0	1	0	1	1	0	1	0	1	0	1	2.2	0	1	2	3.5	8	1.00	7.9	4.0	202
318	1	21.3	1	1	1	1	0	0	1	4	0	0	0	1	0	1	1	0	1	0	1	0	1	2.2	0	1	3	3.1	6	0.96	6.8	3.7	169
319	1	24.5	0	1	0	0	0	0	1	0	0	0	0	0	0	1	1	0	1	0	1	0	0	2.6	0	1	1	2.6	16	0.90	7.0	4.0	185
319	1	24.5	0	1	0	0	0	0	1	0	0	0	0	0	0	1	1	0	1	0	1	0	0	2.6	0	1	2	2.3	20	0.90	7.1	4.2	187
319	1	24.5	0	1	0	0	0	0	1	0	0	0	0	0	0	1	1	0	1	0	1	0	0	2.6	0	1	3	2.2	17	0.89	6.9	4.1	212
320	0	26.1	0	0	0	0	0	0	0	3	0	0	0	1	0	0	0	0	0	0	0	0	0	6.3	0	0	1	6.3	19	0.97	7.0	4.0	186
320	0	26.1	0	0	0	0	0	0	0	3	0	0	0	1	0	0	0	0	0	0	0	0	0	6.3	0	0	2	5.9	19	0.89	6.4	4.4	172
320	0	26.1	0	0	0	0	0	0	0	3	0	0	0	1	0	0	0	0	0	0	0	0	0	6.3	0	0	3	5.4	14	0.72	6.5	4.3	148
321	0	26.6	0	1	1	1	0	1	0	2	1	0	1	1	1	0	0	0	0	0	0	0	0	6.9	0	0	1	6.9	17	0.82	8.0	4.0	173
321	0	26.6	0	1	1	1	0	1	0	2	1	0	1	1	1	0	0	0	0	0	0	0	0	6.9	0	0	2	6.4	14	1.04	7.2	4.0	153
321	0	26.6	0	1	1	1	0	1	0	2	1	0	1	1	1	0	0	0	0	0	0	0	0	6.9	0	0	3	7.0	16	0.81	7.5	4.2	162
322	0	26.6	1	0	1	0	0	1	0	2	0	0	0	1	1	0	0	0	0	0	0	0	0	6.8	0	0	1	6.8	16	0.95	7.0	4.0	180
322	0	26.6	1	0	1	0	0	1	0	2	0	0	0	1	1	0	0	0	0	0	0	0	0	6.8	0	0	2	6.9	26	0.75	7.5	4.4	164
322	0	26.6	1	0	1	0	0	1	0	2	0	0	0	1	1	0	0	0	0	0	0	0	0	6.8	0	0	3	5.8	16	0.94	7.2	4.4	174
323	0	27.0	0	1	0	0	0	0	0	2	0	0	0	0	0	0	0	0	0	0	0	0	0	6.6	0	0	1	6.6	17	1.10	7.0	4.0	163
323	0	27.0	0	1	0	0	0	0	0	2	0	0	0	0	0	0	0	0	0	0	0	0	0	6.6	0	0	2	5.9	12	1.06	7.2	4.5	184
323	0	27.0	0	1	0	0	0	0	0	2	0	0	0	0	0	0	0	0	0	0	0	0	0	6.6	0	0	3	7.2	14	1.02	7.3	4.5	186
324	1	24.8	0	1	0	0	0	0	0	1	0	0	0	0	0	1	1	0	0	0	0	0	1	7.7	1	0	1	7.7	16	0.88	7.0	4.0	179
324	1	24.8	0	1	0	0	0	0	0	1	0	0	0	0	0	1	1	0	0	0	0	0	1	7.7	1	0	2	6.6	17	0.89	7.2	4.3	189
324	1	24.8	0	1	0	0	0	0	0	1	0	0	0	0	0	1	1	0	0	0	0	0	1	7.7	1	0	3	6.8	18	0.96	6.9	4.1	186
325	1	23.8	0	1	0	0	0	0	0	1	0	0	0	0	0	1	1	0	0	0	0	0	1	7.2	1	0	1	7.2	18	0.96	7.0	4.0	195
325	1	23.8	0	1	0	0	0	0	0	1	0	0	0	0	0	1	1	0	0	0	0	0	1	7.2	1	0	2	5.9	19	0.99	7.6	4.9	281
325	1	23.8	0	1	0	0	0	0	0	1	0	0	0	0	0	1	1	0	0	0	0	0	1	7.2	1	0	3	5.4	17	1.02	7.1	4.4	251
326	1	24.5	1	1	1	1	0	0	0	2	0	1	0	0	0	1	1	0	1	0	1	0	0	7.5	1	0	1	7.5	16	0.86	9.0	4.0	172
326	1	24.5	1	1	1	1	0	0	0	2	0	1	0	0	0	1	1	0	1	0	1	0	0	7.5	1	0	2	6.8	16	0.83	8.1	4.0	171
326	1	24.5	1	1	1	1	0	0	0	2	0	1	0	0	0	1	1	0	1	0	1	0	0	7.5	1	0	3	7.7	21	0.74	8.2	4.1	160
327	0	21.4	0	0	0	0	0	0	0	0	0	0	0	0	0	0	0	0	0	0	0	0	0	5.0	0	0	1	5.0	14	0.86	6.0	4.0	175
327	0	21.4	0	0	0	0	0	0	0	0	0	0	0	0	0	0	0	0	0	0	0	0	0	5.0	0	0	2	4.6	11	0.91	7.3	4.7	232
327	0	21.4	0	0	0	0	0	0	0	0	0	0	0	0	0	0	0	0	0	0	0	0	0	5.0	0	0	3	4.3	18	0.94	7.4	4.6	243
328	1	30.0	1	0	1	0	0	0	1	2	0	0	0	0	0	1	1	0	1	0	1	0	0	3.6	0	1	1	3.6	20	1.16	8.0	5.0	192
328	1	30.0	1	0	1	0	0	0	1	2	0	0	0	0	0	1	1	0	1	0	1	0	0	3.6	0	1	2	3.6	19	0.92	7.4	4.6	193
328	1	30.0	1	0	1	0	0	0	1	2	0	0	0	0	0	1	1	0	1	0	1	0	0	3.6	0	1	3	3.8	21	1.00	7.3	4.5	167
329	1	19.9	1	1	0	0	0	0	0	1	0	0	0	0	0	1	1	0	0	0	0	0	1	6.7	0	0	1	6.7	15	1.03	7.0	4.0	190
329	1	19.9	1	1	0	0	0	0	0	1	0	0	0	0	0	1	1	0	0	0	0	0	1	6.7	0	0	2	6.0	11	1.01	7.2	4.0	201
329	1	19.9	1	1	0	0	0	0	0	1	0	0	0	0	0	1	1	0	0	0	0	0	1	6.7	0	0	3	5.4	12	0.96	7.0	4.1	200
330	0	22.8	1	0	1	0	0	0	0	1	0	0	0	1	1	0	0	0	0	0	0	0	0	5.6	0	0	1	5.6	17	0.76	7.0	4.0	141
330	0	22.8	1	0	1	0	0	0	0	1	0	0	0	1	1	0	0	0	0	0	0	0	0	5.6	0	0	2	5.3	17	0.74	6.8	4.2	110
330	0	22.8	1	0	1	0	0	0	0	1	0	0	0	1	1	0	0	0	0	0	0	0	0	5.6	0	0	3	4.5	13	0.70	6.9	4.4	127
331	0	22.9	1	1	1	1	0	0	0	2	0	0	0	0	0	0	0	0	0	0	0	0	0	5.9	0	0	1	5.9	16	1.09	7.0	4.0	147
331	0	22.9	1	1	1	1	0	0	0	2	0	0	0	0	0	0	0	0	0	0	0	0	0	5.9	0	0	2	4.9	13	1.09	7.2	4.5	144
331	0	22.9	1	1	1	1	0	0	0	2	0	0	0	0	0	0	0	0	0	0	0	0	0	5.9	0	0	3	4.2	13	1.12	7.3	4.6	132
332	0	22.6	1	0	1	0	0	0	0	2	0	0	0	0	0	0	0	0	0	0	0	0	0	5.6	0	0	1	5.6	15	0.90	7.0	4.0	208
332	0	22.6	1	0	1	0	0	0	0	2	0	0	0	0	0	0	0	0	0	0	0	0	0	5.6	0	0	2	6.1	16	0.88	7.8	4.6	206
332	0	22.6	1	0	1	0	0	0	0	2	0	0	0	0	0	0	0	0	0	0	0	0	0	5.6	0	0	3	6.5	11	0.84	6.8	4.0	178
333	1	23.9	0	0	1	0	0	0	1	1	0	1	0	1	0	1	1	0	1	0	0	1	0	5.9	0	0	1	5.9	20	1.11	7.0	4.0	179
333	1	23.9	0	0	1	0	0	0	1	1	0	1	0	1	0	1	1	0	1	0	0	1	0	5.9	0	0	2	5.3	18	1.09	7.2	4.2	201
333	1	23.9	0	0	1	0	0	0	1	1	0	1	0	1	0	1	1	0	1	0	0	1	0	5.9	0	0	3	5.4	32	1.02	7.1	4.3	190
334	1	22.1	0	0	0	0	0	0	0	1	0	0	0	0	0	1	1	0	0	0	0	0	1	4.7	0	0	1	4.7	14	0.89	7.0	4.0	160

id	Group	BMI	Current_ETOH	Ever_smoke	HTN	DM	CAD	DYSLIPID	Stage_4	ECOG	Con_asa	Con_thia	con_loopD	con_ARB	con_statin	Anti_Androgen	Bicalutamide	Cyproterone	GNRH	Leuprorelin	Goserelin	Triptorelin	Conc_RT	Base_UA	Base_hyperUA	Base_hypoUA	time	UA	BUN	CR	PROT	ALB	CHOL
334	1	22.1	0	0	0	0	0	0	0	1	0	0	0	0	0	1	1	0	0	0	0	0	1	4.7	0	0	2	3.8	11	0.87	7.0	4.3	158
334	1	22.1	0	0	0	0	0	0	0	1	0	0	0	0	0	1	1	0	0	0	0	0	1	4.7	0	0	3	3.9	16	0.84	7.3	4.3	161
335	0	22.6	0	0	0	0	0	0	0	1	0	0	0	0	0	0	0	0	0	0	0	0	0	3.9	0	1	1	3.9	12	0.98	6.0	4.0	161
335	0	22.6	0	0	0	0	0	0	0	1	0	0	0	0	0	0	0	0	0	0	0	0	0	3.9	0	1	2	3.3	14	0.95	6.5	4.2	151
335	0	22.6	0	0	0	0	0	0	0	1	0	0	0	0	0	0	0	0	0	0	0	0	0	3.9	0	1	3	3.5	18	0.86	6.1	4.1	128
336	0	23.9	0	0	1	0	0	0	0	0	1	0	0	1	0	0	0	0	0	0	0	0	0	3.9	0	1	1	3.9	20	0.86	8.0	5.0	202
336	0	23.9	0	0	1	0	0	0	0	0	1	0	0	1	0	0	0	0	0	0	0	0	0	3.9	0	1	2	4.8	28	0.87	7.2	4.3	176
336	0	23.9	0	0	1	0	0	0	0	0	1	0	0	1	0	0	0	0	0	0	0	0	0	3.9	0	1	3	5.1	16	0.88	8.0	4.5	206
337	1	23.0	0	0	0	0	0	0	0	1	0	0	0	0	0	1	1	0	0	0	0	0	1	5.1	0	0	1	5.1	23	0.92	7.0	5.0	226
337	1	23.0	0	0	0	0	0	0	0	1	0	0	0	0	0	1	1	0	0	0	0	0	1	5.1	0	0	2	4.5	16	0.86	6.7	4.1	225
337	1	23.0	0	0	0	0	0	0	0	1	0	0	0	0	0	1	1	0	0	0	0	0	1	5.1	0	0	3	4.7	18	0.81	7.1	4.2	263
338	1	22.1	1	1	1	1	0	0	1	2	1	0	0	0	0	1	1	0	1	0	1	0	0	3.9	0	1	1	3.9	14	1.01	7.0	4.0	177
338	1	22.1	1	1	1	1	0	0	1	2	1	0	0	0	0	1	1	0	1	0	1	0	0	3.9	0	1	2	3.4	17	0.98	7.2	4.5	202
338	1	22.1	1	1	1	1	0	0	1	2	1	0	0	0	0	1	1	0	1	0	1	0	0	3.9	0	1	3	3.7	18	0.95	6.9	4.3	200
339	1	22.8	1	1	0	0	0	0	1	0	0	0	0	0	0	1	1	0	1	0	1	0	0	6.2	0	0	1	6.2	12	1.10	8.0	4.0	170
339	1	22.8	1	1	0	0	0	0	1	0	0	0	0	0	0	1	1	0	1	0	1	0	0	6.2	0	0	2	4.9	20	1.12	7.7	4.6	189
339	1	22.8	1	1	0	0	0	0	1	0	0	0	0	0	0	1	1	0	1	0	1	0	0	6.2	0	0	3	6.2	23	1.10	7.4	4.4	193
340	0	25.7	0	1	0	0	0	0	0	0	0	0	0	0	0	0	0	0	0	0	0	0	0	5.9	0	0	1	5.9	17	1.17	7.0	4.0	174
340	0	25.7	0	1	0	0	0	0	0	0	0	0	0	0	0	0	0	0	0	0	0	0	0	5.9	0	0	2	6.0	24	1.13	7.1	4.0	192
340	0	25.7	0	1	0	0	0	0	0	0	0	0	0	0	0	0	0	0	0	0	0	0	0	5.9	0	0	3	5.5	18	1.02	7.4	4.1	250
341	0	27.5	0	1	0	0	0	0	0	1	0	0	0	0	0	0	0	0	0	0	0	0	0	5.0	0	0	1	5.0	11	1.16	8.0	4.0	236
341	0	27.5	0	1	0	0	0	0	0	1	0	0	0	0	0	0	0	0	0	0	0	0	0	5.0	0	0	2	6.0	14	1.16	7.4	4.2	225
341	0	27.5	0	1	0	0	0	0	0	1	0	0	0	0	0	0	0	0	0	0	0	0	0	5.0	0	0	3	6.2	14	1.06	7.9	4.4	226
342	0	25.0	1	1	1	1	0	0	0	1	1	1	0	1	0	0	0	0	0	0	0	0	0	5.0	0	0	1	5.0	24	0.98	7.0	5.0	202
342	0	25.0	1	1	1	1	0	0	0	1	1	1	0	1	0	0	0	0	0	0	0	0	0	5.0	0	0	2	5.4	24	1.00	7.4	4.6	219
342	0	25.0	1	1	1	1	0	0	0	1	1	1	0	1	0	0	0	0	0	0	0	0	0	5.0	0	0	3	6.4	21	0.93	6.9	4.5	203
343	0	32.5	0	0	1	0	0	0	0	1	1	0	0	1	0	0	0	0	0	0	0	0	0	5.0	0	0	1	5.0	15	0.93	8.0	5.0	170
343	0	32.5	0	0	1	0	0	0	0	1	1	0	0	1	0	0	0	0	0	0	0	0	0	5.0	0	0	2	6.6	16	0.85	7.1	4.1	171
343	0	32.5	0	0	1	0	0	0	0	1	1	0	0	1	0	0	0	0	0	0	0	0	0	5.0	0	0	3	5.9	13	0.64	6.9	4.1	177
344	0	20.2	0	0	0	0	0	0	0	1	0	0	0	0	0	0	0	0	0	0	0	0	0	8.1	1	0	1	8.1	17	1.10	7.0	4.0	162
344	0	20.2	0	0	0	0	0	0	0	1	0	0	0	0	0	0	0	0	0	0	0	0	0	8.1	1	0	2	8.3	19	0.98	6.8	4.2	163
344	0	20.2	0	0	0	0	0	0	0	1	0	0	0	0	0	0	0	0	0	0	0	0	0	8.1	1	0	3	8.0	15	1.04	7.1	4.0	171
345	1	22.2	0	0	1	0	0	0	0	1	0	0	0	0	0	1	1	0	0	0	0	0	1	6.8	0	0	1	6.8	18	0.87	7.0	4.0	162
345	1	22.2	0	0	1	0	0	0	0	1	0	0	0	0	0	1	1	0	0	0	0	0	1	6.8	0	0	2	6.3	19	0.88	7.2	4.5	203
345	1	22.2	0	0	1	0	0	0	0	1	0	0	0	0	0	1	1	0	0	0	0	0	1	6.8	0	0	3	6.5	21	0.93	7.1	4.3	222
346	1	25.2	0	0	0	0	0	0	1	0	0	0	0	0	0	1	1	0	1	1	0	0	1	6.4	0	0	1	6.4	31	0.88	7.0	4.0	192
346	1	25.2	0	0	0	0	0	0	1	0	0	0	0	0	0	1	1	0	1	1	0	0	1	6.4	0	0	2	4.8	21	0.89	7.5	4.4	219
346	1	25.2	0	0	0	0	0	0	1	0	0	0	0	0	0	1	1	0	1	1	0	0	1	6.4	0	0	3	4.6	25	0.90	7.0	4.1	179
347	0	26.6	1	0	1	0	0	0	0	1	0	1	0	1	0	0	0	0	0	0	0	0	0	6.7	0	0	1	6.7	19	0.82	7.0	4.0	138
347	0	26.6	1	0	1	0	0	0	0	1	0	1	0	1	0	0	0	0	0	0	0	0	0	6.7	0	0	2	6.7	19	1.00	6.9	4.0	137
347	0	26.6	1	0	1	0	0	0	0	1	0	1	0	1	0	0	0	0	0	0	0	0	0	6.7	0	0	3	5.2	20	1.07	6.9	4.3	138
348	0	24.4	0	1	0	0	0	1	0	0	0	0	0	0	1	0	0	0	0	0	0	0	0	4.8	0	0	1	4.8	17	1.00	7.0	4.0	171
348	0	24.4	0	1	0	0	0	1	0	0	0	0	0	0	1	0	0	0	0	0	0	0	0	4.8	0	0	2	4.4	19	0.97	6.6	4.1	215
348	0	24.4	0	1	0	0	0	1	0	0	0	0	0	0	1	0	0	0	0	0	0	0	0	4.8	0	0	3	5.0	19	0.97	7.5	4.5	185
349	0	22.7	0	0	0	0	0	0	0	0	0	0	0	0	0	0	0	0	0	0	0	0	0	6.6	0	0	1	6.6	16	0.90	7.0	5.0	171
349	0	22.7	0	0	0	0	0	0	0	0	0	0	0	0	0	0	0	0	0	0	0	0	0	6.6	0	0	2	6.5	12	0.92	7.3	4.7	191
349	0	22.7	0	0	0	0	0	0	0	0	0	0	0	0	0	0	0	0	0	0	0	0	0	6.6	0	0	3	6.6	16	0.95	7.1	4.8	194
350	0	25.7	0	1	0	0	0	0	0	2	1	0	0	1	1	0	0	0	0	0	0	0	0	7.4	1	0	1	7.4	10	0.91	7.0	4.0	193
350	0	25.7	0	1	0	0	0	0	0	2	1	0	0	1	1	0	0	0	0	0	0	0	0	7.4	1	0	2	7.5	12	0.85	6.9	4.2	228
350	0	25.7	0	1	0	0	0	0	0	2	1	0	0	1	1	0	0	0	0	0	0	0	0	7.4	1	0	3	7.1	13	0.82	7.1	4.3	178

id	Group	BMI	Current_ETOH	Ever_smoke	HTN	DM	CAD	DYSLIPID	Stage_4	ECOG	Con_asa	Con_thia	con_loopD	con_ARB	con_statin	Anti_Androgen	Bicalutamide	Cyproterone	GNRH	Leuprorelin	Goserelin	Triptorelin	Conc_RT	Base_UA	Base_hyperUA	Base_hypoUA	time	UA	BUN	CR	PROT	ALB	CHOL
351	1	27.9	0	0	0	0	0	0	0	2	0	0	0	0	0	1	1	0	1	1	0	0	0	5.9	0	0	1	5.9	14	1.00	8.0	4.0	236
351	1	27.9	0	0	0	0	0	0	0	2	0	0	0	0	0	1	1	0	1	1	0	0	0	5.9	0	0	2	4.2	13	1.01	7.9	5.0	257
351	1	27.9	0	0	0	0	0	0	0	2	0	0	0	0	0	1	1	0	1	1	0	0	0	5.9	0	0	3	4.5	13	0.98	7.5	4.9	231
352	0	31.9	1	1	1	0	0	1	0	1	0	0	0	1	1	0	0	0	0	0	0	0	0	7.1	1	0	1	7.1	13	1.05	8.0	4.0	110
352	0	31.9	1	1	1	0	0	1	0	1	0	0	0	1	1	0	0	0	0	0	0	0	0	7.1	1	0	2	7.0	15	0.91	7.6	4.4	99
352	0	31.9	1	1	1	0	0	1	0	1	0	0	0	1	1	0	0	0	0	0	0	0	0	7.1	1	0	3	6.7	14	0.98	7.2	3.8	117
353	1	25.7	1	1	1	0	0	0	0	1	0	0	0	0	0	0	0	0	1	1	0	0	1	6.3	0	0	1	6.3	11	0.98	7.0	5.0	197
353	1	25.7	1	1	1	0	0	0	0	1	0	0	0	0	0	0	0	0	1	1	0	0	1	6.3	0	0	2	5.0	15	0.97	6.4	4.0	178
353	1	25.7	1	1	1	0	0	0	0	1	0	0	0	0	0	0	0	0	1	1	0	0	1	6.3	0	0	3	#NULL!	18	0.93	#NULL!	#NULL!	210
354	0	25.2	0	0	1	0	0	0	0	2	0	1	0	1	0	0	0	0	0	0	0	0	0	5.7	0	0	1	5.7	20	1.05	7.0	4.0	163
354	0	25.2	0	0	1	0	0	0	0	2	0	1	0	1	0	0	0	0	0	0	0	0	0	5.7	0	0	2	5.3	21	1.00	6.5	3.9	167
354	0	25.2	0	0	1	0	0	0	0	2	0	1	0	1	0	0	0	0	0	0	0	0	0	5.7	0	0	3	4.6	15	1.01	6.8	4.1	154
355	1	24.1	1	1	0	1	0	0	1	1	0	0	0	0	0	1	1	0	1	1	0	0	1	6.4	0	0	1	6.4	14	0.80	7.0	4.0	136
355	1	24.1	1	1	0	1	0	0	1	1	0	0	0	0	0	1	1	0	1	1	0	0	1	6.4	0	0	2	7.0	16	0.80	6.9	4.2	156
355	1	24.1	1	1	0	1	0	0	1	1	0	0	0	0	0	1	1	0	1	1	0	0	1	6.4	0	0	3	5.4	16	0.71	7.1	4.3	168
356	0	22.6	1	1	0	0	0	0	0	0	0	0	0	0	0	0	0	0	0	0	0	0	0	7.4	1	0	1	7.4	12	1.08	8.0	4.0	253
356	0	22.6	1	1	0	0	0	0	0	0	0	0	0	0	0	0	0	0	0	0	0	0	0	7.4	1	0	2	7.8	16	1.05	7.0	4.2	243
356	0	22.6	1	1	0	0	0	0	0	0	0	0	0	0	0	0	0	0	0	0	0	0	0	7.4	1	0	3	6.7	13	1.04	6.9	4.2	261
357	1	25.7	1	1	1	0	0	0	1	1	0	0	0	0	0	0	0	0	1	0	1	0	0	5.9	0	0	1	5.9	15	0.89	7.0	4.0	174
357	1	25.7	1	1	1	0	0	0	1	1	0	0	0	0	0	0	0	0	1	0	1	0	0	5.9	0	0	2	6.2	15	0.81	7.1	4.5	206
357	1	25.7	1	1	1	0	0	0	1	1	0	0	0	0	0	0	0	0	1	0	1	0	0	5.9	0	0	3	5.7	14	0.76	7.2	4.5	233
358	0	24.9	1	0	1	0	0	0	0	0	0	1	0	1	0	0	0	0	0	0	0	0	0	5.7	0	0	1	5.7	14	0.82	7.0	4.0	183
358	0	24.9	1	0	1	0	0	0	0	0	0	1	0	1	0	0	0	0	0	0	0	0	0	5.7	0	0	2	6.2	17	0.86	7.0	4.3	200
358	0	24.9	1	0	1	0	0	0	0	0	0	1	0	1	0	0	0	0	0	0	0	0	0	5.7	0	0	3	6.1	15	0.72	6.8	4.3	188
359	0	21.2	1	0	0	0	0	0	0	1	0	0	0	0	0	0	0	0	0	0	0	0	0	4.7	0	0	1	4.7	11	0.99	8.0	5.0	141
359	0	21.2	1	0	0	0	0	0	0	1	0	0	0	0	0	0	0	0	0	0	0	0	0	4.7	0	0	2	4.9	13	0.93	7.1	4.5	178
359	0	21.2	1	0	0	0	0	0	0	1	0	0	0	0	0	0	0	0	0	0	0	0	0	4.7	0	0	3	4.7	15	0.91	7.1	4.4	160
360	0	24.7	0	1	0	0	0	0	0	0	0	0	0	0	0	0	0	0	0	0	0	0	0	7.1	1	0	1	7.1	13	0.93	8.0	5.0	200
360	0	24.7	0	1	0	0	0	0	0	0	0	0	0	0	0	0	0	0	0	0	0	0	0	7.1	1	0	2	7.2	13	0.91	7.6	4.6	222
360	0	24.7	0	1	0	0	0	0	0	0	0	0	0	0	0	0	0	0	0	0	0	0	0	7.1	1	0	3	6.8	18	0.89	6.8	4.2	201
361	0	18.7	1	0	0	1	0	0	0	2	0	0	0	0	0	0	0	0	0	0	0	0	0	5.7	0	0	1	5.7	11	1.20	8.0	5.0	145
361	0	18.7	1	0	0	1	0	0	0	2	0	0	0	0	0	0	0	0	0	0	0	0	0	5.7	0	0	2	5.2	11	1.17	6.9	4.4	188
361	0	18.7	1	0	0	1	0	0	0	2	0	0	0	0	0	0	0	0	0	0	0	0	0	5.7	0	0	3	5.3	10	1.08	7.2	4.5	195
362	1	30.2	1	1	1	0	0	0	0	1	0	0	0	0	0	1	1	0	0	0	0	0	1	5.4	0	0	1	5.4	20	1.01	7.0	4.0	173
362	1	30.2	1	1	1	0	0	0	0	1	0	0	0	0	0	1	1	0	0	0	0	0	1	5.4	0	0	2	4.2	15	0.98	7.2	4.1	158
362	1	30.2	1	1	1	0	0	0	0	1	0	0	0	0	0	1	1	0	0	0	0	0	1	5.4	0	0	3	5.0	16	0.91	7.3	4.4	148
363	0	22.5	0	0	1	0	0	0	0	1	0	0	0	0	0	0	0	0	0	0	0	0	0	5.9	0	0	1	5.9	12	0.90	6.0	4.0	154
363	0	22.5	0	0	1	0	0	0	0	1	0	0	0	0	0	0	0	0	0	0	0	0	0	5.9	0	0	2	5.8	20	0.81	7.0	4.0	175
363	0	22.5	0	0	1	0	0	0	0	1	0	0	0	0	0	0	0	0	0	0	0	0	0	5.9	0	0	3	4.2	13	0.85	6.8	4.0	175
364	0	22.7	1	1	1	0	0	0	0	1	0	0	0	0	0	0	0	0	0	0	0	0	0	6.5	0	0	1	6.5	9	0.80	7.0	4.0	185
364	0	22.7	1	1	1	0	0	0	0	1	0	0	0	0	0	0	0	0	0	0	0	0	0	6.5	0	0	2	8.0	8	0.83	7.3	4.2	285
364	0	22.7	1	1	1	0	0	0	0	1	0	0	0	0	0	0	0	0	0	0	0	0	0	6.5	0	0	3	8.4	10	0.80	7.4	4.4	242
365	0	22.5	1	0	0	1	0	0	0	1	0	0	0	0	0	0	0	0	0	0	0	0	0	4.2	0	0	1	4.2	16	1.01	7.0	4.0	197
365	0	22.5	1	0	0	1	0	0	0	1	0	0	0	0	0	0	0	0	0	0	0	0	0	4.2	0	0	2	5.3	18	0.81	7.3	4.4	215
365	0	22.5	1	0	0	1	0	0	0	1	0	0	0	0	0	0	0	0	0	0	0	0	0	4.2	0	0	3	5.5	15	0.77	6.7	4.0	160
366	0	18.8	1	1	0	0	0	0	0	0	0	0	0	0	0	0	0	0	0	0	0	0	0	5.3	0	0	1	5.3	20	0.97	8.0	4.0	173
366	0	18.8	1	1	0	0	0	0	0	0	0	0	0	0	0	0	0	0	0	0	0	0	0	5.3	0	0	2	5.5	18	0.97	7.6	4.1	178
366	0	18.8	1	1	0	0	0	0	0	0	0	0	0	0	0	0	0	0	0	0	0	0	0	5.3	0	0	3	4.2	14	1.03	7.3	4.1	149
367	0	22.8	1	1	0	0	0	0	0	2	0	0	0	0	0	0	0	0	0	0	0	0	0	5.0	0	0	1	5.0	8	0.85	7.0	4.0	149
367	0	22.8	1	1	0	0	0	0	0	2	0	0	0	0	0	0	0	0	0	0	0	0	0	5.0	0	0	2	5.5	13	0.96	7.4	4.3	184

id	Group	BMI	Current_ETOH	Ever_smoke	HTN	DM	CAD	DYSLIPID	Stage_4	ECOG	Con_asa	Con_thia	con_loopD	con_ARB	con_statin	Anti_Androgen	Bicalutamide	Cyproterone	GNRH	Leuprorelin	Goserelin	Triptorelin	Conc_RT	Base_UA	Base_hyperUA	Base_hypoUA	time	UA	BUN	CR	PROT	ALB	CHOL
367	0	22.8	1	1	0	0	0	0	0	2	0	0	0	0	0	0	0	0	0	0	0	0	0	5.0	0	0	3	4.7	14	1.06	7.3	4.5	182
368	0	15.4	0	0	0	0	0	0	0	2	0	0	0	0	0	0	0	0	0	0	0	0	0	5.8	0	0	1	5.8	14	1.02	7.0	4.0	198
368	0	15.4	0	0	0	0	0	0	0	2	0	0	0	0	0	0	0	0	0	0	0	0	0	5.8	0	0	2	5.6	11	0.97	7.4	4.5	210
368	0	15.4	0	0	0	0	0	0	0	2	0	0	0	0	0	0	0	0	0	0	0	0	0	5.8	0	0	3	5.3	11	0.94	7.7	4.6	225
369	0	23.3	1	1	0	0	0	0	0	1	0	0	0	1	1	0	0	0	0	0	0	0	0	5.1	0	0	1	5.1	14	1.00	8.0	4.0	167
369	0	23.3	1	1	0	0	0	0	0	1	0	0	0	1	1	0	0	0	0	0	0	0	0	5.1	0	0	2	5.6	11	1.04	7.2	4.1	158
369	0	23.3	1	1	0	0	0	0	0	1	0	0	0	1	1	0	0	0	0	0	0	0	0	5.1	0	0	3	6.2	15	0.90	7.5	4.2	172
370	0	25.4	0	0	1	1	0	0	0	1	1	0	0	0	1	0	0	0	0	0	0	0	0	4.7	0	0	1	4.7	13	0.84	7.0	4.0	118
370	0	25.4	0	0	1	1	0	0	0	1	1	0	0	0	1	0	0	0	0	0	0	0	0	4.7	0	0	2	5.2	14	0.89	7.8	4.6	160
370	0	25.4	0	0	1	1	0	0	0	1	1	0	0	0	1	0	0	0	0	0	0	0	0	4.7	0	0	3	5.0	14	0.90	7.8	4.6	167
371	0	21.6	0	1	0	0	0	1	0	0	0	0	0	0	0	0	0	0	0	0	0	0	0	6.1	0	0	1	6.1	21	0.55	7.0	5.0	241
371	0	21.6	0	1	0	0	0	1	0	0	0	0	0	0	0	0	0	0	0	0	0	0	0	6.1	0	0	2	6.1	16	0.43	7.1	4.5	290
371	0	21.6	0	1	0	0	0	1	0	0	0	0	0	0	0	0	0	0	0	0	0	0	0	6.1	0	0	3	5.8	17	0.50	7.2	4.7	273
372	0	29.8	1	1	1	1	0	0	0	1	0	0	0	1	1	0	0	0	0	0	0	0	0	4.7	0	0	1	4.7	11	0.78	6.0	4.0	146
372	0	29.8	1	1	1	1	0	0	0	1	0	0	0	1	1	0	0	0	0	0	0	0	0	4.7	0	0	2	5.4	13	0.82	7.0	4.4	137
372	0	29.8	1	1	1	1	0	0	0	1	0	0	0	1	1	0	0	0	0	0	0	0	0	4.7	0	0	3	5.4	14	0.76	6.8	4.6	167
373	0	25.8	1	1	0	0	0	0	0	2	0	0	0	0	0	0	0	0	0	0	0	0	0	7.5	1	0	1	7.5	15	0.79	6.0	4.0	145
373	0	25.8	1	1	0	0	0	0	0	2	0	0	0	0	0	0	0	0	0	0	0	0	0	7.5	1	0	2	6.1	22	0.88	6.8	4.4	155
373	0	25.8	1	1	0	0	0	0	0	2	0	0	0	0	0	0	0	0	0	0	0	0	0	7.5	1	0	3	6.6	19	0.95	6.6	4.4	176
374	1	26.8	0	0	1	0	0	0	1	2	1	0	0	1	0	1	1	0	1	1	0	0	0	6.2	0	0	1	6.2	17	1.15	8.0	4.0	171
374	1	26.8	0	0	1	0	0	0	1	2	1	0	0	1	0	1	1	0	1	1	0	0	0	6.2	0	0	2	4.8	21	1.09	7.7	4.0	124
374	1	26.8	0	0	1	0	0	0	1	2	1	0	0	1	0	1	1	0	1	1	0	0	0	6.2	0	0	3	4.7	19	0.89	7.8	4.3	140
375	1	22.1	0	0	0	0	0	0	0	2	0	0	0	0	0	1	1	0	1	0	1	0	0	3.1	0	1	1	3.1	11	1.07	6.0	4.0	182
375	1	22.1	0	0	0	0	0	0	0	2	0	0	0	0	0	1	1	0	1	0	1	0	0	3.1	0	1	2	3.8	15	1.04	6.6	4.0	199
375	1	22.1	0	0	0	0	0	0	0	2	0	0	0	0	0	1	1	0	1	0	1	0	0	3.1	0	1	3	3.7	14	0.94	6.9	4.1	207
376	0	26.3	1	1	0	1	0	1	0	2	0	0	0	0	1	0	0	0	0	0	0	0	0	5.8	0	0	1	5.8	14	1.03	7.0	4.0	108
376	0	26.3	1	1	0	1	0	1	0	2	0	0	0	0	1	0	0	0	0	0	0	0	0	5.8	0	0	2	6.9	18	1.02	7.5	4.4	147
376	0	26.3	1	1	0	1	0	1	0	2	0	0	0	0	1	0	0	0	0	0	0	0	0	5.8	0	0	3	5.9	17	0.98	7.6	4.4	144
377	0	26.2	1	0	1	0	0	0	0	2	0	0	0	1	0	0	0	0	0	0	0	0	0	6.6	0	0	1	6.6	15	0.98	6.0	4.0	176
377	0	26.2	1	0	1	0	0	0	0	2	0	0	0	1	0	0	0	0	0	0	0	0	0	6.6	0	0	2	6.1	16	0.99	7.0	4.6	202
377	0	26.2	1	0	1	0	0	0	0	2	0	0	0	1	0	0	0	0	0	0	0	0	0	6.6	0	0	3	6.7	17	0.97	6.8	4.5	216
378	1	28.1	0	1	0	0	0	0	0	0	0	0	0	0	0	1	1	0	0	0	0	0	0	4.9	0	0	1	4.9	20	0.89	7.0	4.0	163
378	1	28.1	0	1	0	0	0	0	0	0	0	0	0	0	0	1	1	0	0	0	0	0	0	4.9	0	0	2	4.7	19	1.03	8.2	4.9	195
378	1	28.1	0	1	0	0	0	0	0	0	0	0	0	0	0	1	1	0	0	0	0	0	0	4.9	0	0	3	4.3	14	0.78	7.4	4.5	178
379	0	24.0	1	1	0	0	0	0	0	0	0	0	0	0	0	0	0	0	0	0	0	0	0	5.8	0	0	1	5.8	17	0.77	7.0	5.0	171
379	0	24.0	1	1	0	0	0	0	0	0	0	0	0	0	0	0	0	0	0	0	0	0	0	5.8	0	0	2	5.7	16	0.75	7.2	4.5	134
379	0	24.0	1	1	0	0	0	0	0	0	0	0	0	0	0	0	0	0	0	0	0	0	0	5.8	0	0	3	6.0	23	0.70	6.6	4.3	143
380	0	26.8	1	0	1	0	0	0	0	1	0	0	0	0	0	0	0	0	0	0	0	0	0	5.1	0	0	1	5.1	18	0.87	8.0	4.0	152
380	0	26.8	1	0	1	0	0	0	0	1	0	0	0	0	0	0	0	0	0	0	0	0	0	5.1	0	0	2	6.4	12	0.90	7.1	4.3	168
380	0	26.8	1	0	1	0	0	0	0	1	0	0	0	0	0	0	0	0	0	0	0	0	0	5.1	0	0	3	5.3	14	0.94	7.2	3.9	154
381	0	25.7	1	1	0	0	0	1	0	1	0	0	0	0	1	0	0	0	0	0	0	0	0	5.9	0	0	1	5.9	9	0.95	8.0	5.0	249
381	0	25.7	1	1	0	0	0	1	0	1	0	0	0	0	1	0	0	0	0	0	0	0	0	5.9	0	0	2	5.7	11	0.94	7.6	4.9	112
381	0	25.7	1	1	0	0	0	1	0	1	0	0	0	0	1	0	0	0	0	0	0	0	0	5.9	0	0	3	6.1	11	0.86	7.3	4.1	116
382	0	26.8	1	1	1	0	0	0	0	1	0	0	0	0	0	0	0	0	0	0	0	0	0	4.8	0	0	1	4.8	14	0.84	7.0	4.0	204
382	0	26.8	1	1	1	0	0	0	0	1	0	0	0	0	0	0	0	0	0	0	0	0	0	4.8	0	0	2	4.6	17	0.79	7.5	4.6	206
382	0	26.8	1	1	1	0	0	0	0	1	0	0	0	0	0	0	0	0	0	0	0	0	0	4.8	0	0	3	4.3	17	0.79	7.3	4.5	192
383	1	22.7	1	1	1	0	0	1	0	1	0	0	0	0	1	1	1	0	0	0	0	0	0	5.5	0	0	1	5.5	14	1.03	8.0	5.0	165
383	1	22.7	1	1	1	0	0	1	0	1	0	0	0	0	1	1	1	0	0	0	0	0	0	5.5	0	0	2	4.9	13	0.94	7.7	4.5	164
383	1	22.7	1	1	1	0	0	1	0	1	0	0	0	0	1	1	1	0	0	0	0	0	0	5.5	0	0	3	5.3	17	0.93	7.4	4.3	147
384	0	26.0	1	1	1	0	0	1	0	2	0	0	0	1	1	0	0	0	0	0	0	0	0	5.9	0	0	1	5.9	12	0.85	8.0	5.0	187

id	Group	BMI	Current_ETOH	Ever_smoke	HTN	DM	CAD	DYSLIPID	Stage_4	ECOG	Con_asa	Con_thia	con_loopD	con_ARB	con_statin	Anti_Androgen	Bicalutamide	Cyproterone	GNRH	Leuprorelin	Goserelin	Triptorelin	Conc_RT	Base_UA	Base_hyperUA	Base_hypoUA	time	UA	BUN	CR	PROT	ALB	CHOL
384	0	26.0	1	1	1	0	0	1	0	2	0	0	0	1	1	0	0	0	0	0	0	0	0	5.9	0	0	2	6.4	11	0.78	7.4	4.6	164
384	0	26.0	1	1	1	0	0	1	0	2	0	0	0	1	1	0	0	0	0	0	0	0	0	5.9	0	0	3	7.2	11	0.78	8.2	4.8	230
385	0	24.8	0	0	0	0	0	0	0	1	0	0	0	0	0	0	0	0	0	0	0	0	0	6.5	0	0	1	6.5	17	0.86	7.0	4.0	215
385	0	24.8	0	0	0	0	0	0	0	1	0	0	0	0	0	0	0	0	0	0	0	0	0	6.5	0	0	2	5.7	20	0.90	6.8	4.3	194
385	0	24.8	0	0	0	0	0	0	0	1	0	0	0	0	0	0	0	0	0	0	0	0	0	6.5	0	0	3	5.8	14	0.97	6.8	3.9	195
386	1	24.6	0	0	1	0	0	0	1	1	0	0	0	0	0	1	1	0	0	0	0	0	1	7.7	1	0	1	7.7	23	0.98	7.0	4.0	146
386	1	24.6	0	0	1	0	0	0	1	1	0	0	0	0	0	1	1	0	0	0	0	0	1	7.7	1	0	2	5.4	21	0.88	6.9	4.1	152
386	1	24.6	0	0	1	0	0	0	1	1	0	0	0	0	0	1	1	0	0	0	0	0	1	7.7	1	0	3	6.3	22	0.87	7.2	4.5	168
387	0	22.7	0	0	0	0	0	0	0	2	0	0	0	0	0	0	0	0	0	0	0	0	0	6.8	0	0	1	6.8	14	0.98	7.0	5.0	177
387	0	22.7	0	0	0	0	0	0	0	2	0	0	0	0	0	0	0	0	0	0	0	0	0	6.8	0	0	2	6.7	15	0.82	6.7	4.4	184
387	0	22.7	0	0	0	0	0	0	0	2	0	0	0	0	0	0	0	0	0	0	0	0	0	6.8	0	0	3	6.6	12	0.76	6.3	4.1	161
388	0	23.0	0	1	0	0	0	0	0	0	0	0	0	0	0	0	0	0	0	0	0	0	0	4.2	0	0	1	4.2	14	0.81	7.0	4.0	168
388	0	23.0	0	1	0	0	0	0	0	0	0	0	0	0	0	0	0	0	0	0	0	0	0	4.2	0	0	2	4.2	14	0.88	7.3	4.4	214
388	0	23.0	0	1	0	0	0	0	0	0	0	0	0	0	0	0	0	0	0	0	0	0	0	4.2	0	0	3	3.9	16	0.73	6.6	4.0	190
389	0	26.4	0	1	0	0	0	0	0	0	0	0	0	0	0	0	0	0	0	0	0	0	0	5.1	0	0	1	5.1	14	0.78	8.0	5.0	247
389	0	26.4	0	1	0	0	0	0	0	0	0	0	0	0	0	0	0	0	0	0	0	0	0	5.1	0	0	2	4.4	10	0.74	6.6	3.9	153
389	0	26.4	0	1	0	0	0	0	0	0	0	0	0	0	0	0	0	0	0	0	0	0	0	5.1	0	0	3	5.6	13	0.67	6.8	4.3	187
390	0	29.8	0	0	1	0	0	0	0	2	0	0	0	0	0	0	0	0	0	0	0	0	0	4.7	0	0	1	4.7	14	0.67	8.0	4.0	244
390	0	29.8	0	0	1	0	0	0	0	2	0	0	0	0	0	0	0	0	0	0	0	0	0	4.7	0	0	2	6.8	14	0.58	7.9	4.3	248
390	0	29.8	0	0	1	0	0	0	0	2	0	0	0	0	0	0	0	0	0	0	0	0	0	4.7	0	0	3	5.5	16	0.58	8.0	4.2	248
391	0	21.5	0	0	1	0	0	0	0	2	1	0	0	1	0	0	0	0	0	0	0	0	0	5.0	0	0	1	5.0	16	0.65	7.0	5.0	155
391	0	21.5	0	0	1	0	0	0	0	2	1	0	0	1	0	0	0	0	0	0	0	0	0	5.0	0	0	2	5.6	16	0.71	7.1	4.4	166
391	0	21.5	0	0	1	0	0	0	0	2	1	0	0	1	0	0	0	0	0	0	0	0	0	5.0	0	0	3	5.5	17	0.61	7.7	4.6	171
392	1	26.7	0	1	0	0	0	0	0	1	0	0	0	0	0	1	1	0	0	0	0	0	1	6.4	0	0	1	6.4	13	1.19	7.0	4.0	189
392	1	26.7	0	1	0	0	0	0	0	1	0	0	0	0	0	1	1	0	0	0	0	0	1	6.4	0	0	2	5.9	10	1.04	7.4	4.5	215
392	1	26.7	0	1	0	0	0	0	0	1	0	0	0	0	0	1	1	0	0	0	0	0	1	6.4	0	0	3	6.0	13	0.99	7.4	4.3	203
393	0	27.2	1	1	1	0	0	0	0	2	0	0	0	0	0	0	0	0	0	0	0	0	0	7.4	1	0	1	7.4	15	1.11	7.0	4.0	138
393	0	27.2	1	1	1	0	0	0	0	2	0	0	0	0	0	0	0	0	0	0	0	0	0	7.4	1	0	2	8.3	14	1.08	6.7	4.2	143
393	0	27.2	1	1	1	0	0	0	0	2	0	0	0	0	0	0	0	0	0	0	0	0	0	7.4	1	0	3	6.2	11	1.04	7.3	4.6	167
394	0	22.1	0	0	0	0	0	0	0	0	0	0	0	0	0	0	0	0	0	0	0	0	0	5.6	0	0	1	5.6	16	0.71	6.0	3.0	119
394	0	22.1	0	0	0	0	0	0	0	0	0	0	0	0	0	0	0	0	0	0	0	0	0	5.6	0	0	2	2.7	14	0.63	7.9	3.5	138
394	0	22.1	0	0	0	0	0	0	0	0	0	0	0	0	0	0	0	0	0	0	0	0	0	5.6	0	0	3	4.7	12	0.61	7.3	4.0	170
395	0	24.7	0	0	0	0	0	1	0	1	0	0	0	0	1	0	0	0	0	0	0	0	0	3.4	0	1	1	3.4	11	0.71	7.0	4.0	170
395	0	24.7	0	0	0	0	0	1	0	1	0	0	0	0	1	0	0	0	0	0	0	0	0	3.4	0	1	2	3.1	10	0.73	7.2	4.0	123
395	0	24.7	0	0	0	0	0	1	0	1	0	0	0	0	1	0	0	0	0	0	0	0	0	3.4	0	1	3	3.2	10	0.76	7.4	3.6	193
396	1	27.6	1	1	1	0	0	1	0	2	0	0	1	1	1	1	1	0	1	1	0	0	0	5.3	0	0	1	5.3	14	0.98	8.0	5.0	136
396	1	27.6	1	1	1	0	0	1	0	2	0	0	1	1	1	1	1	0	1	1	0	0	0	5.3	0	0	2	5.2	12	0.99	7.9	4.8	184
396	1	27.6	1	1	1	0	0	1	0	2	0	0	1	1	1	1	1	0	1	1	0	0	0	5.3	0	0	3	6.8	15	1.03	7.9	4.8	185
397	0	28.2	0	1	1	1	0	1	0	1	1	1	0	1	0	0	0	0	0	0	0	0	0	3.7	0	1	1	3.7	13	0.99	7.0	4.0	146
397	0	28.2	0	1	1	1	0	1	0	1	1	1	0	1	0	0	0	0	0	0	0	0	0	3.7	0	1	2	5.8	17	1.00	7.1	4.2	151
397	0	28.2	0	1	1	1	0	1	0	1	1	1	0	1	0	0	0	0	0	0	0	0	0	3.7	0	1	3	6.8	19	1.08	7.1	4.2	144
398	0	24.9	1	1	0	0	0	0	0	0	0	0	0	0	0	0	0	0	0	0	0	0	0	5.3	0	0	1	5.3	18	0.90	8.0	5.0	265
398	0	24.9	1	1	0	0	0	0	0	0	0	0	0	0	0	0	0	0	0	0	0	0	0	5.3	0	0	2	6.0	17	0.88	7.1	4.3	228
398	0	24.9	1	1	0	0	0	0	0	0	0	0	0	0	0	0	0	0	0	0	0	0	0	5.3	0	0	3	6.4	14	0.78	7.6	4.7	176
399	0	25.8	1	1	0	0	0	0	0	0	0	0	0	0	0	0	0	0	0	0	0	0	0	4.2	0	0	1	4.2	12	0.98	7.0	4.0	146
399	0	25.8	1	1	0	0	0	0	0	0	0	0	0	0	0	0	0	0	0	0	0	0	0	4.2	0	0	2	6.7	11	0.92	7.6	4.5	159
399	0	25.8	1	1	0	0	0	0	0	0	0	0	0	0	0	0	0	0	0	0	0	0	0	4.2	0	0	3	4.2	22	0.91	6.9	4.3	156
400	0	33.1	0	1	1	0	0	1	0	1	0	0	0	1	1	0	0	0	0	0	0	0	0	6.6	0	0	1	6.6	14	0.99	7.0	4.0	171
400	0	33.1	0	1	1	0	0	1	0	1	0	0	0	1	1	0	0	0	0	0	0	0	0	6.6	0	0	2	5.6	14	0.88	7.1	4.4	142
400	0	33.1	0	1	1	0	0	1	0	1	0	0	0	1	1	0	0	0	0	0	0	0	0	6.6	0	0	3	4.6	11	0.96	7.3	4.3	154

id	Group	BMI	Current_ETOH	Ever_smoke	HTN	DM	CAD	DYSLIPID	Stage_4	ECOG	Con_asa	Con_thia	con_loopD	con_ARB	con_statin	Anti_Androgen	Bicalutamide	Cyproterone	GNRH	Leuprorelin	Goserelin	Triptorelin	Conc_RT	Base_UA	Base_hyperUA	Base_hypoUA	time	UA	BUN	CR	PROT	ALB	CHOL
401	0	29.8	1	0	1	0	0	0	0	1	0	1	0	0	0	0	0	0	0	0	0	0	0	5.2	0	0	1	5.2	11	0.80	7.0	4.0	154
401	0	29.8	1	0	1	0	0	0	0	1	0	1	0	0	0	0	0	0	0	0	0	0	0	5.2	0	0	2	6.8	16	0.89	7.6	4.5	158
401	0	29.8	1	0	1	0	0	0	0	1	0	1	0	0	0	0	0	0	0	0	0	0	0	5.2	0	0	3	7.8	18	0.08	7.4	4.6	202
402	0	22.6	1	1	0	1	0	1	0	0	0	0	0	0	1	0	0	0	0	0	0	0	0	4.2	0	0	1	4.2	13	0.94	8.0	5.0	61
402	0	22.6	1	1	0	1	0	1	0	0	0	0	0	0	1	0	0	0	0	0	0	0	0	4.2	0	0	2	4.4	12	0.90	7.8	4.9	135
402	0	22.6	1	1	0	1	0	1	0	0	0	0	0	0	1	0	0	0	0	0	0	0	0	4.2	0	0	3	44.0	18	0.87	7.1	4.4	125
403	1	21.8	0	0	0	0	0	0	1	0	0	0	0	0	0	0	0	0	1	1	0	0	0	6.6	0	0	1	6.6	19	1.05	7.0	4.0	121
403	1	21.8	0	0	0	0	0	0	1	0	0	0	0	0	0	0	0	0	1	1	0	0	0	6.6	0	0	2	4.2	11	1.02	7.0	4.0	112
403	1	21.8	0	0	0	0	0	0	1	0	0	0	0	0	0	0	0	0	1	1	0	0	0	6.6	0	0	3	5.3	21	0.93	7.3	4.1	122
404	0	23.1	1	1	1	0	0	0	0	1	0	0	0	1	0	0	0	0	0	0	0	0	0	6.0	0	0	1	6.0	14	0.86	7.0	5.0	194
404	0	23.1	1	1	1	0	0	0	0	1	0	0	0	1	0	0	0	0	0	0	0	0	0	6.0	0	0	2	6.2	15	0.78	7.0	4.5	192
404	0	23.1	1	1	1	0	0	0	0	1	0	0	0	1	0	0	0	0	0	0	0	0	0	6.0	0	0	3	6.0	18	0.76	6.9	4.4	200
405	0	22.6	1	1	0	0	0	0	0	0	0	0	0	0	0	0	0	0	0	0	0	0	0	4.9	0	0	1	4.9	15	1.04	7.0	4.0	175
405	0	22.6	1	1	0	0	0	0	0	0	0	0	0	0	0	0	0	0	0	0	0	0	0	4.9	0	0	2	5.4	18	1.01	7.3	4.2	196
405	0	22.6	1	1	0	0	0	0	0	0	0	0	0	0	0	0	0	0	0	0	0	0	0	4.9	0	0	3	5.5	23	0.92	6.8	4.0	164
406	0	29.1	1	1	0	0	0	0	0	0	0	0	0	0	0	0	0	0	0	0	0	0	0	8.7	1	0	1	8.7	20	0.74	8.0	5.0	148
406	0	29.1	1	1	0	0	0	0	0	0	0	0	0	0	0	0	0	0	0	0	0	0	0	8.7	1	0	2	7.8	20	0.69	7.1	4.3	192
406	0	29.1	1	1	0	0	0	0	0	0	0	0	0	0	0	0	0	0	0	0	0	0	0	8.7	1	0	3	6.9	18	0.66	7.4	4.5	193
407	1	17.6	0	0	0	0	0	0	0	1	0	0	0	0	0	1	1	0	1	1	0	0	1	5.6	0	0	1	5.6	15	1.15	7.0	4.0	167
407	1	17.6	0	0	0	0	0	0	0	1	0	0	0	0	0	1	1	0	1	1	0	0	1	5.6	0	0	2	5.3	15	1.08	7.0	4.1	219
407	1	17.6	0	0	0	0	0	0	0	1	0	0	0	0	0	1	1	0	1	1	0	0	1	5.6	0	0	3	4.9	14	1.15	7.0	4.3	234
408	1	24.1	0	0	0	0	0	0	1	1	0	0	0	0	0	0	0	0	1	0	0	1	1	2.7	0	1	1	2.7	16	0.69	7.0	4.0	174
408	1	24.1	0	0	0	0	0	0	1	1	0	0	0	0	0	0	0	0	1	0	0	1	1	2.7	0	1	2	2.5	18	0.72	6.6	4.1	172
408	1	24.1	0	0	0	0	0	0	1	1	0	0	0	0	0	0	0	0	1	0	0	1	1	2.7	0	1	3	2.8	20	0.67	6.8	4.2	246
409	0	23.7	1	1	0	0	0	0	0	0	0	0	0	0	0	0	0	0	0	0	0	0	0	5.3	0	0	1	5.3	15	0.80	8.0	4.0	159
409	0	23.7	1	1	0	0	0	0	0	0	0	0	0	0	0	0	0	0	0	0	0	0	0	5.3	0	0	2	4.4	16	0.75	7.0	4.3	158
409	0	23.7	1	1	0	0	0	0	0	0	0	0	0	0	0	0	0	0	0	0	0	0	0	5.3	0	0	3	4.6	16	0.74	7.2	4.4	166
410	0	25.0	1	1	1	0	0	1	0	1	0	1	0	0	1	0	0	0	0	0	0	0	0	5.1	0	0	1	5.1	18	0.77	7.0	4.0	220
410	0	25.0	1	1	1	0	0	1	0	1	0	1	0	0	1	0	0	0	0	0	0	0	0	5.1	0	0	2	4.1	17	0.75	6.6	4.1	145
410	0	25.0	1	1	1	0	0	1	0	1	0	1	0	0	1	0	0	0	0	0	0	0	0	5.1	0	0	3	4.1	16	0.74	7.2	4.4	158
411	0	29.4	1	1	1	0	0	0	0	1	1	0	0	0	0	0	0	0	0	0	0	0	0	9.7	1	0	1	9.7	23	0.99	7.0	5.0	181
411	0	29.4	1	1	1	0	0	0	0	1	1	0	0	0	0	0	0	0	0	0	0	0	0	9.7	1	0	2	7.2	15	1.10	6.9	4.6	196
411	0	29.4	1	1	1	0	0	0	0	1	1	0	0	0	0	0	0	0	0	0	0	0	0	9.7	1	0	3	8.9	16	0.98	6.9	4.6	205
412	0	28.9	0	1	1	0	0	0	0	1	0	0	0	1	0	0	0	0	0	0	0	0	0	6.1	0	0	1	6.1	17	0.80	7.0	4.0	170
412	0	28.9	0	1	1	0	0	0	0	1	0	0	0	1	0	0	0	0	0	0	0	0	0	6.1	0	0	2	6.0	22	0.80	7.2	4.1	201
412	0	28.9	0	1	1	0	0	0	0	1	0	0	0	1	0	0	0	0	0	0	0	0	0	6.1	0	0	3	4.5	14	0.62	7.3	4.1	178
413	1	27.2	0	1	1	0	0	0	0	1	0	0	0	1	0	0	0	0	1	1	0	0	0	6.0	0	0	1	6.0	16	0.99	7.0	4.0	202
413	1	27.2	0	1	1	0	0	0	0	1	0	0	0	1	0	0	0	0	1	1	0	0	0	6.0	0	0	2	5.1	14	0.92	6.8	4.2	210
413	1	27.2	0	1	1	0	0	0	0	1	0	0	0	1	0	0	0	0	1	1	0	0	0	6.0	0	0	3	5.4	16	0.93	6.7	4.2	192
414	0	27.0	1	1	0	0	0	0	0	0	0	0	0	0	0	0	0	0	0	0	0	0	0	5.9	0	0	1	5.9	11	0.79	7.0	4.0	181
414	0	27.0	1	1	0	0	0	0	0	0	0	0	0	0	0	0	0	0	0	0	0	0	0	5.9	0	0	2	6.2	12	0.69	7.1	4.4	161
414	0	27.0	1	1	0	0	0	0	0	0	0	0	0	0	0	0	0	0	0	0	0	0	0	5.9	0	0	3	6.4	12	0.71	7.2	4.6	170
415	0	19.1	1	0	1	0	0	0	0	0	0	0	0	0	0	0	0	0	0	0	0	0	0	3.7	0	1	1	3.7	18	1.12	7.0	4.0	165
415	0	19.1	1	0	1	0	0	0	0	0	0	0	0	0	0	0	0	0	0	0	0	0	0	3.7	0	1	2	4.0	16	1.07	6.6	4.4	170
415	0	19.1	1	0	1	0	0	0	0	0	0	0	0	0	0	0	0	0	0	0	0	0	0	3.7	0	1	3	5.7	14	1.06	6.9	4.5	191
416	1	23.4	1	1	0	0	0	0	0	1	0	0	0	0	0	1	1	0	0	0	0	0	1	7.0	1	0	1	7.0	16	0.98	7.0	4.0	179
416	1	23.4	1	1	0	0	0	0	0	1	0	0	0	0	0	1	1	0	0	0	0	0	1	7.0	1	0	2	5.7	18	0.79	7.6	4.5	180
416	1	23.4	1	1	0	0	0	0	0	1	0	0	0	0	0	1	1	0	0	0	0	0	1	7.0	1	0	3	5.7	17	0.83	7.5	4.4	162
417	0	24.5	0	1	0	0	0	0	0	1	0	0	0	0	0	0	0	0	0	0	0	0	0	6.7	0	0	1	6.7	14	0.81	7.0	4.0	227
417	0	24.5	0	1	0	0	0	0	0	1	0	0	0	0	0	0	0	0	0	0	0	0	0	6.7	0	0	2	6.5	13	0.89	7.1	4.4	205

id	Group	BMI	Current_ETOH	Ever_smoke	HTN	DM	CAD	DYSLIPID	Stage_4	ECOG	Con_asa	Con_thia	con_loopD	con_ARB	con_statin	Anti_Androgen	Bicalutamide	Cyproterone	GNRH	Leuprorelin	Goserelin	Triptorelin	Conc_RT	Base_UA	Base_hyperUA	Base_hypoUA	time	UA	BUN	CR	PROT	ALB	CHOL
417	0	24.5	0	1	0	0	0	0	0	1	0	0	0	0	0	0	0	0	0	0	0	0	0	6.7	0	0	3	5.5	14	0.80	6.9	4.4	195
418	0	21.0	1	1	0	0	0	0	0	0	0	0	0	0	1	0	0	0	0	0	0	0	0	6.3	0	0	1	6.3	17	0.74	7.0	4.0	182
418	0	21.0	1	1	0	0	0	0	0	0	0	0	0	0	1	0	0	0	0	0	0	0	0	6.3	0	0	2	7.3	16	0.76	7.1	4.5	211
418	0	21.0	1	1	0	0	0	0	0	0	0	0	0	0	1	0	0	0	0	0	0	0	0	6.3	0	0	3	8.7	12	0.88	7.3	4.5	218
419	0	25.8	0	1	1	1	0	1	0	1	1	0	0	1	1	0	0	0	0	0	0	0	0	4.3	0	0	1	4.3	14	1.07	7.0	4.0	134
419	0	25.8	0	1	1	1	0	1	0	1	1	0	0	1	1	0	0	0	0	0	0	0	0	4.3	0	0	2	4.1	13	0.99	7.1	4.1	105
419	0	25.8	0	1	1	1	0	1	0	1	1	0	0	1	1	0	0	0	0	0	0	0	0	4.3	0	0	3	4.6	15	0.95	7.6	4.2	128
420	0	25.5	0	1	1	0	0	0	0	2	0	0	0	1	0	0	0	0	0	0	0	0	0	5.2	0	0	1	5.2	11	0.78	8.0	5.0	163
420	0	25.5	0	1	1	0	0	0	0	2	0	0	0	1	0	0	0	0	0	0	0	0	0	5.2	0	0	2	4.7	15	0.79	7.8	4.7	170
420	0	25.5	0	1	1	0	0	0	0	2	0	0	0	1	0	0	0	0	0	0	0	0	0	5.2	0	0	3	5.7	13	0.81	7.8	4.6	151
421	0	20.9	0	1	1	0	0	0	0	2	0	0	0	1	0	0	0	0	0	0	0	0	0	4.9	0	0	1	4.9	18	0.89	7.0	4.0	175
421	0	20.9	0	1	1	0	0	0	0	2	0	0	0	1	0	0	0	0	0	0	0	0	0	4.9	0	0	2	4.5	13	0.83	6.8	4.2	200
421	0	20.9	0	1	1	0	0	0	0	2	0	0	0	1	0	0	0	0	0	0	0	0	0	4.9	0	0	3	6.1	22	0.83	7.3	4.4	234
422	0	27.3	0	1	1	0	0	1	0	2	0	0	0	0	1	0	0	0	0	0	0	0	0	8.5	1	0	1	8.5	14	0.80	8.0	4.0	157
422	0	27.3	0	1	1	0	0	1	0	2	0	0	0	0	1	0	0	0	0	0	0	0	0	8.5	1	0	2	7.2	19	0.75	7.6	4.0	146
422	0	27.3	0	1	1	0	0	1	0	2	0	0	0	0	1	0	0	0	0	0	0	0	0	8.5	1	0	3	7.8	25	0.71	7.3	4.1	133
423	0	22.0	0	1	0	0	0	0	0	0	0	0	0	0	0	0	0	0	0	0	0	0	0	5.0	0	0	1	5.0	12	0.67	7.0	4.0	179
423	0	22.0	0	1	0	0	0	0	0	0	0	0	0	0	0	0	0	0	0	0	0	0	0	5.0	0	0	2	6.2	15	0.72	7.4	4.4	216
423	0	22.0	0	1	0	0	0	0	0	0	0	0	0	0	0	0	0	0	0	0	0	0	0	5.0	0	0	3	6.0	12	0.67	7.5	4.4	210
424	0	21.9	1	1	1	1	1	1	0	2	1	0	0	0	1	0	0	0	0	0	0	0	0	5.5	0	0	1	5.5	8	0.88	7.0	4.0	108
424	0	21.9	1	1	1	1	1	1	0	2	1	0	0	0	1	0	0	0	0	0	0	0	0	5.5	0	0	2	6.8	10	0.83	7.0	3.8	108
424	0	21.9	1	1	1	1	1	1	0	2	1	0	0	0	1	0	0	0	0	0	0	0	0	5.5	0	0	3	6.8	13	0.76	7.3	3.9	106
425	1	26.0	0	#NULL!	1	0	1	1	0	2	1	0	0	0	1	0	0	0	1	0	1	0	0	6.1	0	0	1	6.1	19	0.97	7.0	5.0	208
425	1	26.0	0	#NULL!	1	0	1	1	0	2	1	0	0	0	1	0	0	0	1	0	1	0	0	6.1	0	0	2	5.6	20	1.05	6.8	4.3	204
425	1	26.0	0	#NULL!	1	0	1	1	0	2	1	0	0	0	1	0	0	0	1	0	1	0	0	6.1	0	0	3	5.5	18	0.93	6.7	4.2	184
426	0	24.2	1	1	1	0	1	1	0	2	1	0	0	1	1	0	0	0	0	0	0	0	0	7.3	1	0	1	7.3	12	0.86	7.0	5.0	168
426	0	24.2	1	1	1	0	1	1	0	2	1	0	0	1	1	0	0	0	0	0	0	0	0	7.3	1	0	2	5.1	17	0.98	6.5	4.3	133
426	0	24.2	1	1	1	0	1	1	0	2	1	0	0	1	1	0	0	0	0	0	0	0	0	7.3	1	0	3	5.5	8	0.81	6.8	4.5	151
427	0	26.6	0	1	0	1	1	1	0	2	1	0	0	0	1	0	0	0	0	0	0	0	0	6.4	0	0	1	6.4	16	0.78	7.0	4.0	137
427	0	26.6	0	1	0	1	1	1	0	2	1	0	0	0	1	0	0	0	0	0	0	0	0	6.4	0	0	2	6.7	13	0.65	7.2	4.4	134
427	0	26.6	0	1	0	1	1	1	0	2	1	0	0	0	1	0	0	0	0	0	0	0	0	6.4	0	0	3	6.7	18	0.64	7.5	4.6	165
428	0	15.9	0	0	1	0	1	0	0	2	0	0	0	0	0	0	0	0	0	0	0	0	0	4.0	0	0	1	4.0	7	1.07	7.0	4.0	130
428	0	15.9	0	0	1	0	1	0	0	2	0	0	0	0	0	0	0	0	0	0	0	0	0	4.0	0	0	2	3.3	8	1.02	6.5	3.6	142
428	0	15.9	0	0	1	0	1	0	0	2	0	0	0	0	0	0	0	0	0	0	0	0	0	4.0	0	0	3	3.9	11	0.91	7.0	3.8	185
429	0	25.7	1	0	1	0	1	0	0	2	1	1	0	1	0	0	0	0	0	0	0	0	0	4.6	0	0	1	4.6	18	#NULL!	7.0	5.0	173
429	0	25.7	1	0	1	0	1	0	0	2	1	1	0	1	0	0	0	0	0	0	0	0	0	4.6	0	0	2	6.4	22	1.05	7.0	4.5	139
429	0	25.7	1	0	1	0	1	0	0	2	1	1	0	1	0	0	0	0	0	0	0	0	0	4.6	0	0	3	6.5	18	1.01	7.3	4.7	155
430	0	25.1	0	0	0	1	1	1	0	1	0	0	0	0	1	0	0	0	0	0	0	0	0	6.5	0	0	1	6.5	27	1.05	7.0	4.0	135
430	0	25.1	0	0	0	1	1	1	0	1	0	0	0	0	1	0	0	0	0	0	0	0	0	6.5	0	0	2	5.7	19	1.00	6.8	4.4	129
430	0	25.1	0	0	0	1	1	1	0	1	0	0	0	0	1	0	0	0	0	0	0	0	0	6.5	0	0	3	6.6	25	0.99	6.9	4.5	157
431	0	27.6	0	1	1	1	1	1	0	2	1	0	0	1	0	0	0	0	0	0	0	0	0	5.9	0	0	1	5.9	16	1.03	7.0	4.0	140
431	0	27.6	0	1	1	1	1	1	0	2	1	0	0	1	0	0	0	0	0	0	0	0	0	5.9	0	0	2	3.8	17	0.92	7.0	4.0	141
431	0	27.6	0	1	1	1	1	1	0	2	1	0	0	1	0	0	0	0	0	0	0	0	0	5.9	0	0	3	4.0	14	0.90	6.2	4.0	135
432	0	23.5	1	0	0	0	1	0	0	2	1	0	0	0	0	0	0	0	0	0	0	0	0	5.4	0	0	1	5.4	15	1.14	8.0	4.0	141
432	0	23.5	1	0	0	0	1	0	0	2	1	0	0	0	0	0	0	0	0	0	0	0	0	5.4	0	0	2	5.4	12	1.03	8.1	3.5	163
432	0	23.5	1	0	0	0	1	0	0	2	1	0	0	0	0	0	0	0	0	0	0	0	0	5.4	0	0	3	4.1	16	0.95	8.6	3.6	175
433	0	22.7	0	0	1	1	1	1	0	2	1	0	0	1	1	0	0	0	0	0	0	0	0	3.4	0	1	1	3.4	18	0.92	8.0	4.0	174
433	0	22.7	0	0	1	1	1	1	0	2	1	0	0	1	1	0	0	0	0	0	0	0	0	3.4	0	1	2	4.2	18	0.90	7.5	4.2	163
433	0	22.7	0	0	1	1	1	1	0	2	1	0	0	1	1	0	0	0	0	0	0	0	0	3.4	0	1	3	4.5	18	0.82	7.3	4.3	175
434	0	23.7	0	1	1	0	1	1	0	2	1	1	0	1	1	0	0	0	0	0	0	0	0	5.4	0	0	1	5.4	21	0.89	7.0	4.0	147

id	Group	BMI	Current_ETOH	Ever_smoke	HTN	DM	CAD	DYSLIPID	Stage_4	ECOG	Con_asa	Con_thia	con_loopD	con_ARB	con_statin	Anti_Androgen	Bicalutamide	Cyproterone	GNRH	Leuprorelin	Goserelin	Triptorelin	Conc_RT	Base_UA	Base_hyperUA	Base_hypoUA	time	UA	BUN	CR	PROT	ALB	CHOL
434	0	23.7	0	1	1	0	1	1	0	2	1	1	0	1	1	0	0	0	0	0	0	0	0	5.4	0	0	2	5.1	25	0.93	7.2	4.2	158
434	0	23.7	0	1	1	0	1	1	0	2	1	1	0	1	1	0	0	0	0	0	0	0	0	5.4	0	0	3	5.9	21	1.05	6.7	3.9	150
435	1	20.3	0	1	1	1	1	1	1	1	1	0	0	1	1	1	1	0	0	1	0	0	0	6.6	0	0	1	6.6	33	1.12	7.0	4.0	136
435	1	20.3	0	1	1	1	1	1	1	1	1	0	0	1	1	1	1	0	0	1	0	0	0	6.6	0	0	2	6.1	36	1.01	7.5	4.6	104
435	1	20.3	0	1	1	1	1	1	1	1	1	0	0	1	1	1	1	0	0	1	0	0	0	6.6	0	0	3	6.4	35	0.97	7.5	4.6	116
436	0	23.7	1	0	1	0	1	1	0	2	1	0	0	1	1	0	0	0	0	0	0	0	0	5.2	0	0	1	5.2	16	0.73	8.0	5.0	154
436	0	23.7	1	0	1	0	1	1	0	2	1	0	0	1	1	0	0	0	0	0	0	0	0	5.2	0	0	2	4.9	12	0.78	7.5	4.5	140
436	0	23.7	1	0	1	0	1	1	0	2	1	0	0	1	1	0	0	0	0	0	0	0	0	5.2	0	0	3	5.9	14	0.70	7.1	4.5	166
437	0	27.3	0	0	0	1	1	1	0	2	0	0	0	0	1	0	0	0	0	0	0	0	0	3.4	0	1	1	3.4	10	0.92	7.0	5.0	132
437	0	27.3	0	0	0	1	1	1	0	2	0	0	0	0	1	0	0	0	0	0	0	0	0	3.4	0	1	2	5.8	14	0.96	7.0	4.7	150
437	0	27.3	0	0	0	1	1	1	0	2	0	0	0	0	1	0	0	0	0	0	0	0	0	3.4	0	1	3	5.1	15	0.89	6.6	4.3	137
438	0	22.8	0	0	1	0	1	1	0	1	1	0	0	1	1	0	0	0	0	0	0	0	0	4.0	0	0	1	4.0	21	1.17	7.0	4.0	146
438	0	22.8	0	0	1	0	1	1	0	1	1	0	0	1	1	0	0	0	0	0	0	0	0	4.0	0	0	2	4.3	19	1.14	7.3	4.4	156
438	0	22.8	0	0	1	0	1	1	0	1	1	0	0	1	1	0	0	0	0	0	0	0	0	4.0	0	0	3	4.3	21	1.12	7.2	4.4	149
439	0	26.0	1	1	1	0	1	1	0	1	1	0	0	0	1	0	0	0	0	0	0	0	0	6.0	0	0	1	6.0	13	0.81	7.0	4.0	194
439	0	26.0	1	1	1	0	1	1	0	1	1	0	0	0	1	0	0	0	0	0	0	0	0	6.0	0	0	2	5.6	16	0.69	6.9	4.4	210
439	0	26.0	1	1	1	0	1	1	0	1	1	0	0	0	1	0	0	0	0	0	0	0	0	6.0	0	0	3	5.5	17	0.73	6.3	3.9	183
440	1	22.9	1	0	1	0	1	1	0	1	0	0	0	1	1	1	1	0	1	0	1	0	0	4.7	0	0	1	4.7	16	1.06	7.0	4.0	151
440	1	22.9	1	0	1	0	1	1	0	1	0	0	0	1	1	1	1	0	1	0	1	0	0	4.7	0	0	2	4.5	15	1.08	7.0	4.5	149
440	1	22.9	1	0	1	0	1	1	0	1	0	0	0	1	1	1	1	0	1	0	1	0	0	4.7	0	0	3	4.6	19	0.98	6.6	4.3	145
441	0	24.1	1	1	1	1	1	1	0	1	1	0	0	1	1	0	0	0	0	0	0	0	0	4.2	0	0	1	4.2	11	1.00	8.0	4.0	119
441	0	24.1	1	1	1	1	1	1	0	1	1	0	0	1	1	0	0	0	0	0	0	0	0	4.2	0	0	2	5.0	16	0.93	7.5	4.6	140
441	0	24.1	1	1	1	1	1	1	0	1	1	0	0	1	1	0	0	0	0	0	0	0	0	4.2	0	0	3	4.6	15	0.90	7.3	4.4	138
442	0	25.6	0	1	1	1	1	1	0	0	1	0	0	1	1	0	0	0	0	0	0	0	0	5.9	0	0	1	5.9	13	0.76	7.0	4.0	153
442	0	25.6	0	1	1	1	1	1	0	0	1	0	0	1	1	0	0	0	0	0	0	0	0	5.9	0	0	2	5.9	13	0.67	7.2	4.3	147
442	0	25.6	0	1	1	1	1	1	0	0	1	0	0	1	1	0	0	0	0	0	0	0	0	5.9	0	0	3	6.1	13	0.67	6.8	4.2	161
443	0	24.2	1	1	1	0	1	1	0	2	1	0	0	1	1	0	0	0	0	0	0	0	0	8.7	1	0	1	8.7	25	0.74	7.0	4.0	121
443	0	24.2	1	1	1	0	1	1	0	2	1	0	0	1	1	0	0	0	0	0	0	0	0	8.7	1	0	2	7.3	19	0.61	7.2	4.3	137
443	0	24.2	1	1	1	0	1	1	0	2	1	0	0	1	1	0	0	0	0	0	0	0	0	8.7	1	0	3	10.0	24	0.62	7.9	4.6	119
444	1	27.1	0	0	1	0	1	0	1	1	1	0	0	1	0	1	1	0	1	0	1	0	0	4.0	0	0	1	4.0	14	0.94	8.0	4.0	164
444	1	27.1	0	0	1	0	1	0	1	1	1	0	0	1	0	1	1	0	1	0	1	0	0	4.0	0	0	2	3.1	19	0.90	7.4	4.5	187
444	1	27.1	0	0	1	0	1	0	1	1	1	0	0	1	0	1	1	0	1	0	1	0	0	4.0	0	0	3	3.5	14	0.87	7.7	4.6	171
445	0	25.8	0	0	1	1	1	1	0	1	1	0	0	1	1	0	0	0	0	0	0	0	0	5.2	0	0	1	5.2	20	0.86	7.0	4.0	200
445	0	25.8	0	0	1	1	1	1	0	1	1	0	0	1	1	0	0	0	0	0	0	0	0	5.2	0	0	2	4.4	22	0.86	6.8	4.2	159
445	0	25.8	0	0	1	1	1	1	0	1	1	0	0	1	1	0	0	0	0	0	0	0	0	5.2	0	0	3	4.4	22	0.84	6.8	4.2	159
446	0	20.2	0	0	1	0	1	0	0	2	1	0	0	1	0	0	0	0	0	0	0	0	0	4.2	0	0	1	4.2	15	1.08	7.0	4.0	190
446	0	20.2	0	0	1	0	1	0	0	2	1	0	0	1	0	0	0	0	0	0	0	0	0	4.2	0	0	2	4.6	16	1.10	7.1	4.1	185
446	0	20.2	0	0	1	0	1	0	0	2	1	0	0	1	0	0	0	0	0	0	0	0	0	4.2	0	0	3	4.2	18	1.18	7.2	4.3	221
447	0	26.3	0	0	1	0	1	1	0	2	1	0	0	1	1	0	0	0	0	0	0	0	0	5.4	0	0	1	5.4	13	0.96	7.0	4.0	168
447	0	26.3	0	0	1	0	1	1	0	2	1	0	0	1	1	0	0	0	0	0	0	0	0	5.4	0	0	2	4.7	15	1.05	7.1	4.3	158
447	0	26.3	0	0	1	0	1	1	0	2	1	0	0	1	1	0	0	0	0	0	0	0	0	5.4	0	0	3	4.9	16	0.93	7.0	4.2	128
448	0	29.7	1	1	1	0	1	0	0	2	1	1	0	1	0	0	0	0	0	0	0	0	0	6.7	0	0	1	6.7	17	0.65	7.0	4.0	160
448	0	29.7	1	1	1	0	1	0	0	2	1	1	0	1	0	0	0	0	0	0	0	0	0	6.7	0	0	2	6.8	14	0.66	7.2	4.3	166
448	0	29.7	1	1	1	0	1	0	0	2	1	1	0	1	0	0	0	0	0	0	0	0	0	6.7	0	0	3	6.8	15	0.82	7.2	4.7	172
449	0	25.2	1	1	1	1	1	1	0	0	1	0	0	1	1	0	0	0	0	0	0	0	0	7.8	1	0	1	7.8	20	0.97	7.0	4.0	166
449	0	25.2	1	1	1	1	1	1	0	0	1	0	0	1	1	0	0	0	0	0	0	0	0	7.8	1	0	2	7.5	14	0.94	7.5	4.0	137
449	0	25.2	1	1	1	1	1	1	0	0	1	0	0	1	1	0	0	0	0	0	0	0	0	7.8	1	0	3	8.0	17	0.87	7.2	4.0	183
450	1	23.8	0	1	1	1	1	0	1	1	1	0	0	1	1	1	1	0	0	0	0	0	1	5.4	0	0	1	5.4	18	0.78	7.0	4.0	98
450	1	23.8	0	1	1	1	1	0	1	1	1	0	0	1	1	1	1	0	0	0	0	0	1	5.4	0	0	2	4.5	17	0.73	6.7	4.1	106
450	1	23.8	0	1	1	1	1	0	1	1	1	0	0	1	1	1	1	0	0	0	0	0	1	5.4	0	0	3	4.6	23	0.72	7.2	4.3	135

id	Group	BMI	Current_ETOH	Ever_smoke	HTN	DM	CAD	DYSLIPID	Stage_4	ECOG	Con_asa	Con_thia	con_loopD	con_ARB	con_statin	Anti_Androgen	Bicalutamide	Cyproterone	GNRH	Leuprorelin	Goserelin	Triptorelin	Conc_RT	Base_UA	Base_hyperUA	Base_hypoUA	time	UA	BUN	CR	PROT	ALB	CHOL
451	0	22.9	1	1	1	0	1	0	0	1	1	1	0	0	1	0	0	0	0	0	0	0	0	6.9	0	0	1	6.9	18	1.15	7.0	4.0	235
451	0	22.9	1	1	1	0	1	0	0	1	1	1	0	0	1	0	0	0	0	0	0	0	0	6.9	0	0	2	7.2	16	1.03	7.0	4.4	133
451	0	22.9	1	1	1	0	1	0	0	1	1	1	0	0	1	0	0	0	0	0	0	0	0	6.9	0	0	3	7.2	19	1.00	7.2	4.3	141
452	0	25.9	0	0	1	0	1	1	0	2	1	0	0	0	1	0	0	0	0	0	0	0	0	5.7	0	0	1	5.7	20	0.91	8.0	4.0	138
452	0	25.9	0	0	1	0	1	1	0	2	1	0	0	0	1	0	0	0	0	0	0	0	0	5.7	0	0	2	6.6	16	0.84	6.9	3.8	132
452	0	25.9	0	0	1	0	1	1	0	2	1	0	0	0	1	0	0	0	0	0	0	0	0	5.7	0	0	3	6.0	17	0.80	7.8	4.5	155
453	0	24.7	0	0	0	0	1	1	0	2	1	0	0	0	1	0	0	0	0	0	0	0	0	6.0	0	0	1	6.0	16	0.74	7.0	5.0	203
453	0	24.7	0	0	0	0	1	1	0	2	1	0	0	0	1	0	0	0	0	0	0	0	0	6.0	0	0	2	5.9	20	0.90	7.2	4.7	125
453	0	24.7	0	0	0	0	1	1	0	2	1	0	0	0	1	0	0	0	0	0	0	0	0	6.0	0	0	3	5.7	24	0.90	6.8	4.4	209
454	0	31.1	0	1	1	0	1	1	0	2	0	0	1	1	1	0	0	0	0	0	0	0	0	7.1	1	0	1	7.1	13	0.99	7.0	4.0	143
454	0	31.1	0	1	1	0	1	1	0	2	0	0	1	1	1	0	0	0	0	0	0	0	0	7.1	1	0	2	5.8	11	0.96	7.5	4.3	148
454	0	31.1	0	1	1	0	1	1	0	2	0	0	1	1	1	0	0	0	0	0	0	0	0	7.1	1	0	3	6.8	11	0.81	7.5	4.5	141
455	0	26.7	0	0	1	1	1	0	0	1	1	0	0	1	0	0	0	0	0	0	0	0	0	4.4	0	0	1	4.4	20	0.93	8.0	4.0	101
455	0	26.7	0	0	1	1	1	0	0	1	1	0	0	1	0	0	0	0	0	0	0	0	0	4.4	0	0	2	7.1	19	1.03	8.0	4.8	124
455	0	26.7	0	0	1	1	1	0	0	1	1	0	0	1	0	0	0	0	0	0	0	0	0	4.4	0	0	3	6.6	17	1.08	7.8	4.7	104
456	0	26.0	1	0	0	1	1	1	0	1	1	0	0	0	1	0	0	0	0	0	0	0	0	6.3	0	0	1	6.3	17	1.00	7.0	4.0	140
456	0	26.0	1	0	0	1	1	1	0	1	1	0	0	0	1	0	0	0	0	0	0	0	0	6.3	0	0	2	7.0	13	0.98	6.9	4.4	124
456	0	26.0	1	0	0	1	1	1	0	1	1	0	0	0	1	0	0	0	0	0	0	0	0	6.3	0	0	3	7.2	14	0.96	6.8	4.3	134
457	0	23.8	0	0	1	0	1	1	0	1	0	0	0	0	1	0	0	0	0	0	0	0	0	5.1	0	0	1	5.1	14	1.07	8.0	5.0	130
457	0	23.8	0	0	1	0	1	1	0	1	0	0	0	0	1	0	0	0	0	0	0	0	0	5.1	0	0	2	4.7	14	0.99	7.3	4.6	128
457	0	23.8	0	0	1	0	1	1	0	1	0	0	0	0	1	0	0	0	0	0	0	0	0	5.1	0	0	3	3.5	11	0.95	7.6	4.8	139
458	1	25.5	0	1	1	0	1	1	0	1	1	0	0	0	1	1	1	0	0	0	0	0	1	6.4	0	0	1	6.4	15	0.90	7.0	4.0	130
458	1	25.5	0	1	1	0	1	1	0	1	1	0	0	0	1	1	1	0	0	0	0	0	1	6.4	0	0	2	6.0	18	0.78	7.1	4.3	163
458	1	25.5	0	1	1	0	1	1	0	1	1	0	0	0	1	1	1	0	0	0	0	0	1	6.4	0	0	3	5.0	20	0.82	6.9	4.3	171
