# Supplementary figures and images for: Influence of androgen deprivation therapy on serum urate levels in patients with prostate cancer: A retrospective observational study
Source: PLoS One. 2018 Dec 17;13(12):e0209049. doi: 10.1371/journal.pone.0209049 (PMC6296534; doi:10.1371/journal.pone.0209049)

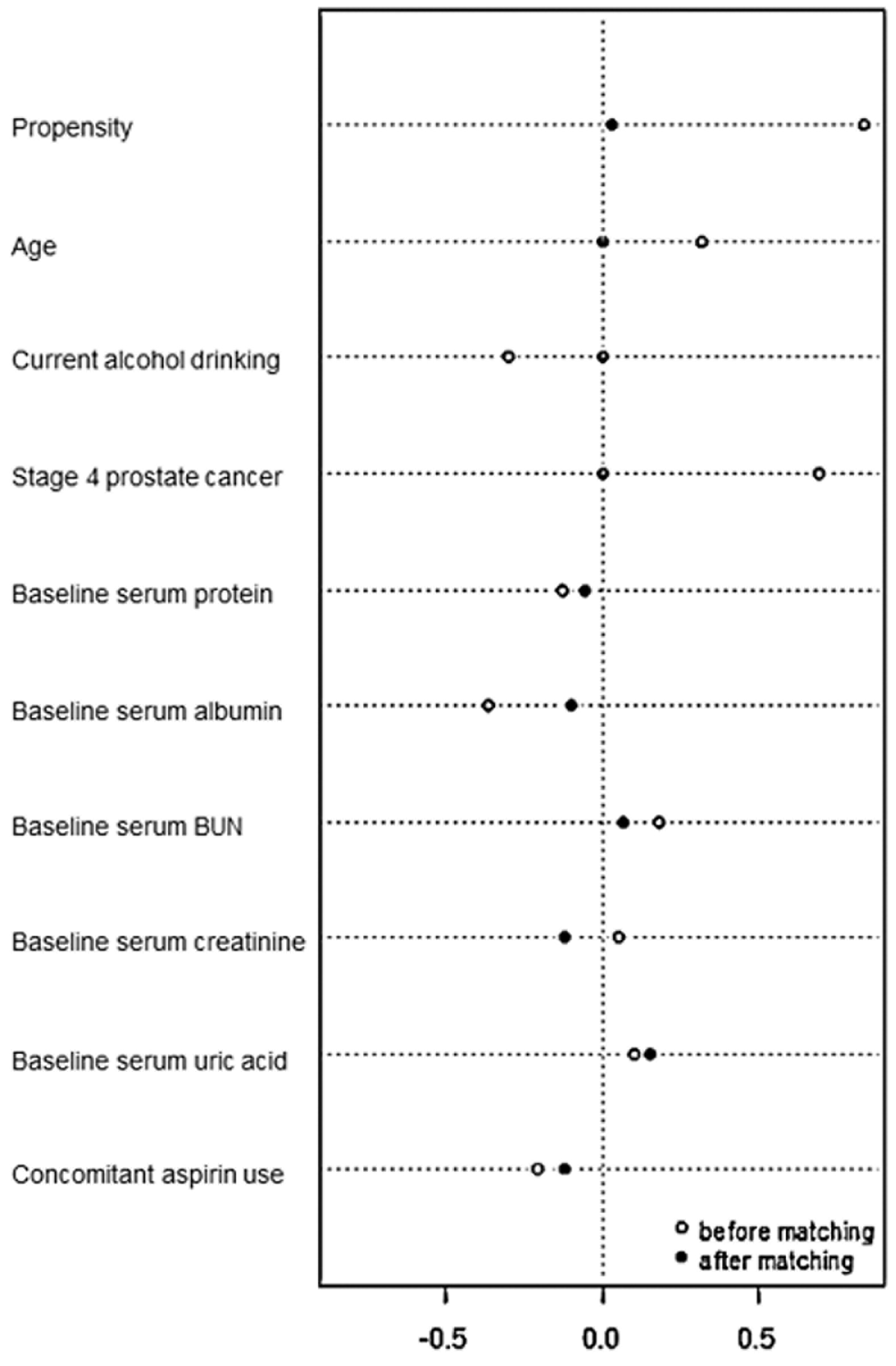

Supplement: S1 Fig — (TIF) [file pone.0209049.s002.tif]
