# Supplementary material for: Influence of androgen deprivation therapy on serum urate levels in patients with prostate cancer: A retrospective observational study
Source: PLoS One. 2018 Dec 17;13(12):e0209049. doi: 10.1371/journal.pone.0209049 (PMC6296534; doi:10.1371/journal.pone.0209049)
Supplement: S1 Table — AR subgroup received monotherapy with AR antagonists. (DOCX) [file pone.0209049.s003.docx]

**Supporting information files.**

**S1 Table.** Baseline features of luteinizing hormone-releasing hormone (LHRH) agonist-positive and negative subgroups. LHRH agonist-negative subgroup received a monotherapy with androgen receptor antagonists.

|  | LHRH agonist-positive subgroup  (n = 114) | LHRH agonist-negative subgroup  (n = 36) | P value |
| --- | --- | --- | --- |
| Age, year, mean (SD) | 69.7 (7.5) | 67.4 (5.9) | 0.081 |
| Body mass index, kg/m^2^, mean (SD) | 24.3 (2.6) | 24.5 (2.4) | 0.703 |
| Current alcohol drink^*^, n (%) | 42 (38.9) | 12 (33.3) | 0.551 |
| Hypertension, n (%) | 58 (50.9) | 19 (52.8) | 0.842 |
| Diabetes mellitus, n (%) | 22 (19.3) | 6 (16.7) | 0.724 |
| Coronary artery disease, n (%) | 4 (3.5) | 37 (8.3) | 0.237 |
| Dyslipidemia, n (%) | 22 (19.5) | 9 (25.0) | 0.476 |
| Concomitant radiotherapy, n (%) | 42 (36.8) | 31 (86.1) | <0.001 |
| Metastatic prostate cancer, n (%) | 45 (39.5) | 4 (11.1) | 0.002 |
| ECOG functional status^†^, n (%) |  |  | 0.006 |
| 0 | 9 (8.9) | 2 (5.6) |  |
| 1 | 63 (62.4) | 34 (94.4) |  |
| 2 | 26 (25.7) | 0 (0.0) |  |
| 3 | 2 (2.0) | 0 (0.0) |  |
| 4 | 1 (1.0) | 0 (0.0) |  |
| Serum uric acid, mg/dL, mean (SD) | 5.67 (1.39) | 6.02 (1.13) | 0.169 |
| Hyperuricemia^¶^, n (%) | 24 (21.1) | 13 (36.1) | 0.068 |
| Hypouricemia^‡^, n (%) | 12 (10.5) | 0 (0.0) | 0.071 |
| Serum protein, mg/dL, mean (SD) | 7.1 (0.7) | 7.1 (0.4) | 0.525 |
| Serum albumin, mg/dL, mean (SD) | 4.1 (4.3) | 4.2 (4.0) | 0.249 |
| Serum cholesterol, mg/dL, mean (SD) | 175.6 (36.9) | 180,0 (33.2) | 0.525 |
| BUN, mg/dL, mean (SD) | 16.0 (4.4) | 17.2 (4.0) | 0.165 |
| Serum creatinine, mg/dL, mean (SD) | 0.96 (0.16) | 0.92 (0.13) | 0.158 |
| Medications |  |  |  |
| Aspirin use, n (%) | 21 (18.4) | 4 (11.1) | 0.305 |
| Thiazide, n (%) | 10 (8.8) | 3 (8.3) | 0.924 |
| Loop diuretics, n (%) | 2 (1.8) | 1 (2.8) | 0.702 |
| Angiotensin receptor blockers, n (%) | 31 (27.4) | 5 (13.9) | 0.098 |
| Statins, n (%) | 26 (23.0) | 10 (27.8) | 0.560 |

*, data were missing in 6; †, data were missing in 13; ¶, serum urate level ≥ 7.0 mg/dL; ‡, serum urate level < 4.0 mg/dL; BUN, blood urea nitrogen; ECOG, Eastern Cooperative Oncology Group; SD, standard deviation.
