# Supplementary material for: Influence of androgen deprivation therapy on serum urate levels in patients with prostate cancer: A retrospective observational study
Source: PLoS One. 2018 Dec 17;13(12):e0209049. doi: 10.1371/journal.pone.0209049 (PMC6296534; doi:10.1371/journal.pone.0209049)
Supplement: S2 Table — (DOCX) [file pone.0209049.s004.docx]

**S2 Table.** Baseline characteristics of the propensity-score–matched patients

|  | Surgery group  (n = 90) | ADT group  (n = 90) | P value |
| --- | --- | --- | --- |
| Age^*^, year, mean (SD) | 68.0 (6.4) | 68.9 (6.7) | 0.401 |
| Body mass index, kg/m^2^, mean (SD) | 24.5 (2.8) | 24.7 (2.5) | 0.647 |
| Current alcohol drink^*^, n (%) | 42 (46.7) | 33 (36.7) | 0.174 |
| Hypertension, n (%) | 43 (47.8) | 46 (51.1) | 0.655 |
| Diabetes mellitus, n (%) | 14 (15.6) | 15 (16.7) | 0.839 |
| Coronary artery disease, n (%) | 6 (6.7) | 3 (3.3) | 0.305 |
| Dyslipidemia, n (%) | 22 (24.2) | 23 (25.6) | 0.863 |
| Concomitant radiotherapy, n (%) | 2 (2.2) | 56 (62.2) | <0.001 |
| Metastatic prostate cancer^*^, n (%) | 2 (2.2) | 2 (2.2) | 1.000 |
| ECOG functional status, n (%) |  |  | <0.001 |
| 0 | 16 (17.8) | 4 (4.4) |  |
| 1 | 40 (44.4) | 69 (76.7) |  |
| 2 | 34 (37.8) | 16 (17.8) |  |
| 3 | 0 (0.0) | 1 (1.1) |  |
| 4 | 0 (0.0) | 0 (0.0) |  |
| Serum uric acid^*^, mg/dL, mean (SD) | 5.43 (1.30) | 5.84 (1.17) | 0.027 |
| Hyperuricemia^†^, n (%) | 12 (13.3) | 16 (17.8) | 0.411 |
| Hypouricemia^¶^, n (%) | 15 (16.7) | 3 (3.3) | 0.003 |
| Serum protein^*^, mg/dL, mean (SD) | 7.2 (0.6) | 7.1 (0.6) | 0.128 |
| Serum albumin^*^, mg/dL, mean (SD) | 4.2 (0.5) | 4.1 (0.4) | 0.110 |
| Serum cholesterol, mg/dL, mean (SD) | 182.1 (35.0) | 184.2 (31.1) | 0.676 |
| BUN^*^, mg/dL, mean (SD) | 15.5 (3.5) | 15.7 (3.1) | 0.786 |
| Serum creatinine^*^, mg/dL, mean (SD) | 0.95 (0.17) | 0.95 (0.13) | 0.984 |
| Medications |  |  |  |
| Aspirin use, n (%) | 14 (15.6) | 12 (13.3) | 0.672 |
| Thiazide, n (%) | 10 (11.1) | 8 (8.9) | 0.619 |
| Loop diuretics, n (%) | 0 (0.0) | 2 (2.2) | 0.155 |
| Angiotensin receptor blockers, n (%) | 29 (32.2) | 21 (23.3) | 0.183 |
| Statins, n (%) | 25 (27.8) | 23 (25.60) | 0.736 |

*, variables were used in propensity score matching; †, serum urate level ≥ 7.0 mg/dL; ¶, serum urate level < 4.0 mg/dL; ADT, androgen deprivation therapy; ARB, angiotensin receptor blocker; BUN, blood urea nitrogen; ECOG, Eastern Cooperative Oncology Group; SD, standard deviation.
